# Hierarchical Targeting Nanodrug with Holistic DNA Protection for Effective Treatment of Acute Kidney Injury

**DOI:** 10.1002/advs.202411254

**Published:** 2024-12-20

**Authors:** Qiaohui Chen, Yongqi Yang, Xiaohong Ying, Changkun Huang, Jianlin Chen, Jue Wang, Ziyu Wu, Wan Zeng, Chenxi Miao, Xiaojing Shi, Yayun Nan, Qiong Huang, Kelong Ai

**Affiliations:** ^1^ Department of Pharmacy Xiangya Hospital Central South University Changsha 410008 China; ^2^ Xiangya School of Pharmaceutical Sciences Central South University Changsha 410013 China; ^3^ Hunan Provincial Key Laboratory of Cardiovascular Research Xiangya School of Pharmaceutical Sciences Central South University Changsha 410013 China; ^4^ Department of Urology The Second Xiangya Hospital Central South University Changsha Hunan 410011 China; ^5^ Department of Pancreatic Surgery Xiangya Hospital Central South University Changsha 410008 China; ^6^ Department of General Surgery Xiangya Hospital Central South University Changsha 410008 China; ^7^ Geriatric Medical Center People's Hospital of Ningxia Hui Autonomous Region Yinchuan Ningxia 750002 China; ^8^ National Clinical Research Center for Geriatric Disorders Xiangya Hospital Central South University Changsha 410008 China; ^9^ Key Laboratory of Aging‐related Bone and Joint Diseases Prevention and Treatment Ministry of Education Xiangya Hospital Central South University Changsha 410008 China

**Keywords:** AKI, antioxidative nanomedicine, mitochondria, nucleus, oxidative stress, subcellular organelle targeting

## Abstract

Acute kidney injury (AKI) manifests a hallmark pathological feature of extensive and severe DNA damage in renal tubules, primarily induced by the excessive of toxic reactive oxygen species (ROS) from the mitochondrial electron transport chain. The kidney's complex intricate physiological architecture and the heterogeneous intracellular environment pose significant challenges for effective sequential and high‐resolution drug delivery—an urgent issue that remains unresolved. To address this, a hierarchical‐targeting antioxidant nanodrug has been developed with a folic acid moiety (HAND) designed for high‐resolution drug delivery in AKI treatment. For the first time, HAND enables sequential targeting from the kidney to the most severely damaged proximal tubular epithelial cells (PTECs), ultimately concentrating in the DNA‐rich mitochondria and nucleus. As a result, HAND effectively scavenges ROS in situ, protecting both mitochondria and nuclei along with their vital genetic material. This action restores mitochondrial function, mitigates DNA oxidation and fragmentation, reduces apoptosis, and inhibits cGAS/STING‐mediated sterile inflammation. Consequently, HAND demonstrates remarkable efficacy in safeguarding injured kidneys during AKI. Overall, this work pioneers a hierarchical, high‐resolution antioxidant strategy, providing innovative guidance for the development of AKI therapies.

## Graphical abstract

This study developed a hierarchical‐targeting antioxidant nanodrug (HAND) for precise AKI treatment, achieving targeted delivery from kidney to severely damaged proximal tubular epithelial cells, and concentrating in DNA‐rich mitochondria and nucleus. HAND effectively scavenges ROS in situ, protecting vital genetic material and demonstrating significant efficacy in preserving kidney function during AKI.

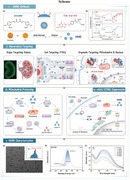

## 1 Introduction

Acute kidney injury (AKI) refers to the sudden deterioration of kidney function, primarily due to acute dysfunction of proximal tubular epithelial cells (PTECs).^[^ ^1^ ^]^ Globally, AKI affects over 13 million individuals annually and is particularly prevalent among hospitalized patients, with incidence rates reaching 30%−60% in critical care settings.^[^ ^2^ ^]^ Unfortunately, no pharmacological interventions have been successfully demonstrated to provide renal protection in AKI. Supportive care and renal replacement therapy are currently the only clinical treatment options,^[^ ^3^ ^]^ but their effectiveness is limited, and they impose a significant burden on healthcare systems. If untreated or inadequately managed, AKI can lead to renal failure and progress toward kidney fibrosis, driven by sustained oxidative stress and chronic inflammation, ultimately resulting in severe end‐stage renal disease, multi‐organ failure, or death.^[^ ^4^ ^]^ With an unacceptably high mortality rate of 1.7 million deaths annually, AKI presents a significant clinical challenge and underscores the urgent need for novel drug development.^[^ ^5^, ^6^ ^]^

AKI is marked by a defining pathological feature: extensive and severe DNA damage in renal tubules.^[^ ^7^, ^8^ ^]^ DNA, the repository of genetic information, is located exclusively in the nucleus and mitochondria of human cells, orchestrating cellular structure, function, and responses to stimuli through gene expression regulation.^[^ ^9^ ^]^ Damage to DNA disrupts this regulatory function and collapses the cellular command center, directly resulting in cell death.^[^ ^10^ ^]^ Moreover, irreparable DNA fragments released into the cytoplasm or extracellular space act as critical signals that activate innate DNA sensing pathways, triggering inflammatory cascades and cytokine storms.^[^ ^11^ ^]^ Consequently, the loss of functional cells combined with persistent inflammation significantly impairs renal function, leading to acute manifestations such as electrolyte imbalances, fluid overload, and acidosis, as well as the malignant progression of fibrosis.^[^ ^12^ ^]^ The primary source of DNA damage in AKI is endogenous oxidative stress, particularly from the production of toxic reactive oxygen species (ROS) by the mitochondrial electron transport chain (ETC).^[^ ^13^ ^]^ The kidneys, as one of the most mitochondria‐rich organs, receive 20% of cardiac output and utilize 10% of the body's oxygen.^[^ ^14^ ^]^ To support the intense transport and reabsorption functions in PTECs, mitochondria, serving as cellular power factories, are densely concentrated within these cells.^[^ ^15^ ^]^ Stimulants of AKI, such as rhabdomyolysis, systemic inflammation, or ischemia/reperfusion, trigger electron leakage from the mitochondrial ETC in PTECs, leading to a surge in mitochondrial ROS (mtROS).^[^ ^16^ ^]^ Critically, the high mitochondrial density within PTECs allows mtROS to rapidly diffuse through the mitochondrial pool, creating a storm that engulfs neighboring mitochondria and the nucleus, the latter of which is closely and fully surrounded by these mitochondria.^[^ ^17^ ^]^ As highly nucleophilic species with unpaired electrons, ROS engage in intense chemical reactions with electrophilic biomolecules, including DNA.^[^ ^18^ ^]^ These interactions result in substantial oxidative double‐stranded DNA damage, which subsequently activate cell death pathways and inflammatory signaling mediated by the cGAS/STING axis.^[^ ^19^ ^]^ Thus, the damage inflicted by ROS on the two crucial DNA‐rich organelles in PTECs—mitochondria and the nucleus—propagates rapidly, akin to a cascading domino effect, ultimately compromising the entire kidney.

Therefore, effectively neutralizing excessive toxic ROS represents a crucial therapeutic objective to mitigate the source of damage and protect PTECs, ultimately improving renal function. However, clinically utilized antioxidant molecules, such as amifostine and N‐acetylcysteine (NAC), offer limited protection against AKI due to their lack of targeting capability and poor bioavailability. In recent years, emerging nanomedicine has demonstrated a remarkable trajectory of advancement,^[^ ^20^, ^21^ ^]^ with several mitochondria‐specific targeting nanostrategies under active investigation.^[^ ^22^, ^23^ ^]^ Nonetheless, these approaches face significant challenges in achieving effective and precise treatment for AKI. First, the presence of positive charges and/or hydrophobic structures in mitochondria‐targeting compounds limits their stability and in vivo applicability, as negatively charged enzymes or proteins in circulation may interact with them, thereby diminishing the targeting efficacy.^[^ ^24^ ^]^ Additionally, complex encapsulation or modification can result in increased particle size, complicating passage through the glomerular filtration barrier (GFB) and raising safety concerns related to residual materials.^[^ ^25^ ^]^ Second, some nanomaterials that are appropriately sized for the GFB often fail to effectively target PTECs, the primary site of injury in AKI, leading to a rapid loss of therapeutic efficacy as they are swiftly excreted in the urine. The nucleus, serving as the cell's primary command center, is often overlooked by researchers, leading to inadequate protection. This neglect is particularly concerning, given the close spatial and functional interconnections between mitochondria and the nucleus,^[^ ^26^ ^]^ both of which are highly susceptible to ROS. Thus, developing a hierarchical targeting strategy for the precise delivery of therapeutic agents to kidney‐PTECs‐mitochondria and the nucleus—facilitating targeted ROS clearance at the tissue, cellular, and subcellular levels—holds significant promise for innovative and effective treatment of AKI. However, the unique physiological structure of the kidney and the complex, heterogeneous intracellular environment present formidable challenges to achieving such sequential and high‐precision targeting. As a result, no practical applications from this perspective have been reported to date.

**Scheme 1:**
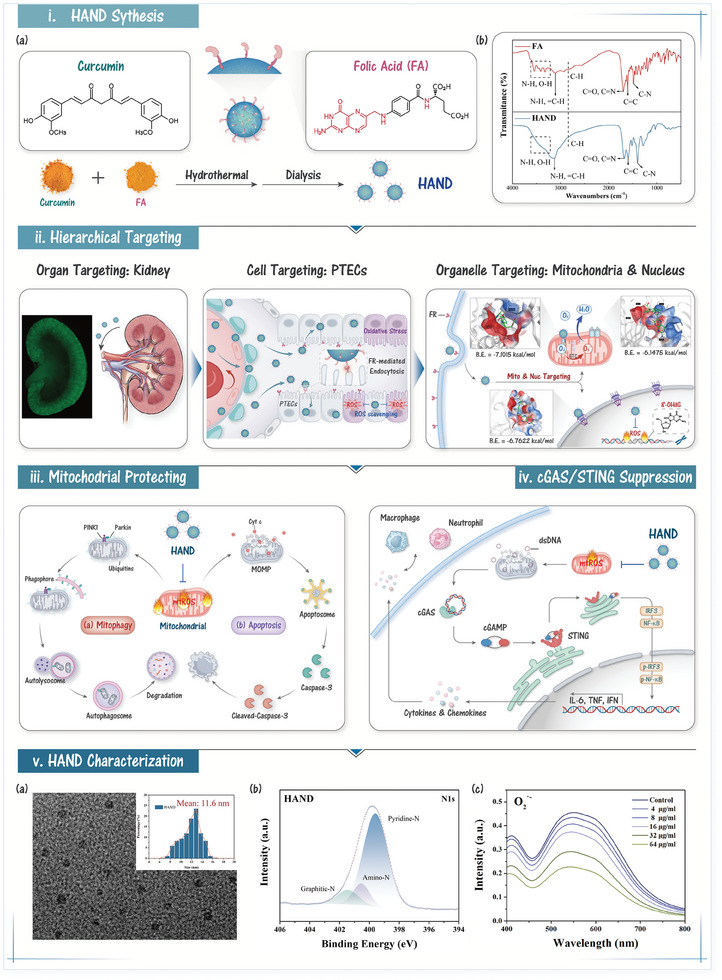
Hierarchical Targeting Nanodrug with Holistic DNA Protection for Effective Treatment of Acute Kidney Injury. i) HAND was synthesized using curcumin and FA as precursor compounds through a hydrothermal method. Infrared spectroscopy results indicated that HAND successfully inherited the surface groups of FA. ii) Upon intravenous injection, ultrasmall HAND could passively transverse the GFB and be actively taken up by PTECs through FR‐mediated endocytosis. Furthermore, HAND could localize in the nucleus and mitochondria due to the high affinity of FA for their surface membrane proteins. iii) HAND effectively protected mitochondrial structure and function via scavenging mtROS, subsequently inhibiting damage‐induced mitophagy and intrinsic apoptosis. iv) Through effectively scavenging mtROS, HAND could reduce the release of dsDNA, thereby suppressing sterile inflammation caused by cGAS/STING pathway activation. v) TEM image of HAND (a). Insert: Size distribution of HAND. XPS N1s spectrum of HAND (b). UV absorption spectra of O_2_^·−^ reacting with different concentrations of HAND.

To address this challenge, a portable and elegant hierarchical‐targeting antioxidant nanodrug (HAND) derived from folic acid (FA) and curcumin (**Scheme** **1**i) has been developed for precise delivery to the mitochondria and the nucleus in PTECs, which enabled the specific and highly efficient clearance of toxic mtROS and mitigation of DNA damage. Curcumin, a lipophilic polyphenol compound extracted from turmeric rhizomes, has demonstrated a wide range of pharmacological activities, including antioxidant, antimicrobial, and anti‐inflammatory properties. Moreover, curcumin was recognized as a “generally regarded as safe” compound by the U.S. Food and Drug Administration. However, its therapeutic efficacy in treating AKI is limited by poor bioavailability, rapid metabolism, and low water solubility when used alone. Fortunately, the chemical structure of curcumin, characterized by abundant sp2 and sp3 carbon atoms along with antioxidant functional groups such as phenolic hydroxyl and methoxy groups, makes it an ideal precursor for synthesizing carbon dots with antioxidant properties. In this study, we have integrated curcumin with the targeting molecule FA into integrated carbon dots──HAND, not only enhancing curcumin's bioavailability but also promoting its precise delivery to AKI lesions. The ultrasmall HAND is abundant in FA‐like groups and possesses a strong negative charge on its surface. Therefore, HAND was less susceptible to sequestration by the hepatic reticuloendothelial system, facilitating its accumulation in the kidneys and enabling it to penetrate the GFB to reach the renal tubular lumen. Furthermore, PTECs expressed high levels of FA receptors (FR) on their surface, granting them a high affinity for HAND and facilitating efficient endocytosis. Notably, we have identified, for the first time, that FA demonstrates a strong affinity for translocases of the outer and inner mitochondrial membrane (TOM complex and TIM23 complex) and FG nucleoporins (FG Nups) within nuclear pore complexes. This property is effectively inherited by HAND, which shows pronounced co‐localization with both the nucleus and mitochondria in human renal tubular epithelial cells (HK‐2 cells). Therefore, HAND can actively target PTECs and preferentially accumulate in DNA‐rich regions (Scheme 1ii). By leveraging its abundant antioxidant groups, HAND efficiently scavenged toxic mtROS, thereby protecting the mitochondria and nucleus, restoring mitochondrial function, mitigating apoptosis, and attenuating cGAS/STING‐mediated sterile inflammation (Scheme 1iii,iv). This multifaceted approach effectively safeguarded PTECs and facilitated the restoration of renal function in AKI mouse models. Moreover, HAND, derived from natural products (curcumin) and essential nutrients (FA), exhibited commendable biocompatibility and was effectively eliminated from the body through renal clearance within 72 h. In conclusion, this study introduces a high‐resolution, hierarchical targeting antioxidant therapeutic strategy, providing a new paradigm for the development of therapeutic agents for renal diseases, including but not limited to AKI.

## 2 Results

### 2.1 Synthesis and Characterization of HAND

In this study, we synthesized HAND and CND (without FA) by inducing a carbonization process using FA and curcumin as carbon source precursors at 240 °C (Scheme 1i–a). Transmission electron microscopy (TEM) confirmed that HAND and CND were monodisperse, spherical nanoparticles with an average diameter of ≈11 nm (Scheme 1v–a; Figure S1, Supporting Information), well below the particle size threshold for GFB (≈14 nm). The surface charges of HAND and CND were −34.5 and −23.5 mV, respectively, supporting their stable dispersion in circulation (Figure S2, Supporting Information). We subsequently used X‐ray photoelectron spectroscopy (XPS) to analyze the composition of HAND. The XPS spectrum of HAND showed three main peaks: C1s (285 eV), N1s (400 eV), and O1s (534 eV) (Figure S3, Supporting Information), while CND exhibited only two peaks: C1s and O1s (Figure S4, Supporting Information), indicating that HAND inherited nitrogen from FA. As illustrated in Scheme 1v–b, the high‐resolution XPS N1s spectrum of HAND identified three distinct peaks attributed to pyridinic nitrogen (400.5 eV), amino nitrogen (401.6 eV), and graphitic nitrogen (402.6 eV). Concurrently, the XPS C1s spectrum (Figure S5, Supporting Information) of HAND displayed five distinct peaks corresponding to C─C, C═C, C─N/C─O, C═O, and π–π^*^ transitions, suggesting a nitrogen‐doped carbon framework with FA‐derived surface groups. Fourier‐transform infrared (FT–IR) spectroscopy further supported these findings, revealing N‐containing absorption peaks such as ─NH, C═N, and C─N, consistent with FA, alongside typical carbon dot peaks for ─OH, C─H, C═C, and C─O (Scheme 1i–b). In contrast, CND showed only characteristic carbon dot peaks for ─OH, C─H, C═O (1638 cm⁻¹), C═C, and C─O (Figure S6, Supporting Information). UV–vis spectroscopy of HAND and CND revealed two major absorption bands at 277 and 300–400 nm, corresponding to π–π^*^ transitions of polycyclic aromatic chromophores (C═C) and n‐π^*^ transitions of C─N/C═O, respectively (Figure S7, Supporting Information). These results confirmed the successful synthesis of HAND, which exhibited an N‐doped conjugated carbon framework with an abundant surface group derived from FA and curcumin as expected. Consequently, these surface groups, including methoxy, phenolic hydroxyl, and amino groups inherited from FA and curcumin, endowed HAND with exceptional and broad‐spectrum ROS scavenging capacity. For example, HAND effectively neutralized O_2_^·⁻^ in vitro, counteracting the primary ROS generated by mitochondrial ETC electron leakage, thus potentially limiting mtROS propagation (Scheme 1v–c). As a reductive sacrificial agent, HAND also effectively detoxified H₂O₂, a longer‐lived ROS, and secondary radicals derived from O_2_^·⁻^, such as ·OH and ONOO^⁻^ (Figure S8, Supporting Information). Similarly, CND demonstrated comparable ROS scavenging activity due to the antioxidant groups inherited from curcumin (Figure S9, Supporting Information). Thus, HAND's broad‐spectrum antioxidant activity as a reductive sacrificial agent was robustly validated. In conclusion, HAND was successfully synthesized and comprehensively characterized, confirming its expected structure and potent antioxidant properties. With its ultra‐small size and potential for active targeting to PTECs, HAND holds considerable promise for renal accumulation, which is evaluated in subsequent animal models.

### 2.2 Hierarchical Targeting of HAND: from Kidney to PTECs, and Ultimately to Mitochondria and Nucleus

**Figure 1:**
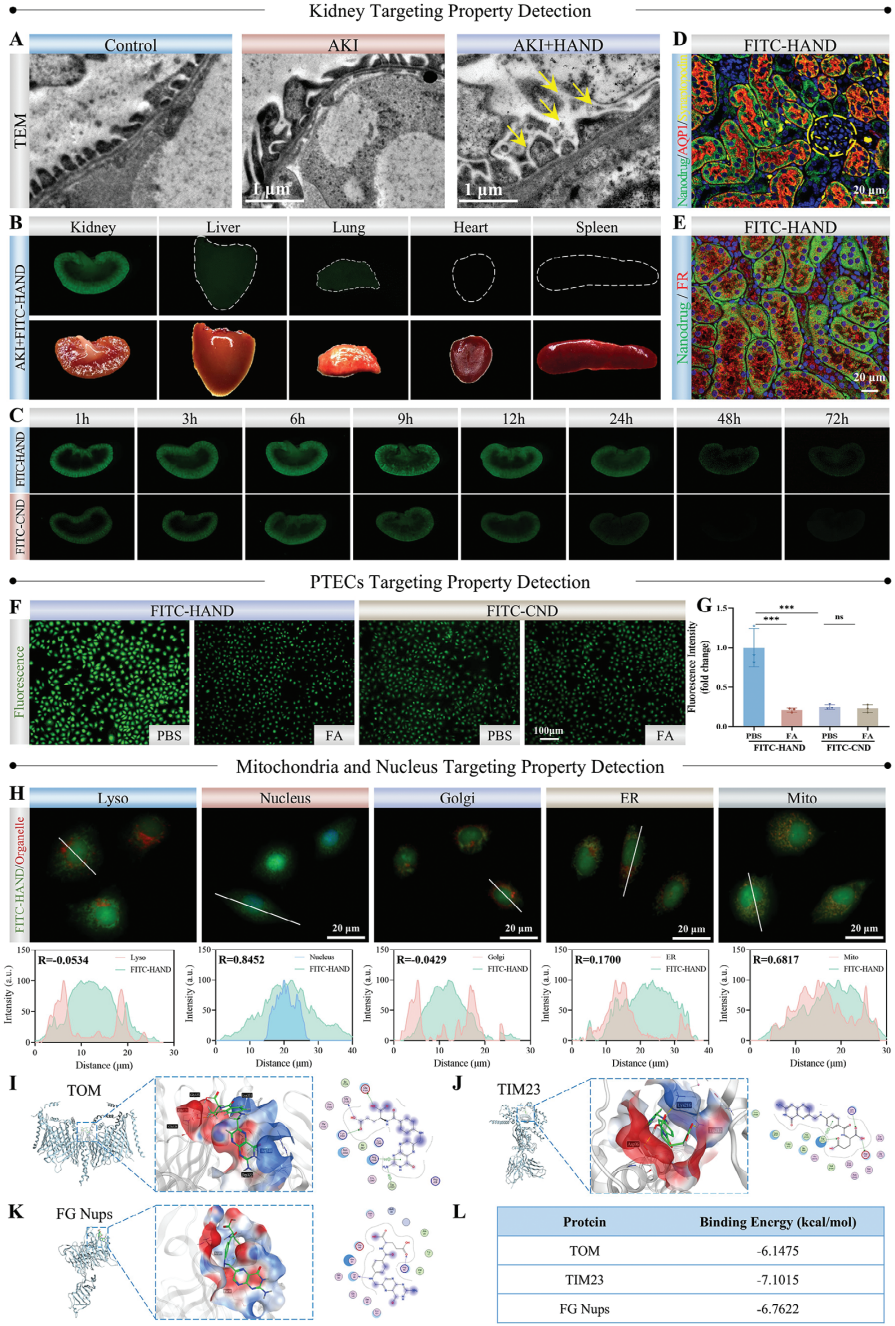
The hierarchical targeting property of HAND. A) TEM images of GFB in different groups. B) Representative fluorescence images of different organs in AKI mice 6 h after intravenous injection of HAND. C) Representative fluorescence images of kidneys in AKI mice at various time points (1‐72 h) after intravenous injection of HAND. D) Representative IF staining images of FITC‐HAND with glomerular (synaptopodin) and tubular (AQP1) markers in mice kidney tissues, with yellow circles indicating glomeruli. E) Representative IF staining image of FITC‐HAND with FR in mice kidney tissues. F,G) Representative fluorescence images (F) and quantitative fluorescence analysis (G) of HK‐2 cells incubated with FITC‐HAND or FITC‐CND in different groups. H) Colocalization fluorescence images and correlation coefficient analysis of FITC‐HAND with different organelles in HK‐2 cells. I–L) Molecular docking results of FA with TOM comple, TIM23, and FG Nups. Data are presented as mean ± SD. One‐way ANOVA followed by the Student–Newman–Keuls (SNK) test was used for analysis. *n* = 3, ^***^ *p* < 0.001, ^****^ *p* < 0.0001; ^##^ *p* < 0.01, ^###^ *p* < 0.001; ns, not significant (*p* > 0.05).

To investigate the hierarchical targeting properties of HAND, a rhabdomyolysis‐induced AKI (RM‐AKI) mouse model was established by injecting equal volumes of 50% glycerol into both hind limbs. RM‐AKI, commonly encountered in trauma and perioperative contexts, represents one of the most prevalent forms of AKI in clinical practice. Following intravenous administration of HAND to the AKI mice, TEM images of the glomerulus were captured to assess the passage of HAND through the GFB. As shown in **Figure** **1**A, HAND could be directly observed in the glomerular capsule, confirming its successful traversal of the GFB. To further track their distribution in vivo, HAND and CND were labeled with fluorescein isothiocyanate to form FITC‐HAND and FITC‐CND, which showed comparable particle size and surface charge to HAND and CND (Figure S10, Supporting Information). The labeling process involved the reaction of the isothiocyanate group of FITC with the amino and hydroxyl functional groups present in HAND and CND, forming stable covalent bonds (Figure S11, Supporting Information). The resulting conjugates were then visualized through stereo‐fluorescence microscopy to assess their distribution and localization in biological systems. As shown in Figure 1B and Figures S12,S13 (Supporting Information), FITC‐HAND and FITC‐CND demonstrated significant renal accumulation in both Control and AKI mice, with minimal distribution in the liver and lungs, and negligible presence in the heart and spleen. Such in vivo pharmacokinetic behavior of HAND and CND can be attributed to their negative charge, ultra‐small size, and excellent water solubility. We then further analyzed the temporal variation in renal concentrations of HAND and CND. Within 1 h post‐injection, a distinct green fluorescence was observed in the kidneys of the AKI+FITC‐HAND and AKI+FITC‐CND groups, with fluorescence intensity increasing over time, peaking ≈6 h before gradually declining due to drug excretion (Figure 1C; Figures S14,S15, Supporting Information). Notably, the peak fluorescence intensity (6 h) and fluorescence duration (24 h) of FITC‐HAND in the kidneys were significantly greater than those of FITC‐CND (Figure S16, Supporting Information). This sustained accumulation highlighted the superior of HAND in actively targeting the focus, ensuring more specific and prolonged ROS scavenging in PTECs, as opposed to merely retaining in the tubular lumen or being rapidly excreted in urine. The enhanced specific and sustained accumulation of HANDs in the kidneys compared with CNDs may be attributed to surface groups inherited from FA, which specifically bind to FR‐rich PTECs. Meanwhile, HAND was labeled with a Cy7 probe and administered to mice for in vivo imaging. The results showed that once in circulation, HAND rapidly accumulated in the kidneys, demonstrating time‐dependent uptake that peaked at 6 h before gradually declining. This accumulation pattern is well‐aligned with stereo fluorescence imaging results, providing strong evidence of the targeted renal distribution of HAND. Notably, the distribution of HAND in other organs, such as the liver, was relatively low, further supporting its kidney‐specific targeting capability (Figure S17, Supporting Information).

Thereafter, we further delved into the influence of FA‐derived groups on the more microcosmic targeting properties of HANDs at the cellular and organellar levels. FA, an essential micronutrient, is primarily regulated by the kidneys to maintain systemic homeostasis.^[^ ^27^ ^]^ Upon entering circulation, FA is freely filtered through the glomerulus, with most being reabsorbed by PTECs to prevent excretion. The reabsorption process relies heavily on the FR located on the brush border of PTECs.^[^ ^28^ ^]^ Our immunofluorescence (IF) staining of human kidney samples confirmed that PTECs express high levels of FR, establishing a foundation for the cell‐specific targeting of FA‐functionalized HAND (Figure S18, Supporting Information). As expected, the ultrasmall FITC‐HAND and FITC‐CND were able to cross the GFB, primarily distributing within renal tubules (marked by AQP1) rather than glomeruli (marked by synaptopodin) in mouse kidney tissues (Figure 1D; Figure S19, Supporting Information). Importantly, FITC‐HAND derived from FA showed a high degree of colocalization with FR on the surface of PTECs, which was significantly greater than that of FITC‐CND, highlighting the active targeting capability of HAND toward FR (Figure 1E; Figure S20, Supporting Information). We also verified HAND's FR‐targeting ability at the cellular level by comparing HAND uptake across various cell lines with differing FR expression. As shown in Figure S21 (Supporting Information), FITC‐HAND was effectively and abundantly taken up by FR‐high‐expressing HK‐2 cells, while its uptake by FR‐low‐expressing cardiomyocytes (H9c2) and hepatic stellate cells (LX2) was lower under same conditions. In contrast, FITC‐CND exhibited no significant differences in uptake among these cell lines. Notably, pre‐treatment with FA significantly inhibited FITC‐HAND uptake in HK‐2 cells, evidenced by a marked decrease in intracellular fluorescence (Figure 1F,G). This competitive inhibition indicates that HAND internalization is primarily FR‐mediated. Conversely, under the same conditions, FITC‐CND exhibited markedly lower fluorescence intensity in HK‐2 cells compared to FITC‐HAND, and FA pre‐treatment did not affect its uptake. Together, these findings underscore the FR‐dependent targeting capability of HAND. Furthermore, FA is a pivotal component of intracellular one‐carbon metabolism, exhibiting a compartmentalized pattern of utilization, predominantly within the mitochondria and nucleus. Such compartmentalization likely drives the selective intracellular localization of HAND. Specifically, HAND exhibited a pronounced dual‐targeting specificity for the nucleus and mitochondria in HK‐2 cells, with Pearson's correlation coefficient of 0.85 and 0.68, respectively, corresponding to the primary compartments involved in FA‐mediated one‐carbon metabolism (Figure 1H). To further elucidate the targeting and adhesion mechanisms of HAND, we employed molecular docking to assess FA's affinity for mitochondrial and nuclear proteins. TOM and TIM are critical macromolecular complexes embedded in the outer and inner mitochondrial membranes, respectively, facilitating the localization and import of nuclear‐encoded proteins into mitochondria.^[^ ^29^ ^]^ The nuclear pore complex (NPC) serves as the principal gateway for macromolecular transport between the nucleus and cytoplasm. NPC is highly conserved and intricate, with FG Nups in its central channel mediating the interactions with cargo.^[^ ^30^ ^]^ Molecular docking results indicated that FA achieved high docking scores with TOM, TIM, and FG Nups due to its molecular flexibility, stable conformation, and effective non‐bonded interactions, with calculated free binding energies of −6.15, −7.10, and −6.76 kcal mol^−1^, respectively (Figure 1I–L). To further validate the affinity of FA‐mediated HAND for TOM, TIM, and FG Nups, we pre‐treated HK‐2 cells with FA before incubation with FITC‐HAND and examined its the colocalization of FITC‐HAND with various organelles. As illustrated in Figure S22 (Supporting Information), pre‐treatment with FA significantly reduced the distribution of HAND in the mitochondria and nucleus, with co‐localization coefficients decreasing to 0.37 and 0.42, respectively. In contrast, there were no significant changes in the co‐localization of HAND with the endoplasmic reticulum (ER), Golgi apparatus, and lysosomes (Lyso), confirming that the dual‐targeting capability of HAND is mediated by FA. These findings provided compelling validation and mechanistic insights into the distinct dual‐targeting affinity of HAND for both mitochondria and the nucleus.

In summary, the above evidence strongly demonstrated the hierarchical targeting properties of HAND: sequentially from the kidney to PTECs, and ultimately to the mitochondria and nucleus, which is characterized by high and persistent accumulation in the kidneys, specific binding to PTECs, and subsequent precise localization targeting to mitochondria and nucleus.

### 2.3 HAND Protected the Kidneys of AKI Mice by Suppressing Oxidative Stress

**Figure 2:**
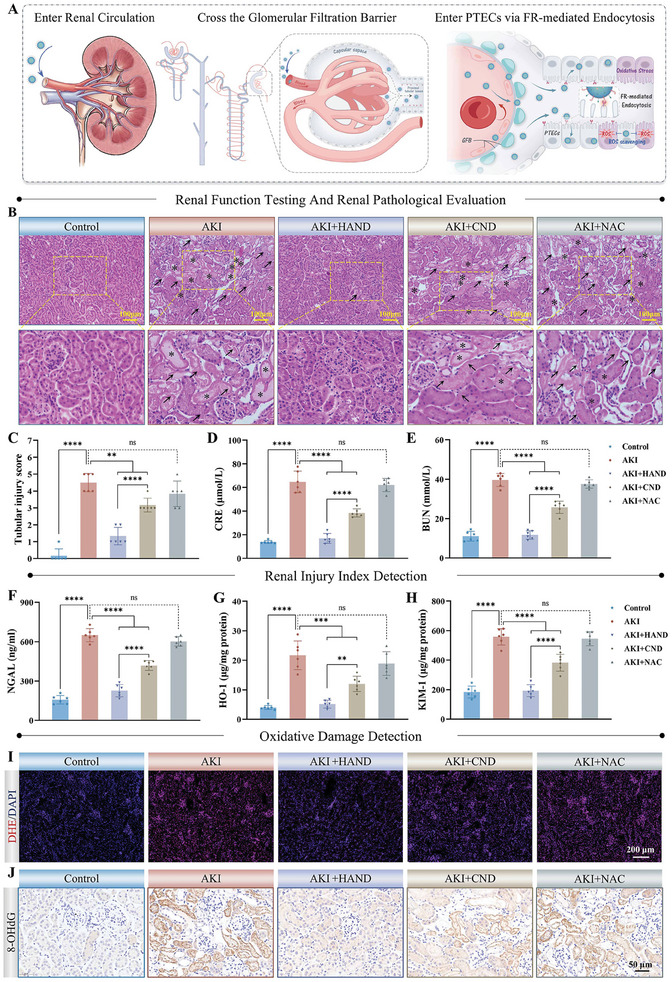
HAND protected the kidneys of AKI mice by suppressing oxidative stress. A) Schematic illustration of HAND crossing the GFB and actively targeting PTECs. B) Representative H&E‐stained images of kidney tissues from different treatment groups. Arrows indicate damaged PTECs, and asterisks indicate casts. C–E) Kidney injury scores (C), levels of serum CRE (D), and BUN (E) in different treatment groups. F–H) Levels of NGAL (F), HO‐1 (G), and KIM‐1 (H) in kidney tissues of different treatment groups. I) Representative DHE fluorescence images of kidney tissues in different treatment groups. J) IHC results of 8‐OHdG in mouse kidneys from different treatment groups. Data are presented as mean ± SD. One‐way ANOVA followed by the SNK test was used for analysis. *n* = 6, ^**^ *p* < 0.01, ^***^ *p* < 0.001, ^****^ *p* < 0.0001; ns, not significant (*p* > 0.05).

Given its hierarchical targeting (**Figure** **2**A) and potent antioxidant properties, the in vivo therapeutic effect of HAND on AKI was further explored in RM‐AKI mice. Renal tubular injury scores and renal function indicators are standard criteria for assessing the extent of renal damage in AKI. The renal tubular injury score, ranging from 0 to 5, reflects the severity of renal damage based on renal tubule morphology, with higher scores indicating more severe injury. Renal function is evaluated through serum creatinine (CRE) and urea nitrogen (BUN) levels, both of which increase with declining kidney function. In addition, neutrophil gelatinase‐associated lipocalin (NGAL) is a sensitive biomarker for early kidney injury, providing important information about renal damage. We first optimized the dosage of HAND for the treatment of AKI. As shown in renal function indicators detection results (Figure S23, Supporting Information), HAND could increase the therapeutic effects of AKI in a dose‐dependent manner, with the optimal efficacy observed at a dose of 2 mg kg^−1^. We then compared the effects of HAND, CND, and the clinical antioxidant NAC on the treatment of AKI were compared at the same dosage. Renal cortex morphology was observed by HE staining and the degree of renal tubular injury was scored accordingly. As shown in Figure 2B,C, RM induced severe renal tubular injury in the AKI group, characterized by obvious dilation and deformation of the renal tubules, loose arrangement between tubules, deposition of substances in the tubular lumen (casts), loss of the brush border of PTECs, PTECs swelling, necrosis, and sloughing, with a renal injury score as high as 4.5. Treatment with 2 mg kg^−1^ HANDs effectively alleviated the pathological damage of the kidneys, restoring tubule morphology and structure to near‐normal conditions. In contrast, the pathological damage remained severe in the CND group (lacking PTECs targeting) and the NAC group (Figure 2B,C), with renal injury scores of 3.2 and 3.8, respectively, which was primarily due to their lower targeting and accumulation at PTECs. Moreover, the serum CRE (Figure 2D) and BUN (Figure 2E) levels in the AKI group rose sharply, reaching 4.67 and 3.59 times those of the Control group, meeting the criteria for stage 3 AKI. Similarly, NGAL levels in serum and urine also surged (Figure 2F; Figure S24, Supporting Information), confirming the severity of renal injury. HAND treatment provided superior renal protection compared to the CND and NAC groups, with CRE, BUN, and NGAL levels returning to normal. During the experimental period, mice in the AKI group exhibited significant weight loss, whereas those in the HAND group displayed normal growth patterns (Figure S25, Supporting Information). HO‐1 and KIM‐1, specific biomarkers of renal injury, are minimally expressed in normal renal tissues but are markedly upregulated in PTECs during renal impairment. Compared with the AKI group, levels of HO‐1 (Figure 2G) and KIM‐1 (Figure 2H) in the HAND group returned to normal, demonstrating far superior therapeutic effects compared to the CND group, while the NAC group showed negligible therapeutic benefit.

The pharmacological basis of HAND is its ability to inhibit oxidative stress. To investigate this, we conducted a thorough examination of HAND's in vivo antioxidant activity as a meticulously designed reductive scavenger. Dihydroethidium (DHE) probe staining (Figure 2I; Figure S26, Supporting Information) revealed that the ROS level in the renal tissues of AKI mice was significantly elevated compared to the Control group. After treatment with HAND, the level of ROS markedly decreased to baseline, demonstrating its effectiveness in alleviating oxidative stress in the kidneys during AKI. In comparison, both CND and NAC exhibited weaker antioxidant activity at the same dosage, primarily due to their limited capacity for active targeting of PTECs. As highly destructive molecules, ROS inflict extensive cellular oxidative damage by aggressively attacking biomacromolecules, particularly DNA and membrane lipids in mitochondria and the nucleus.^[^ ^31^ ^]^ As shown in the IHC results of the DNA oxidation marker, 8‐OHdG, DNA was severely damaged and widely diffused throughout the PTECs, affecting both the nucleus and mitochondria in the AKI group (Figure 2J). Elisa's results further corroborated a significantly elevated level of 8‐OHdG in the kidney tissues of the AKI group (Figure S27, Supporting Information). Thanks to the precise targeting of mitochondria and the nucleus, HAND offered comprehensive protection for the DNA by significantly reducing DNA oxidative damage, as evidenced by the decreased levels of 8‐OHdG in both IHC and Elisa results. In comparison, CND and NAC exhibited lower DNA protection at the same dosage. Moreover, the γ‐H2AX staining results provided additional confirmation of HAND's efficacy in mitigating DNA damage. In the AKI group, a marked increase in γ‐H2AX foci was observed in PTECs, indicating widespread DNA double‐strand breaks (DSBs) (Figure S28, Supporting Information). However, treatment with HAND significantly reduced the number of γ‐H2AX‐positive cells. In contrast, CND and NAC treatments resulted in higher γ‐H2AX levels, underscoring their comparatively weaker protection against DSBs. These results collectively validate the superior capability of HAND in safeguarding DNA integrity under oxidative stress conditions in AKI. Moreover, the antioxidant effects of HAND are further underscored by the reduction in damage to lipids. As shown in Figure S29 (Supporting Information), levels of the lipid peroxidation product products malondialdehyde (MDA) and thiobarbituric acid reactive substances (TBARS) in the kidneys of the AKI group were significantly elevated, reaching two to three times those of the Control group. In the HAND group, these levels returned to normal, indicating that the heightened oxidative stress in kidney tissues was effectively mitigated by HAND. However, the levels of lipid peroxidation products in the NAC group showed no significant reduction, and the antioxidant effect of CND was notably weaker compared to the HAND group. To sum up, owing to its high‐precision hierarchical targeting and outstanding antioxidant properties, HAND demonstrated superior therapeutic benefits over CND and NAC in RM‐AKI mice, which are underscored by its effective suppression of severe oxidative stress in AKI kidneys.

### 2.4 RNA Sequencing Excavated the Therapeutic Mechanism of HAND

**Figure 3:**
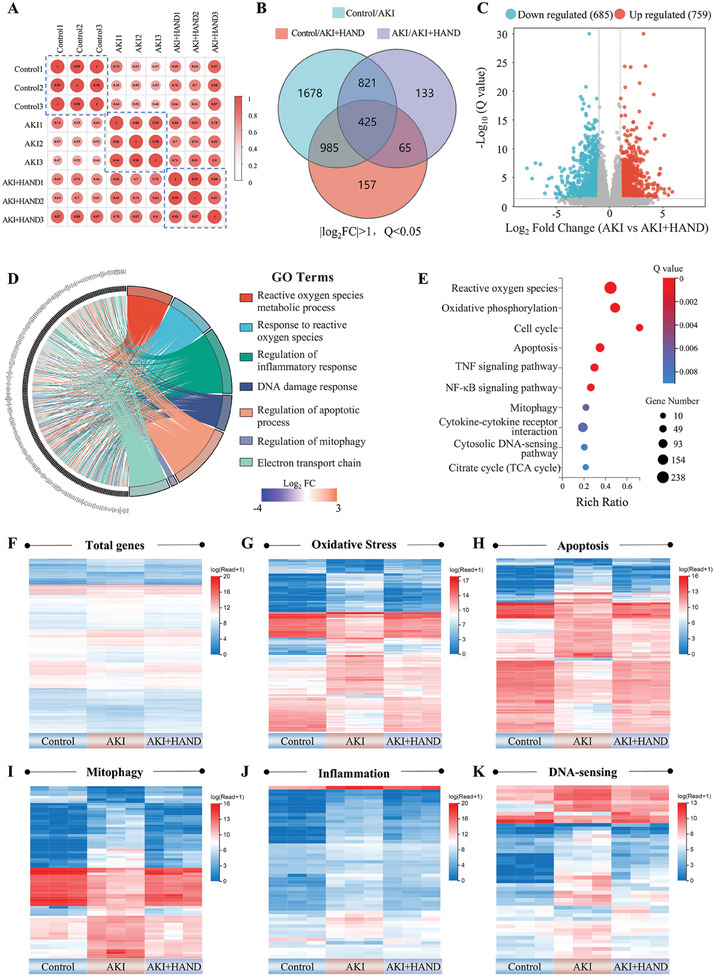
RNA‐seq revealed the therapeutic mechanisms of HAND. A) Heatmap showing the correlation of gene expression from different groups in RNA‐seq of mouse kidney tissues. B) Venn diagram of DEGs in RNA‐seq of kidney tissues. C) Volcano plot of DEGs between the AKI group and the AKI+HAND group. D,E) GO enrichment chord diagram (D) and KEGG pathway enrichment bubble chart (E) for DEGs between the AKI and AKI+HAND groups. F–K) Cluster heatmap of gene expression across different groups (F) and for five functional categories: oxidative stress (G), apoptosis (H), mitophagy (I), inflammation (J), and DNA sensing (K). *n* = 3, |log_2_FC| > 1, Q < 0.05.

To further explore the pathological mechanisms of AKI and the pharmacological effects of HAND, we conducted RNA sequencing analysis (RNA‐Seq) on kidney samples from Control, AKI, and HAND groups. The quality and reliability of the data were evaluated through correlation analysis (**Figure** **3**A) and principal component analysis (Figure S30, Supporting Information). These analyses revealed complete separation among samples from different groups, indicating significant intergroup differences and high intragroup correlation. Subsequently, we screened for differentially expressed genes (DEGs) using the criteria of |log_2_FC| > 1 and Q < 0.05. The Venn diagram (Figure 3B) and volcano plot (Figure 3C) revealed 1246 overlapping DEGs between the AKI group and the other two groups (Control group and HAND group). These DEGs underwent enrichment and clustering analyses, leveraging the Gene Ontology (GO) and Kyoto Encyclopedia of Genes and Genomes (KEGG) databases to uncover gene functions and pathways pertinent to our study. The GO enrichment analysis indicated that the DEGs between the AKI and HAND groups were primarily associated with the regulation of cellular processes such as ROS metabolism, ROS response, inflammation response, DNA damage response, apoptosis process, mitophagy, and mitochondrial ETC activity (Figure 3D). KEGG pathway enrichment analysis further revealed that these genes were involved in the regulation of pathways related to ROS, oxidative phosphorylation, cell cycle, apoptosis, TNF signaling, NF‐κB signaling, mitophagy, cytokine‐cytokine receptor interactions, cytosolic DNA sensing, and the citric acid cycle (Figure 3E). These findings underscored the therapeutic potential of HAND in alleviating oxidative stress, protecting mitochondria and DNA, and suppressing excessive inflammation during AKI, which benefited from its precise targeting capabilities and broad‐spectrum antioxidant activity. We further illustrated gene expression variations across samples through visual clustering heatmaps. As shown in Figure 3F, the overall gene expression pattern of the HAND group closely resembled that of the Control group, whereas the AKI group showed significant divergence from the other two, suggesting that HAND effectively reversed the gene expression profile alterations induced by AKI. Additionally, we constructed clustering heatmaps for five key categories—oxidative stress, apoptosis, mitophagy, inflammation, and DNA sensing—highlighted from the above enrichment analysis. As depicted in Figure 3G–K, the gene expression patterns in the HAND group more closely aligned with those in the Control group, further confirming the regulatory efficacy of HAND on these critical gene categories, which will be further explored in detail in the following sections.

### 2.5 HAND Comprehensively Protected Mitochondria

Mitochondria, as prime examples of endosymbiotic integration, coordinate critical cellular and organelle signaling pathways essential for adaptive responses and evolutionary processes, making them pivotal signaling hubs that determine cell fate.^[^ ^32^, ^33^ ^]^ During AKI, electron leakage from the mitochondrial ETC turns mitochondria into a primary source of ROS. Simultaneously, these mitochondria become the main victims of the resulting toxic ROS, which leads to severe mitochondrial dysfunction, disrupted energy metabolism, and heightened ROS production, ultimately triggering a ROS storm that engulfs the entire PTECs.^[^ ^34^, ^35^ ^]^ Consequently, mitochondrial health emerges as a crucial target in AKI therapy, serving as a direct indicator of the effectiveness of therapeutic interventions, a theme we have examined comprehensively in this section.

**Figure 4:**
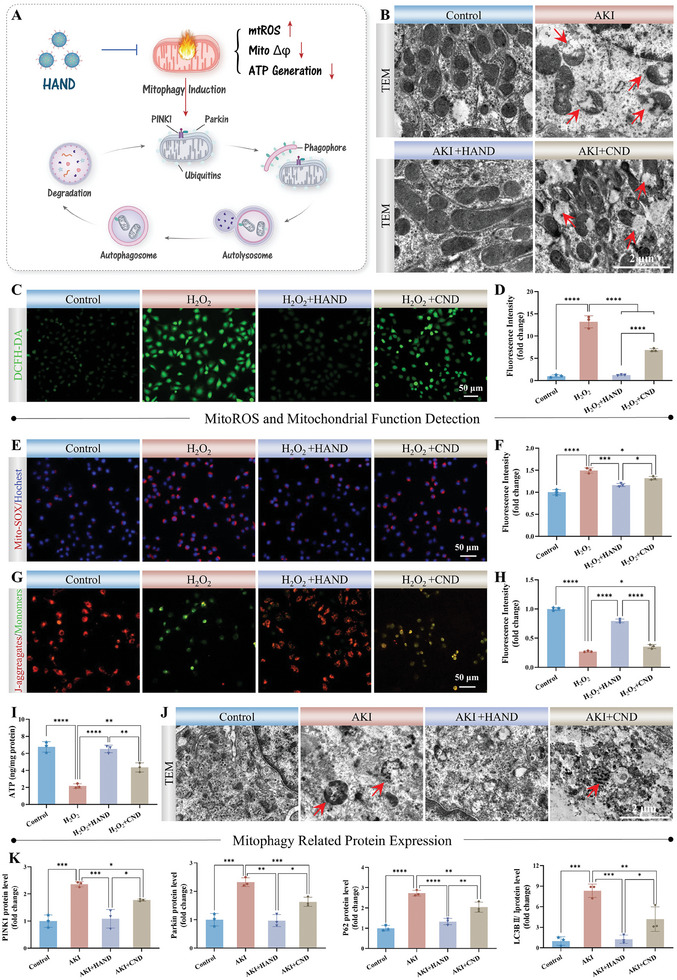
Comprehensive mitochondrial protection of HAND. A) Schematic illustration of the mitochondrial protecting ability of HAND. B) TEM images of mitochondria in PTECs from different treatment groups of mice. C,D) Representative DCFH‐DA fluorescence images (C) and corresponding fluorescence intensity (D) of HK‐2 cells in different treatment groups. E,F) Mito‐SOX fluorescence staining images and corresponding fluorescence intensity (F) of HK‐2 cells from different treatment groups. G,H) JC‐1 fluorescence staining images (G) and corresponding fluorescence intensity (H) of HK‐2 cells from different treatment groups. I) ATP production of HK‐2 cells from different treatment groups. J) TEM images of mitophagy in PTECs from different treatment groups of mice. K) The grayscale analysis of PINK1, Parkin, P62, and LC3BII/I of Western blot results from kidney tissues in different treatment groups. Data are presented as mean ± SD. One‐way ANOVA followed by the SNK test was used for analysis. *n* = 3, ^*^ *p* < 0.05, ^**^ *p* < 0.01, ^***^ *p* < 0.001, ^****^ *p* < 0.0001.

HAND exhibited precise mitochondrial targeting and sustained antioxidant activity, enabling it to effectively eliminate mtROS in situ and protect mitochondria from oxidative damage and its detrimental consequences (**Figure** **4**A). We conducted a series of experiments to validate the comprehensive protective effects of HAND on mitochondrial structure, function, oxidative stress status, and homeostatic regulation. First, we analyzed the mitochondrial morphology of PTECs across different groups using TEM. In contrast to the intact mitochondria observed in the control group, those in the AKI group exhibited significant structural damage, including swelling, rupture, and cristae fragmentation (Figure 4B). Following HAND treatment, mitochondrial morphology in the AKI mice showed notable restoration, while the CND group continued to display morphological abnormalities. Next, we investigated the mitochondrial protective mechanisms of HAND in HK‐2 cells. DCFH‐DA (ROS probe) detection results showed that HAND significantly alleviated the overall increase in intracellular ROS levels induced by H_2_O_2_ (Figure 4C,D). Additionally, mitoSOX (mtROS‐specific probe with red fluorescence) fluorescence imaging and flow cytometry analysis further revealed the potent mtROS clearance of HAND under H_2_O_2_ stimulation, underscoring its targeted mitochondrial protection (Figure 4E,F; Figure S31, Supporting Information). Furthermore, mitochondria maintain an electrochemical gradient across their inner membrane via proton pumps in the ETC, which drives ATP synthesis. Thus, mitochondrial membrane potential (MMP) and ATP levels are key indicators of mitochondrial function. We evaluated the effect of HAND on MMP under H_2_O_2_ stimulation using the JC‐1 fluorescent dye. As shown in Figure 4G,H; Figure S32 (Supporting Information), H_2_O_2_ administration led to a significant decrease in MMP, which was indicated by the predominant green fluorescence corresponding to JC‐1 monomers in the mitochondria. Co‐incubation with HAND effectively mitigated the loss of MMP, resulting in the formation of JC‐1 aggregates that emitted red fluorescence in mitochondria. Consequently, the restoration of MMP ensured the efficiency and integrity of the mitochondrial ETC, leading to a notable recovery of ATP production in the HAND group compared to the H_2_O_2_ group (Figure 4I). Moreover, mitophagy is another indicator of mitochondrial damage, aiding in the removal of impaired mitochondria to prevent further damage.^[^ ^36^ ^]^ Mitophagy is tightly regulated by various signaling pathways and proteins, with the PINK1‐Parkin pathway being the most important.^[^ ^37^ ^]^ PINK1, which contains a mitochondrial targeting sequence at its N‐terminus, is normally located in mitochondria, where it is cleaved and degraded by the proteasome. However, disruptions such as MMP loss disrupt this process, leading to PINK1 accumulation on the outer mitochondrial membrane. Activated PINK1 recruits Parkin from the cytosol to the mitochondria, where Parkin, an E3 ubiquitin ligase, ubiquitinates outer mitochondrial membrane proteins, attracting autophagic vesicle proteins like LC3B II. During this process, P62/SQSTM1 acts as a bridging protein, linking LC3B II with ubiquitinated proteins on the mitochondrial surface, promoting the formation of autophagosome1^[^ ^12^ ^]^ (Figure 4A). TEM images showed that during AKI, high levels of mtROS caused severe mitochondrial damage and activated mitophagy, resulting in the presence of many autophagosomes in PTECs (Figure 4J). Western blot (WB) analysis further revealed that the expression levels of mitophagy‐related proteins, including PINK1, Parkin, P62, and LC3BII/I, were significantly elevated in AKI kidneys, indicating high mitophagy activation. HAND treatment reduced the number of autophagosomes in PTECs and downregulated the expression of mitophagy‐related proteins compared to the AKI group, suggesting reduced mitochondrial damage (Figure 4K; Figure S33, Supporting Information). In summary, HAND provided exceptional, comprehensive protection for mitochondria due to its high‐resolution mitochondrial localization and remarkable antioxidant capacity, thereby preserving the most critical organelles in PTECs during AKI.

### 2.6 HAND Effectively Inhibited Apoptosis of PTECs

**Figure 5:**
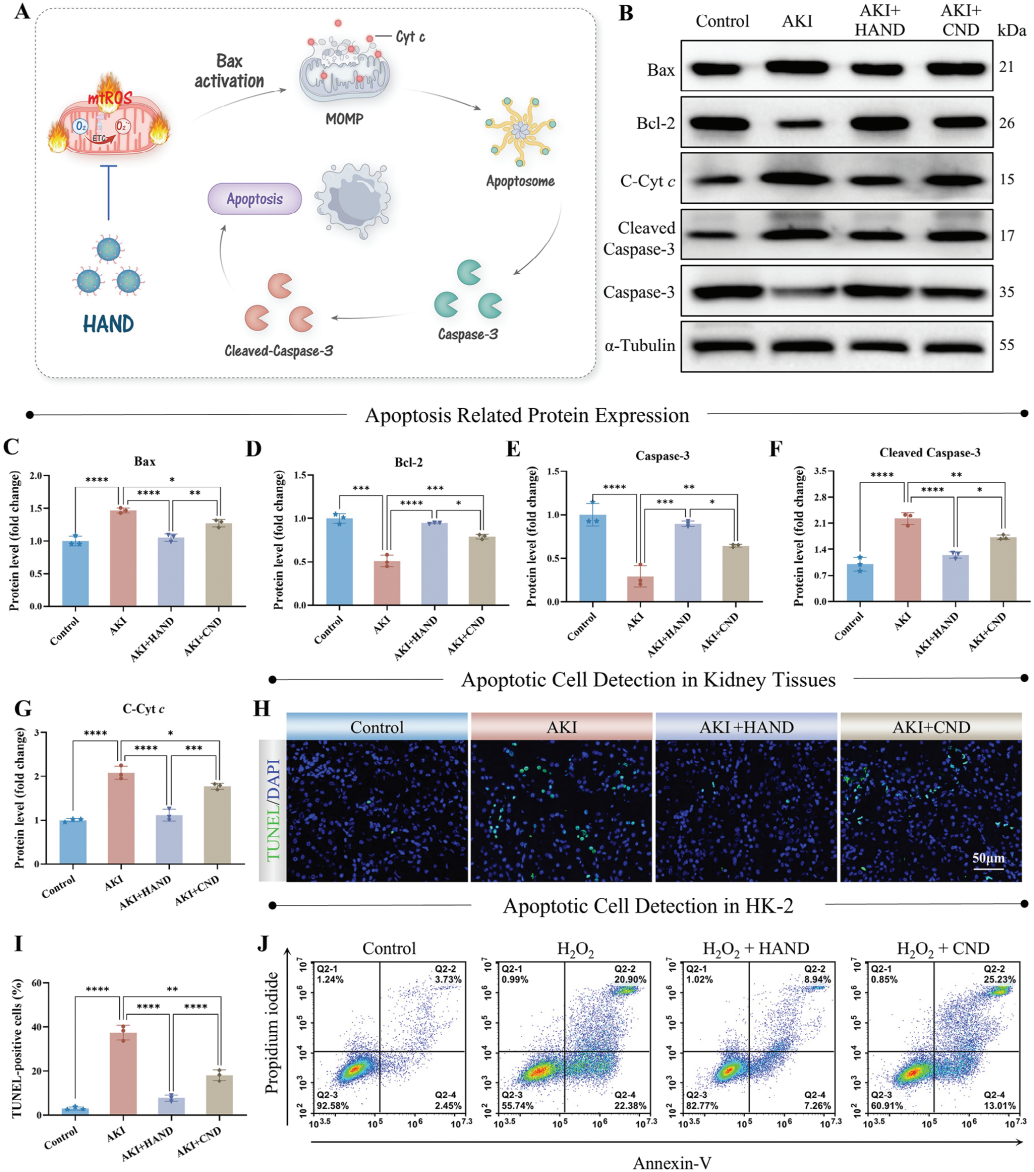
HAND effectively inhibited intrinsic apoptosis. A) Schematic illustration of HAND inhibiting intrinsic apoptosis. B–G) Western blot analysis of apoptosis‐related protein expression levels in mouse kidney tissues from different treatment groups and grayscale analysis of Bax (C), Bcl‐2 (D), Caspase‐3 (E), Cleaved‐Caspase 3 (F), and C‐Cyt *c* (G). (H‐I) TUNEL fluorescence staining results (H) and quantification of TUNEL‐positive cells (I) in mouse kidneys from different treatment groups. J) Annexin‐V/PI flow cytometry results in HK‐2 cells from different treatment groups. Data are presented as mean ± SD. One‐way ANOVA followed by the SNK test was used for analysis. *n* = 3, ^*^ *p* < 0.05, ^**^ *p* < 0.01, ^***^ *p* < 0.001, ^****^ *p* < 0.0001.

As central arbiters of cellular function and integrity, mitochondria are crucial in mediating cell death signaling, particularly in intrinsic apoptosis (also known as the mitochondrial pathway of apoptosis)^[^ ^38^ ^]^ (**Figure** **5**A). In the pathological state of AKI, mtROS damages mitochondrial lipids and proteins, leading to compromised membrane integrity and inducing mitochondrial outer membrane permeabilization (MOMP). MOMP, the initiating event of intrinsic apoptosis, is primarily mediated by pro‐apoptotic members of the Bcl‐2 family, such as Bax. Under oxidative stress, Bax accumulates on the mitochondrial outer membrane, forming pores that enable the release of essential apoptotic factors, such as cytochrome *c* (Cyt *c*), into the cytosol. Once released, Cyt *c* promptly activates the caspase cascade, with Caspase‐3 serving as the principal executor. Upon activation through cleavage, it degrades various cellular components, thereby driving apoptosis.^[^ ^39^ ^]^ As shown in the WB results (Figure 5B–G), expression levels of Bax and Cytosolic‐Cyt *c* (C‐Cyt *c*) were significantly upregulated in the kidneys of the AKI group, while the expression of the anti‐apoptotic protein Bcl‐2 was downregulated, indicating the activation of intrinsic apoptosis. Furthermore, the caspase cascade was effectively triggered, with Caspase‐3 being cleaved and downregulated, which corresponded to a significant increase in Cleaved‐Caspase‐3 levels, confirming the execution of intrinsic apoptosis. Importantly, following HAND treatment, the expression of mitochondrial pro‐apoptotic proteins, including Bax, C‐Cyt *c*, and Cleaved‐Caspase‐3, was significantly reduced in the kidneys, while Bcl‐2 levels were markedly increased compared to the AKI group. Moreover, DNA fragmentation induced by Caspase‐activated DNase or ROS is a critical hallmark of apoptosis, leading to chromatin fragmentation and condensation, ultimately resulting in irreversible cell death. We then validated the anti‐apoptotic effect of HAND through TUNEL staining, which identifies and labels DNA strand breaks. As shown in Figure 5H,I, the number of TUNEL‐positive cells in the renal tubules of the AKI group was significantly increased compared to the Control group, with an apoptotic cell rate of 37%. Notably, HAND treatment significantly reduced the proportion of TUNEL‐positive cells, demonstrating superior anti‐apoptotic efficacy compared to CND without PTEC targeting. Subsequently, the anti‐apoptotic effect of HAND was further validated in HK‐2 cells through Annexin V‐FITC/PI flow cytometry. Annexin V specifically binds to phosphatidylserines, which translocate to the outer leaflet of the cell membrane during early apoptosis, while propidium iodide (PI) penetrates only late apoptotic cells with compromised membranes to bind to DNA. Under H_2_O_2_ stimulation, HK‐2 cells exhibited a high apoptosis rate, with the proportion of early and late apoptotic cells reaching 49.2%, which was significantly reduced to 14.9% following HAND treatment (Figure 5J; Figure S34, Supporting Information). Additionally, WB analysis confirmed that HAND effectively down‐regulated the expression of pro‐apoptotic proteins in HK‐2 cells induced by H_2_O_2_ (Figure S35, Supporting Information). Collectively, these findings demonstrated the superior anti‐apoptotic protective effects of HAND in both in vivo and in vitro models.

### 2.7 HAND‐Inhibited cGAS‐STING‐Mediated Sterile Inflammation

**Figure 6:**
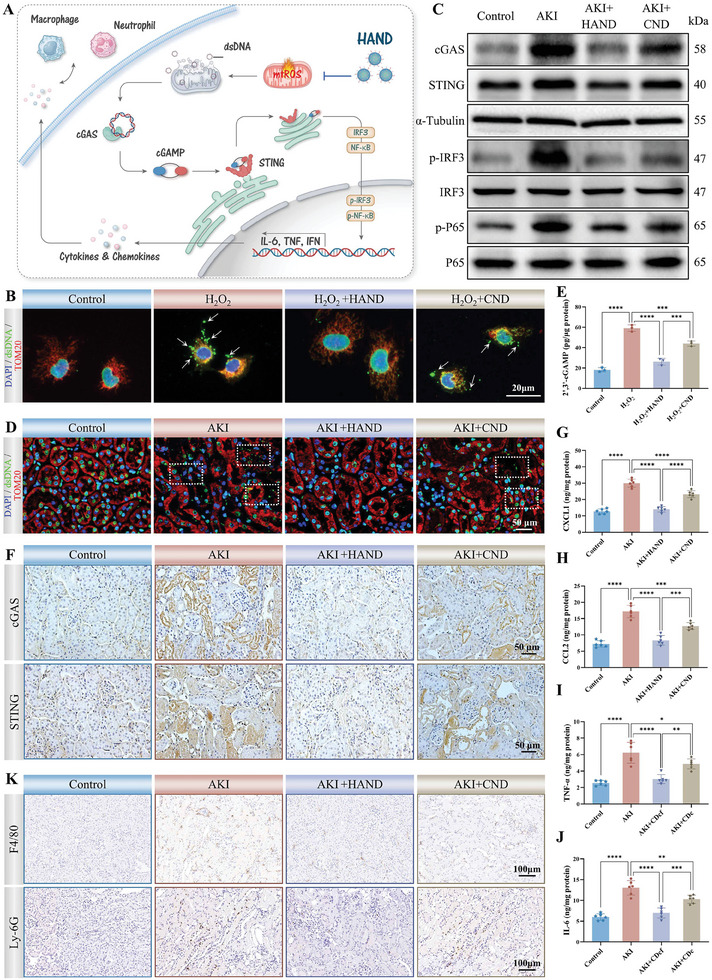
HAND inhibited cGAS‐STING mediated sterile inflammation. A) Schematic illustration of HAND inhibiting cGAS‐STING mediated sterile inflammation. B) Fluorescence staining results of dsDNA (green), TOM20 (red), and DAPI (blue) in HK‐2 cells from different treatment groups. C) WB analysis of cGAS‐STING related protein expression in HK‐2 cells. D) Fluorescence staining results of dsDNA (green), TOM20 (red), and DAPI (blue) in kidneys from different treatment groups. E) Levels of 2′‐3′cGAMP in kidneys from different treatment groups. F) IHC results of cGAS and STING in kidneys from different treatment groups. G–J) Levels of chemokines CXCL1 (G), CCL2 (H), and inflammatory cytokines TNF‐α (I), and IL‐6 (J) in kidneys from different treatment groups. K) IHC results of F4/80 and Ly‐6G in kidneys from different treatment groups. Data are presented as mean ± SD. One‐way ANOVA followed by the SNK test was used for analysis. *n* = 3, ^*^ *p* < 0.05, ^**^ *p* < 0.01, ^***^ *p* < 0.001, ^****^ *p* < 0.0001.

Excessive and unresolved sterile inflammation during AKI exacerbates tissue damage and accelerates the progression of renal fibrosis.^[^ ^40^ ^]^ Because in the absence of pathogens, the indiscriminate attacks by immune cells are mainly directed against endogenous tissues.^[^ ^41^ ^]^ Notably, mitochondria and the nucleus, beyond their central role in mediating cell death during AKI, also serve as key regulators of the inflammatory response, significantly influencing inflammatory signaling through the release of damaged DNA. The precise targeting capability of HAND to mitochondria and the nucleus, along with its potent mtROS‐scavenging efficacy, offers HAND a significant advantage in modulating inflammation during AKI (**Figure** **6**A). The activation of the cGAS‐STING DNA‐sensing pathway has been increasingly recognized as a contributor to AKI‐related sterile inflammation, initiating a cascade of biological effects by activating innate immune responses. cGAS, a cytosolic DNA sensor, detects double‐stranded DNA (dsDNA) and catalyzes the synthesis of the second messenger cGAMP. Thus, the aberrant presence of cytosolic dsDNA is amplified by cGAMP and transmitted to the ER membrane receptor STING. This interaction induces conformational changes in STING, triggering downstream signaling cascades, including the activation of IRF3 and the phosphorylation and nuclear translocation of NF‐κB.^[^ ^11^ ^]^ In eukaryotic cells, the nucleus and mitochondria are the exclusive compartments for DNA, both of which undergo intense oxidative stress during AKI, leading to the production of substantial oxidative‐DNA fragments.

As shown in Figure 6B, H_2_O_2_ stimulation significantly reduced the colocalization of dsDNA with the nucleus and mitochondria in HK‐2 cells. Further quantitative results showed that the level of mtDNA in the cytoplasm of the H_2_O_2_ group increased significantly, while the mtDNA level in mitochondria decreased, which proved that the contents were released due to membrane rupture during mitochondrial injury (Figure S36, Supporting Information). The released dsDNA then strongly sensitized the cGAS/STING pathway, evidenced by the significant upregulation of cGAS and STING in HK‐2 cells, as well as the activation of downstream IRF3 and phosphorylation of NF‐κB (Figure 6C). Notably, HAND treatment effectively mitigated dsDNA leakage and subsequent cGAS‐STING activation by mitigating DNA oxidative damage (Figure 6B,C; Figures S36,S37, Supporting Information). We further validated the modulation of HAND on cGAS‐STING‐mediated inflammatory response in AKI mice. PTECs play a central role in AKI‐induced damage, serving as the initial site of injury that propagates signals to induce an inflammatory response. As shown in Figure 6D, the colocalization of dsDNA with TOM20 and DAPI in the renal cortex was significantly reduced in the AKI group, with dsDNA dispersed into the extracellular space. cGAS was then highly sensitized, generating a large amount of second messenger to activate STING, as evidenced by the high levels of 2′‐3′ cGAMP in renal tissue (Figure 6E) and the elevated expression of cGAS and STING in damaged proximal tubules (Figure 6F). Correspondingly, downstream pro‐inflammatory signals of the cGAS/STING pathway were activated (Figure S38, Supporting Information), mediating the expression and secretion of various inflammatory cytokines (such as TNF‐α, IL‐1β, IL‐6) and chemokines (CXCL1, CXCL2, CCL2) (Figure 6G–J; Figure S39, Supporting Information). Among these, CXCL1 and CXCL2 primarily recruit neutrophils, while CCL2 mainly recruits macrophages and other monocytes. As a result, in contrast to the small number of resident immune cells in the Control group, the AKI group displayed extensive infiltration of neutrophils and macrophages in the renal tubular interstitium. These recruited immune cells further released inflammatory mediators such as cytokines, chemokines, ROS, and proteases, perpetuating a vicious cycle that fosters chronic inflammation. Such persistent and extensive damage to PTECs would ultimately deplete the functional epithelium, resulting in the formation of scar tissue formation and irreversible fibrosis. Fortunately, the potent ROS‐scavenging ability of HAND effectively disrupted the vicious cycle of oxidative stress, inflammation, and tissue damage, as evidenced by the significantly reduced levels of inflammatory mediators and decreased immune cell infiltration in the HAND group, demonstrating superior immunomodulatory effects compared to CND (Figure 6K). Furthermore, to substantiate these findings, we employed a conditioned medium model to investigate the interactions between injured PTECs and immune cells under oxidative stress. HK‐2 cells were first treated with H_2_O_2_ to induce oxidative damage and activation of the cGAS‐STING pathway, after which the conditioned medium from these HK‐2 cells was collected and used to stimulate RAW 264.7 macrophages (Figure S40 (A), Supporting Information). In this system, macrophages exhibited significant activation, as evidenced by the upregulation of COX‐2 and iNOS, along with elevated levels of pro‐inflammatory cytokines (TNF‐α, IL‐1β, IL‐6), which further exacerbated inflammation. However, HAND treatment to H_2_O_2_‐stimulated HK‐2 cells resulted in a marked reduction in COX‐2 and iNOS expression in RAW 264.7 macrophages, as well as significantly lower levels of pro‐inflammatory cytokines compared to the CND group (Figure S40 (B), Supporting Information). The enhanced anti‐inflammatory effect is primarily attributed to HAND's ability to protect HK‐2 cells from oxidative damage, underscoring the therapeutic advantage of HAND in mitigating oxidative stress‐induced inflammation by preserving cellular integrity and minimizing damage‐associated signals that drive excessive immune responses. Taken together, the above findings suggested that HAND could successfully halt the pathological progression of AKI by precisely eliminating the source of ROS and inhibiting the cGAS‐STING‐mediated sterile inflammation.

### 2.8 HAND Exhibited Excellent Biocompatibility

**Figure 7:**
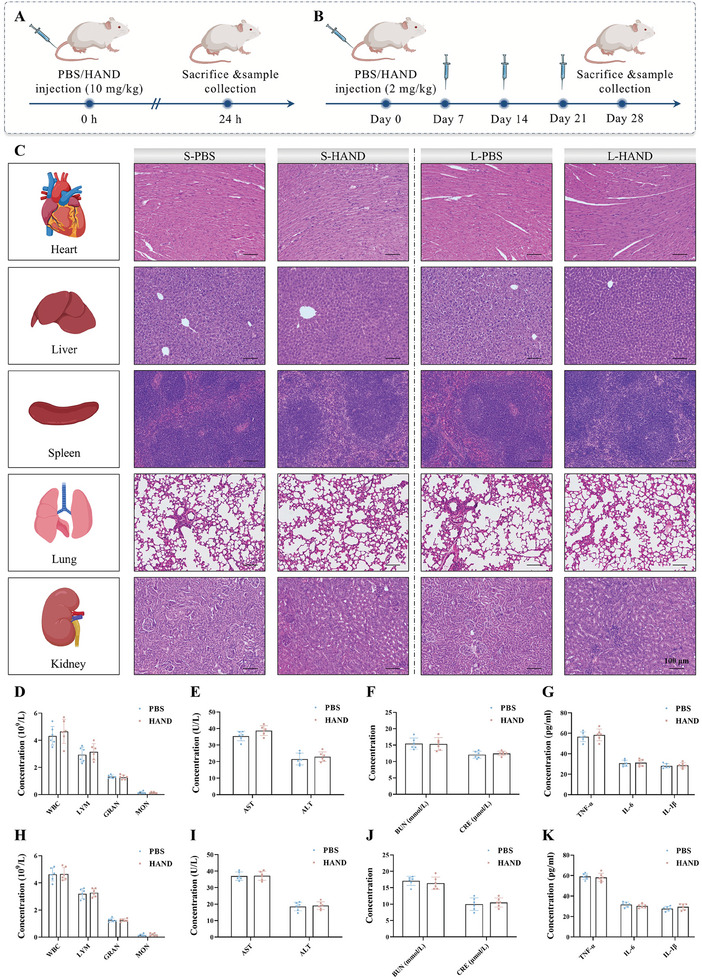
HAND exhibited excellent biocompatibility. A,B) Schematic illustration of short‐term and long‐term treatment protocol for biosafety evaluation of HAND. C) Representative H&E‐stained images of major organs from KM mice after short‐term (left) and long‐term (right) administration of HAND or 1× PBS. D–G) Hematological parameters (D), liver function indicators (E), kidney function indicators (F), and inflammatory cytokine levels (G) of short‐term treated mice in different treatment groups. H–K) Hematological parameters (H), liver function indicators (I), kidney function indicators (J), and inflammatory cytokine levels (K) of long‐term treated mice in different treatment groups. Data are presented as mean ± SD. *n* = 6.

Finally, we evaluated the biocompatibility of HAND in vivo (**Figure** **7**A,B). Following the administration of a high dosage (10 mg kg^−1^, 5 times the therapeutic dose) for 24 h, the lungs, liver, heart, spleen, and kidneys were harvested for HE staining. Compared with the PBS group, HAND did not cause any damage to these vital organs (Figure 7C). Additionally, hematological parameters, including white blood cells, red blood cells, and platelets, showed no significant differences compared to the PBS group (Figure 7D; Figure S41, Supporting Information). Liver function indicators (AST, ALT) (Figure 7E), kidney function markers (BUN, CRE) (Figure 7F), and inflammatory cytokine levels (TNF‐α, IL‐6, IL‐1β) (Figure 7G) also remained stable, indicating that HAND did not adversely affect liver or kidney function and did not provoke an inflammatory response. Moreover, during long‐term administration of HAND in mice (therapeutic dosage once a week for 4 weeks), no significant changes were observed in major organ histology (Figure 7C), hematological parameters (Figure 7H; Figure S42, Supporting Information), liver and kidney function (Figure 7I,J), or inflammatory markers (Figure 7K). These results collectively demonstrated the excellent biosafety of the naturally derived HAND.

## 3 Discussion

Over the past two decades, the proportion of deaths attributed to kidney disease has steadily increased, with its global prevalence surpassing that of any other major non‐communicable diseases, including cardiovascular diseases, cancer, chronic respiratory diseases, and diabetes.^[^ ^42^ ^]^ Patients with kidney disease are at high risk for comorbidities that contribute not only to elevated mortality rates but also to a significant symptom burden and substantial treatment costs.^[^ ^43^ ^]^ Given the urgent clinical demand, the development of therapeutics for kidney diseases holds significant promise but also faces substantial challenges. Recent advancements in genetics, bioinformatics, and pharmacodynamics have facilitated deeper exploration into g the pathophysiology of AKI and the development of new drugs. However, the diverse etiologies and heterogeneous pathological processes of AKI complicate efforts to create effective treatments targeting gene regulation or specific downstream molecules, which may fail to rapidly halt acute damage progression. Because the oxidative stress‐PTECs injury‐inflammation vicious cycle is a key factor in almost all types of AKI, which interferes with numerous cellular processes and spreads rapidly like a snowball to cause a swift decline in renal function.^[^ ^16^ ^]^ This cycle begins with the excessive production of ROS in response to various stressors, leading to oxidative stress that directly damages PTECs. Once injured, PTECs release pro‐inflammatory cytokines that intensify the inflammatory response and attract immune cells to the injury site. The influx of these inflammatory cells, in turn, generates additional ROS, creating a feedback loop that perpetuates cellular damage and inflammation. As the cycle continues, impairs vital cellular functions, including mitochondrial activity and signaling pathways, ultimately resulting in rapid renal function decline. Moreover, the increasing emphasis on precision medicine underscores the need for more organized, rational, and high‐resolution targeting strategies.^[^ ^44^ ^]^ Non‐selective drug distribution often necessitates higher therapeutic doses and leads to unavoidable off‐target toxic side effects.^[^ ^45^ ^]^ These challenges significantly complicate the development of AKI therapies, highlighting the urgent need for more precise and efficient treatment strategies.

In this context, we specifically target the primary source of injury in AKI—excessive toxic ROS—and designed an FA‐optimized nanomedicine, HAND, that aligns with molecular preferences for targeted tissues (kidneys), cells (PTECs), and organelles (mitochondria and nucleus). Driven by glomerular hydrostatic pressure, HAND, with its ultrasmall size, efficiently traversed the GFB to reach the renal tubules. Subsequently, the densely packed brush border of PTECs, extending into the lumen, captured the filtered HAND through contractile movements, and effectively absorbed it via FR‐mediated endocytosis. The active drug uptake mechanism prevented rapid passive clearance through renal excretion. Notably, the compartmentalized utilization of FA within cells may drive the selective intracellular distribution of HAND, leading to its significant colocalization with the mitochondria and nucleus. Simultaneously, FA‐derived groups of HAND mediated its strong adsorption to these two organelles. Consequently, HAND achieved precise accumulation in mitochondria and nucleus, where it scavenged destructive ROS in situ, thereby mitigating a range of downstream oxidative stress‐related consequences such as mitochondrial stress, DNA damage, cell death, and cGAS‐STING‐mediated sterile inflammation.

While HAND holds significant potential, there are still a few concerns that need to be carefully addressed for its successful clinical translation. First, like many nanomedicines, the potential for immune responses or unanticipated interactions with other biological systems in humans warrants further investigation. Second, although preclinical models demonstrate highly selective targeting, translating this precision to humans—where individual variability can affect drug distribution—necessitates additional exploration. Moreover, the interaction between FA and key proteins in the mitochondria and nucleus is critical to HAND's targeting mechanism, yet the specific pathways remain unclear. The intracellular metabolism and transport dynamics of FA also merit further investigation, as these factors could significantly influence the in vivo behavior of FA‐based nanodrugs. This complexity poses a long‐term challenge that demands collaborative efforts across multiple disciplines to fully understand and address. Future studies focusing on FA transport dynamics and its interactions with mitochondrial and nuclear components will provide essential insights for optimizing the design of FA‐based nanodrugs for clinical use. Lastly, extensive studies are required to validate HAND's therapeutic benefits beyond the acute phase, ensuring long‐term safety and efficacy. However, overcoming these challenges is feasible and will yield valuable insights for refining HAND's design for broader applications. Notably, HAND's high efficacy and precision in targeting multiple key injury sites simultaneously represent a significant advancement in AKI therapy. This approach enhances therapeutic outcomes while substantially reducing dosage and potential off‐target effects, making HAND a promising candidate for future clinical applications. Although further studies are necessary to fully elucidate HAND's long‐term effects and scalability, its current success in preclinical models provides a strong foundation for optimism.

Overall, this work integrated hierarchical, high‐precision targeting with broad‐spectrum antioxidant activity—typically requiring complex construction—into a simple and elegant nanomedicine, thereby significantly lowering the therapeutic dosage and minimizing side effects. We hope that this strategy, which focuses on eliminating common damage factors and targeting key injury sites, will pave the way for innovative therapeutic approaches and provide valuable guidance in treating AKI and other kidney diseases.

## 4 Conclusion

In this study, we present a hierarchical targeting strategy for AKI antioxidant therapy with functional nanomedicine‐HAND. Derived from natural antioxidants and essential nutrients, HAND demonstrated excellent antioxidant activity and biocompatibility. The functionalization with FA endowed HAND with multi‐dimensional targeting capabilities, allowing for the selective elimination of ROS in the mitochondria and nucleus of PTECs. Consequently, HAND significantly ameliorated oxidative stress and excessive inflammation in the renal proximal tubules during AKI, restoring kidney function and halting the progression of AKI at an extremely low dose. Overall, the tailored HAND carefully integrated and harmonized multiple considerations, including subcellular precision targeting, broad‐spectrum antioxidant capacity, and exceptional biosafety, offering promising new insights and valuable information for the development of tailored therapies for AKI.

## 5 Experimental Section

### Materials

The used reagents are shown in **Table** **1**.

### Synthesis of HAND and CND

Accurately weigh 0.250 g of curcumin powder and 0.500 g of folic acid powder. Precisely measure 20.0 mL of ultrapure water. Dissolve the weighed powders in the ultrapure water, ensuring complete dissolution and uniform dispersion, and then react at 240 °C for 6 h. After the reaction, collect the supernatant and purify it by dialysis to obtain the HAND solution. The preparation process for CND is the same as for HAND, except that only 0.750 g of curcumin powder was used as the carbon source.

**Table 1:** List of reagents.

Reagents	Source	Catalog
Folic acid	Macklin	F809516
Curcumin	Macklin	C805204
Fluorescein isothiocyanate	Macklin	F6120
Methionine	Macklin	L812760
Riboflavin	Macklin	R817215
Nitro‐blue tetrazolium	Macklin	N814596
Glycerol	Macklin	G810575
Hydrogen peroxide	Sigma	216763
Ferrous sulfate	Aladdin	F116338
Dihydroethidium	Sigma	309800
Mitochondrial membrane potential assay kit with JC‐1	Beyotime	C2006
MitoSOX Red Mitochondrial Superoxide Indicator	Yeasen	40778ES50
DCFH‐DA	Sigma	35845
Malondialdehyde assay kit	Jiancheng Bioengineering Institute	A003‐1
TUNEL assay kit	Beyotime	C1098
DAB HRP kit	Beyotime	P0203
Enhanced BCA Protein Assay Kit	Beyotime	P0010
Hoechst 33342	Beyotime	C1022
Antifade Mounting Medium with DAPI	Beyotime	P0131
Enhanced ATP Assay Kit	Beyotime	S0027
Annexin V‐FITC/PI Apoptosis Detection Kit	Yeasen	40302ES50
ER‐tracker Red	Beyotime	C1041
Mito‐tracker Red	Beyotime	C1035
Golgi‐tracker Red	Beyotime	C1043
Lyso‐tracker Red	Beyotime	C1046
CRE assay kit	Jiancheng Bioengineering Institute	C011‐2‐1
TBARS assay kit	Elabscience	E‐BC‐K298‐M
BUN assay kit	Jiancheng Bioengineering Institute	C013‐2‐1
GSH/GSSG assay kit	Beyotime	S0053
CAT assay kit	Jiancheng Bioengineering Institute	A007‐1‐1
SOD assay kit	Jiancheng Bioengineering Institute	A001‐3‐2
Mitochondrial separation and protein extraction kit	Proteintech	PK10016
MolPure® Cell/Tissue DNA Kit	Yeasen	18700ES50
dsDNA HS Assay Kit	Yeasen	12640ES60

### Synthesis of FITC‐HAND and FITC‐CND

Dissolve 10 mg of HAND or CND in 8 mL of ddH_2_O. For HAND, add 2 mg of FITC (dissolved in DMSO, 1 mg mL^−1^), and stir to react for 8 h at room temperature in the dark. The isothiocyanate group of FITC underwent a covalent conjugation reaction with the amino groups present in HAND, resulting in the formation of stable FITC‐HAND conjugates. For CND, add 2 mg of FITC (dissolved in DMSO, 1 mg mL^−1^) to the CND solution, adjust the pH to 9–10 by adding NaOH to create an alkaline environment, and stir the mixture to react for 8 h at room temperature in the dark. In this basic condition, the hydroxyl groups on CND were deprotonated and reacted with FITC, forming a covalent bond between the hydroxyl groups and the isothiocyanate group of FITC, resulting in FITC‐CND conjugates. After the reaction, dialyze the solution for 1–2 days to remove unreacted impurities and obtain FITC‐HAND or FITC‐CND.

### Synthesis of Cy7‐HAND

Briefly, HAND (10 mg) was dispersed in PBS (pH 8.5) and mixed with Cy7‐NHS (2 mg) under gentle stirring. The reaction was allowed to proceed for 4 h at room temperature in the dark to prevent photobleaching of Cy7. Following conjugation, the mixture was dialyzed against PBS for 24 h to remove unreacted Cy7 dye and byproducts. The purified Cy7‐HAND was then collected.

### Characterization of the Morphology, Structure, and Spectra of HAND, CND, FITC‐HAND, and FITC‐CND

Images of HAND and CND were captured using a TECNAI G2 high‐resolution Transmission Electron Microscope (TEM). X‐ray Photoelectron Spectroscopy (XPS) of nanodrugs was conducted using a VG ESCALAB MKII spectrometer. The infrared spectra of curcumin, folic acid, HAND, and CND were obtained using a Bruker Vertex 7 Fourier Transform Infrared Spectrometer. The UV–vis spectra of nanodrugs were scanned with a UV‐3600i Plus UV–vis Spectrophotometer. The surface potentials of HAND and CND were measured using a Zeta potential analyzer.

### In Vitro SOD‐Mimicking Activity Assay of HAND and CND

The O_2_^·−^the scavenging ability of HAND and CND was assessed using the Nitro Blue Tetrazolium (NBT) method to calculate their in vitro SOD‐mimicking activity. The reaction system comprised methionine (0.1 m), riboflavin (20 µm), NBT (0.01 m), PBS (0.1 m, pH 7.4), and the HAND/CND (1 mg mL^−1^). In the presence of methionine, riboflavin underwent photochemical reduction upon UV light exposure and was subsequently oxidized in an oxygenated environment to produce O_2_^·−^. O_2_^·−^ could reduce NBT to blue formazan, which exhibited a characteristic absorption peak at 560 nm. The HAND/CND scavenge O_2_^·−^ in a concentration‐dependent manner, resulting in decreased UV absorption, showing a dose‐response trend. The O_2_^·−^the scavenging ability of HAND/CND was calculated based on the inhibition of NBT photochemical reduction by HAND and CND.

### In Vitro ·OH Scavenging Ability Assay of HAND and CND

The in vitro ·OH scavenging ability of HAND and CND was evaluated using the 3,3′,5,5′‐Tetramethyl Benzidine Dihydrochloride (TMB) method. The reaction system consisted of FeSO_4_ (5 mm), TMB (5 mm), H_2_O_2_ (100 mm), PBS (0.01 m, pH 7.4), and the HAND/CND (1 mg mL^−1^). The Fenton reaction between FeSO_4_ and H_2_O_2_ catalyzed the generation of ·OH. Highly reactive ·OH oxidizes TMB, causing a color change from colorless to blue, with the blue diamine product exhibiting a maximum characteristic UV absorption peak at 652 nm. HAND/CND scavenges ·OH in a concentration‐dependent manner, resulting in decreased UV absorption, showing a dose‐response trend.

### In Vitro ONOO^−^ Scavenging Ability Assay of HAND and CND

The in vitro ONOO^−^ scavenging ability of HAND and CND was measured using a fluorescence method. The reaction system consisted of varying concentrations of ONOO^−^ and HAND/CND (0.1 mg mL^−1^). ONOO^−^ reacted with the drugs in a concentration‐dependent manner, leading to a decrease in the fluorescence emission intensity of the drugs, showing a dose‐response trend.

### In Vitro H_2_O_2_ Scavenging Ability Assay of HAND and CND

The in vitro H_2_O_2_ scavenging ability of HAND and CND was measured using a fluorescence method. The reaction system consisted of varying concentrations of H_2_O_2_ and HAND/CND (0.1 mg mL^−1^). H_2_O_2_ reacts with the drugs in a concentration‐dependent manner, resulting in a decrease in the fluorescence emission intensity of the drugs, showing a dose‐response trend.

### Cell Culture

HK‐2 cells were cultured in DMEM/F‐12 medium supplemented with 10% fetal bovine serum and incubated in a 5% CO_2_, 37 °C incubator. H9c2 cells were cultured in high‐glucose DMEM supplemented with 10% fetal bovine serum (FBS) and incubated in a 5% CO_2_, 37 °C incubator. LX‐2 cells RAW 264.7 cells were cultured in DMEM supplemented with 10% FBS and 1% penicillin‐streptomycin, and maintained in a 5% CO_2_, 37 °C incubator.

### Conditioned Medium Model

HK‐2 cells were stimulated with H_2_O_2_ and treated with HAND. Following the treatment, the conditioned medium (supernatant) from the HK‐2 cells was collected and used to stimulate RAW 264.7 macrophages. The supernatant from RAW 264.7 macrophages was collected and used to pro‐the detection of inflammatory cytokines (IL‐6, IL‐1β, TNF‐α) with Elisa kits. Additionally, RAW 264.7 cells were lysed to analyze protein expression levels of iNOS and COX‐2 by Western blotting.

### Cellular Uptake of FITC‐HAND/FITC‐CND

HK‐2 cells were seeded at an appropriate density in a 6‐well plate and treated with 40 µg mL^−1^ FITC‐HAND, FITC‐CND, with or without an equivalent amount of uptake target competitive inhibitor (FA) according to the following groups: HAND + PBS treatment group, HAND + FA co‐treatment group, CND + PBS treatment group, and CND + FA co‐treatment group. After treatment, the cell plates were incubated in a 5% CO_2_, 37 °C incubator for 2 h. The old medium was then discarded, and each well was washed three times with 200 µL HBSS. The cells were then observed and photographed under a fluorescence microscope.

HK‐2 cells, H9c2 cells, and LX2 cells were seeded at an appropriate density in a 6‐well plate and treated with 40 µg mL^−1^ FITC‐HAND, FITC‐CND. After treatment, the cell plates were incubated in a 5% CO_2_, 37 °C incubator for 2 h. The old medium was then discarded, and each well was washed three times with 200 µL HBSS. The cells were then observed and photographed under a fluorescence microscope.

### Ethical Considerations

The animal experiments conducted in this study were approved by the Institutional Animal Care and Use Committee of Central South University (Ethical Permit Number: XMSB‐2024‐0263). Additionally, the human studies were approved by the Ethics Committee of Xiangya Hospital, Central South University (Ethical Permit Number: 2024030599). All procedures were performed in accordance with relevant guidelines and regulations to ensure the ethical treatment of all subjects involved.

### TEM Images of Mouse Kidney Tissue

After euthanizing the mice, the kidneys were carefully removed and quickly sliced into 1 × 1 × 1 mm^3^ pieces using a clean, pre‐cooled blade. These pieces were placed in a pre‐cooled electron microscope fixation solution. After 2 h of fixation, the tissues were washed three times with PBS (0.1 m, pH 7.4) for 15 min each. The tissues were then fixed in 1% OsO_4_ fixation solution in the dark for 2–3 h and washed again with PBS three times for 15 min each. The samples were dehydrated at room temperature using a gradient ethanol series (30, 50, 70, 80, 90, and 95%), treated for 15 min at each concentration, followed by treatment with 100% ethanol for 20 min and then transitioned to pure acetone for 20 min. The samples were then infiltrated with a mixture of embedding agent and acetone (V = 1:1) for 1 h, followed by a mixture of embedding agent and acetone (V = 3:1) for 3 h, and finally with pure embedding agent overnight. The infiltrated samples were embedded and heated at 70 °C overnight to obtain the embedded samples. These samples were sectioned into 6–8 µm slices using an ultramicrotome and placed on 150‐mesh copper grids coated with Formvar film. The sections were stained with saturated uranyl acetate solution in the dark for 8 min, then washed three times each with 70% ethanol and ultrapure water. They were further stained with lead citrate in the absence of CO_2_ for 8 min and washed three times with ultrapure water. The samples were dried using absorbent filter paper and left to dry overnight at room temperature. The next day, the samples were observed and photographed under a TEM.

### Detection of FITC‐HAND/FITC‐CND Distribution in Major Organs of Mice

KM mice were divided into the following groups: Control + HAND, Control + CND, AKI + HAND, and AKI + CND. The Control group mice were not subjected to any special treatment. In the AKI group, mice were injected with 1 mg kg^−1^ of FITC‐HAND or FITC‐CND via the tail vein 2 h after model induction. They were euthanized at 1, 3, 6, 9, 12, 24, 48, and 72 h post‐injection to collect major organs: heart, liver, spleen, lung, and kidney. Fluorescence and bright‐field images were captured using a Leica M165 FC stereo fluorescence microscope. The average optical density was analyzed using Image J software.

### In Vivo Tracking of Cy7‐HAND Distribution in Mice

KM mice were intravenously injected with Cy7‐HAND at a dose of 5 mg kg^−1^. Real‐time fluorescence imaging was conducted at 1, 3, 6, 9, 12, 24, 48, and 72 h post‐injection using an in vivo imaging system (IVIS) with excitation and emission filters set for Cy7. Mice were anesthetized before imaging to minimize movement and ensure accurate visualization. Fluorescence intensity was captured and analyzed in real‐time to assess the temporal biodistribution of Cy7‐HAND throughout the body.

### Construction of RM‐AKI Mouse Model

KM mice were acclimated for 3 days and then water‐deprived for 15 h (with free access to food). To construct the RM‐AKI mouse model, an equal amount of 50% glycerol (8 mL kg^−1^) was injected into the muscles of both hind limbs. After model establishment, the mice were allowed free access to water and food. The body weight of the mice was recorded before model establishment and before euthanasia to analyze weight changes.

### Animal Grouping and Administration

KM mice were divided into six groups: 1) Control group: normal mice injected with PBS via the tail vein; 2) AKI group: RM‐AKI mice injected with saline via the tail vein; 3) AKI + HAND (1 mg kg^−1^) group: RM‐AKI mice injected with 1 mg kg^−1^ HAND via the tail vein; 4) AKI + HAND (2 mg kg^−1^) group: RM‐AKI mice injected with 2 mg kg^−1^ HAND via the tail vein; 5) AKI + CND (2 mg kg^−1^) group: RM‐AKI mice injected with 2 mg kg^−1^ CND via the tail vein; 6) AKI + NAC (2 mg kg^−1^) group: RM‐AKI mice injected with 2 mg kg^−1^ NAC via the tail vein. The treatment was administered 2 h after model establishment, and samples were collected 24 h after administration for subsequent index detection. Each group contained six mice.

### Measurement Of Mouse Kidney Function Indicators

Mice were anesthetized with an intraperitoneal injection of 3% sodium pentobarbital 24 h after treatment, and blood was collected via the orbital sinus. The mice were then euthanized with an overdosage of anesthetic. The blood was left to stand for 30 min and then centrifuged (3000 rpm, 15 min) to separate and collect the serum. CRE and BUN levels were measured and calculated according to the kit instructions.

### H&E Staining of Mouse Kidney Sections

Paraffin blocks of mouse kidney tissue were sectioned into 4 µm slices. The slices were deparaffinized by immersion in xylene (15 min, 3 times), followed by gradient ethanol (5 min) and ultrapure water (15 min). Hematoxylin staining solution was applied to ensure complete coverage of the tissue, and after 10–15 min, excess dye was carefully rinsed off with running water. The sections were then differentiated in 1% hydrochloric acid ethanol solution for 1 s and immersed in water for 2 min. The eosin staining solution was applied for 5 min, followed by dehydration in anhydrous ethanol (2 min, 3 times) and clearing in xylene (2 min, 3 times). The slides were air‐dried in a fume hood for 30 min and then sealed with neutral gum. The stained sections were observed and photographed under a microscope.

### γ‐H2AX Foci Staining

Paraffin‐embedded kidney tissue sections (4 µm) were deparaffinized in xylene (15 min, 3 times), rehydrated through a graded ethanol series (5 min each), and rinsed in ultrapure water (15 min). The sections were then heated in citrate buffer (pH 6.0) at 95 °C for 15 min. After cooling, sections were permeabilized with 0.1% Triton X‐100 for 10 min. Following blocking with 5% BSA for 30 min, the sections were incubated with γ‐H2AX primary antibody overnight at 4 °C. The sections were then sealed with anti‐fluorescence quenching tablets containing DAPI. The samples were observed and photographed under a fluorescence microscope.

### Elisa Assay—Kidney Tissue Homogenate

Place kidney tissue samples from each group on ice. Cut an appropriate amount of tissue and add an appropriate volume of lysis buffer (selected based on experimental purpose). Add 2 sterile, enzyme‐free magnetic beads to each tube and homogenize at a frequency of 60 Hz for 180 s, repeating twice. Centrifuge the homogenized tissue samples at 12,000 g for 15 min at 4 °C, and retain the supernatant to obtain kidney tissue homogenate.

### Elisa Assay—Cell Supernatant

After trypsin digestion, collect the cells, centrifuge at 500 g for 10 min, and collect the supernatant.

### Elisa Assay—Cell Lysate

After trypsin digestion, collect the cells and centrifuge at 500 g for 10 min. Resuspend the cell pellet in an appropriate volume of cold lysis buffer (selected based on experimental purpose). Incubate the cell lysate on ice for 10–30 min to ensure complete cell lysis, occasionally vortexing or mixing gently. Then, centrifuge at 12,000 g for 15 min at 4 °C, and retain the supernatant to obtain the cell lysate.

### Elisa Assay—Mouse Serum

Centrifuge the blood at 3000 g for 15 min. Collect the supernatant to obtain the serum.

Then the relevant indexes were detected according to the instructions of each Elisa kit **Table** **2**.

**Table 2:** List of ELISA kits for mouse kidney tissue.

Primary/Secondary antibody	Company	Cat. No
KIM‐1	Elabscience	E‐EL‐M3039
HO‐1	Elabscience	E‐EL‐M3031
8‐OHdG	Elabscience	E‐EL‐0028
2′‐3′ cGAMP	Cayman	501700
TNF‐α	Elabscience	E‐EL‐M3063
IL‐1β	Elabscience	E‐EL‐M0037
IL‐6	Elabscience	E‐EL‐M0044
CXCL1	Elabscience	E‐EL‐M0018
CXCL2	Elabscience	E‐EL‐M0019
CCL2	Elabscience	E‐EL‐M3001
NGAL	Elabscience	E‐EL‐M0828

### DHE Staining of Mouse Kidney Sections

Kidney tissues were embedded in an Optimal Cutting Temperature (OCT) compound and then frozen. The kidneys were sectioned into ≈5 µm slices and mounted on cationic anti‐detachment slides. The superoxide anion fluorescent probe DHE was added to the tissue sections and incubated at room temperature in the dark for 30 min. The sections were washed three times with 0.01 m PBS to remove excess dye. The sections were then sealed with anti‐fluorescence quenching tablets containing DAPI. The samples were observed and photographed under a fluorescence microscope, and the fluorescence intensity was analyzed using Image J software.

### ROS Fluorescence Staining of HK‐2 Cells

Seed HK‐2 cells at an appropriate density in a 6‐well plate. Add an equal amount of PBS (control group) or 500 µm H_2_O_2_ (to induce oxidative damage) to each well. After 4 h of stimulation, add PBS/HAND/CND (40 µg mL^−1^) and incubate in a 5% CO_2_, 37 °C incubator for 24 h. Then, incubate the cells with the DCFH‐DA probe for 30 min and thoroughly wash away extra probes using a serum‐free cell culture medium. Discard the cell culture medium in the wells, and add 200 µL HBSS to each well. The cells were observed and photographed under a fluorescence microscope, and the fluorescence intensity was analyzed using Image J software.

### Detection of Oxidative Stress Indexes

Prepare kidney tissue homogenate according to the method described in “Detection of FITC‐HAND/FITC‐CND Distribution in Major Organs of Mice” in Experimental Section. Then, follow the instructions of the assay kits for each indicator to measure the levels of TBARS, MDA, GSH/GSSG, CAT, and SOD in the kidney tissues.

### RNA Sequencing Analysis

Total RNA was extracted from kidney tissue in the Control, AKI, and AKI+HAND group using Trizol according to the manufacturer's protocol. The subsequent analysis and data mining were performed on Dr. Tom's Multi‐omics Data mining system (https://biosys.bgi.com), the clean reads were mapped to the reference genome using HISAT2, and Bowtie2 was applied to align the clean reads to the gene set.

### Subcellular Localization of HAND

Seed HK‐2 cells at an appropriate density in a 24‐well plate. Incubate the cells with FITC‐HAND (40 µg mL^−1^) for 2 h, followed by 3 washes with PBS. Then, the cells were stained by ER‐tracker, Mito‐tracker, Golgi‐tracker, Lyso‐tracker, and Hoechst 33342 according to the instruction manual. Images were taken with a fluorescence microscope, and the colocalization values were calculated using GraphPad Prism 9.

### Molecular Docking

The molecular docking analysis of all the selected phytocompounds was subjected to MOE 2022 using the script standard method. PDB ID: 7vdd for TOM complex; 8scx for TIM23; 7mni for FG Nups.

### Detection of MMP of HK‐2 Cells

Seed HK‐2 cells at an appropriate density in a 6‐well plate. Add an equal amount of PBS (control group) or 500 µm H_2_O_2_ (to induce oxidative damage) to each well. After 4 h of stimulation, add PBS/HAND/CND (40 µg mL^−1^) and incubate in a 5% CO_2_, 37 °C incubator for 24 h. Remove the culture medium and wash the cells twice with PBS. Prepare a JC‐1 staining solution by diluting the JC‐1 dye in a suitable buffer as per the manufacturer's instructions. Add the JC‐1 staining solution to each well, ensuring the cells were completely covered, and incubate them at 37 °C for 20–30 min in a CO_2_ incubator. After incubation, remove the staining solution and wash the cells twice with PBS to remove excess dye. Finally, observe the cells under a fluorescence microscope. The JC‐1 dye will accumulate in healthy mitochondria, emitting red fluorescence, while in depolarized mitochondria, it will remain in monomeric form, emitting green fluorescence.

### Detection of ATP Production in HK‐2 Cells

Seed HK‐2 cells at an appropriate density in a 96‐well plate. Add an equal amount of PBS (control group) or 500 µm H_2_O_2_ (to induce oxidative damage) to each well. After 4 h of stimulation, add PBS/HAND/CND (40 µg mL^−1^) and incubate in a 5% CO_2_, 37 °C incubator for 24 h. Remove the culture medium and wash the cells twice with PBS. Lyse the cells using lysis buffer from the kit, ensuring complete lysis by incubating the plate for 5 min at room temperature. Prepare the ATP assay reagent according to the manufacturer's instructions. Add the reagent to each well containing the cell lysate, and mix gently to ensure a proper reaction. Incubate the plate for 10 min at room temperature to stabilize the luminescent signal. Measure the luminescence using an enzyme‐labeled instrument. The luminescence intensity was directly proportional to the ATP concentration in the sample, allowing for quantification of ATP production in the HK‐2 cells.

### Mito‐SOX Fluorescence Detection and Flow Cytometry in HK‐2 Cells

Seed HK‐2 cells at an appropriate density in a 6‐well plate. Add an equal amount of PBS (control group) or 500 µm H_2_O_2_ (to induce oxidative damage) to each well. After 4 h of stimulation, add PBS/HAND/CND (40 µg mL^−1^) and incubate in a 5% CO_2_, 37 °C incubator for 24 h.

For fluorescence microscopy, wash the cells with PBS, then add the Mito‐SOX reagent to the well according to the manufacturer's instructions, and incubate at 37 °C for 10–30 min in the dark. After incubation, wash the cells with PBS to remove excess dye and observe under a fluorescence microscope using appropriate filters.

For flow cytometry, harvest and wash the cells with PBS, then add the Mito‐SOX reagent to the well according to the manufacturer's instructions, and incubate at 37 °C for 10–30 min in the dark. After incubation, wash the cells with PBS to remove excess dye transfer the stained cells to a flow cytometry tube, and analyze them using a flow cytometer set to detect the specific fluorescence emission of Mito‐SOX.

### Western Blot

Collect protein samples from tissues or cells according to the method described in “Detection of FITC‐HAND/FITC‐CND Distribution in Major Organs of Mice” in Experimental Section using RIPA buffer as lysate. Determine protein concentration using the BCA method. Prepare an appropriate concentration of separating gel according to the protein molecule and 5% stacking gel. Load 20 µg of protein sample into each well and perform constant voltage electrophoresis (80 V, 30 min). Transfer the electrophoresed protein samples onto a polyvinylidene fluoride (PVDF) membrane using a constant current wet transfer method (250 mA). Place the PVDF membrane in an incubation box and add TBST (Tris‐Buffered Saline and Tween 20) solution containing 5% non‐fat milk powder. Gently shake on a shaker for blocking for 60 min. Wash the blocked PVDF membrane three times with TBST solution, 10 min each time. After gently removing the surface liquid, incubate the membrane in primary antibody working solution at 4 °C overnight. Wash the incubated PVDF membrane three times with TBST, 10 min each time. After gently removing the surface liquid, place the membrane in the corresponding rabbit or mouse secondary antibody incubation box and incubate at room temperature for 60 min. Wash three times after incubation. Place the PVDF membrane in a developing tray and evenly apply the developing solution (Solution A:Solution B = 1:1) to its surface, ensuring complete and uniform coverage. Capture the image using the ChemiDoc XRS+ imaging system and perform grayscale scanning with Image Lab software. The used antibodies are shown in **Table** **3**.

**Table 3:** List of antibodies.

Primary/Secondary antibody	Company	Cat. No	Dilution
Bax	Abcam	ab32503	1:5000
Bcl2	Affinity	BF9103	1:1000
Caspase 3	Proteintech	19677‐1‐AP	1:2000
Cleaved Caspase 3	Wanleibio	WL02117	1:500
Cytochrome C	Proteintech	10993‐1‐AP	1:5000
cGAS	Proteintech	29958‐1‐AP	1:1000
STING	Proteintech	19851‐1‐AP	1:1000
IRF3	Affinity	DF6895	1:1000
P‐IRF3	Affinity	AF2436	1:1000
P65	Affinity	BF8005	1:1000
P‐P65	Affinity	AF2006	1:1000
Tubulin	Affinity	AF7011	1:5000
Goat anti Rabbit IgG H&L (HRP)	Abclonal	AS014	1:6000
Goat anti mice IgG H&L (HRP)	Abclonal	AS003	1:5000
dsDNA	Abcam	AB27156	1:500
TOM20	Proteintech	11802‐1‐AP	1:500
8‐OHdG	Abcam	AB48508	1:1000
F4/80	Proteintech	28463‐1‐AP	1:1000
Ly‐6G	Abcam	AB25377	1:1000
Goat anti‐Rabbit IgG (H+L) Cross‐Adsorbed Secondary Antibody, Alexa Fluor™ 555	Invitrogen	A21428	1:500
Goat anti‐Mouse IgG (H+L) Highly Cross‐Adsorbed Secondary Antibody, Alexa Fluor™ 488	Invitrogen	A11029	1:500
FA	Proteintech	23355‐1‐AP	1:1000

### Annexin V/PI Flow Cytometry

Seed HK‐2 cells at an appropriate density in a 12‐well plate. Add an equal amount of PBS (control group) or 500 µm H_2_O_2_ (to induce oxidative damage) to each well. After 4 h of stimulation, add PBS/HAND/CND (40 µg mL^−1^) and incubate in a 5% CO_2_, 37 °C incubator for 24 h. Collect and wash the HK‐2 cells, then resuspend them in a binding buffer. Add fluorescently labeled Annexin V and propidium iodide (PI) to the cell suspension and incubate at room temperature in the dark for 10–15 min. Subsequently, analyze the cells using flow cytometry, distinguishing early and late apoptotic cells based on the fluorescence signals of Annexin V and PI.

### IHC Staining

Prepare, fix, and section the kidney tissue, and then deparaffinize and rehydrate the sections (xylene × 2, 15 min; anhydrous ethanol, 10 min; 95% ethanol, 5 min; 80% ethanol, 5 min; 70% ethanol, 5 min; ultra‐pure water, 1 min; PBST × 3, 2 min). Preheat the water bath to 95 °C in advance, dip the slices into 1XTris‐EDTA repair solution, cool them naturally after the water bath for 15 min, and clean them with PBST 3 times (3 min/ time). Add an appropriate amount of endogenous catalase blocker to the tissue sample (completely covering the tissue), incubate in a wet box at 37 °C for 20 min, and wash them with PBST 3 times (3 min/ time). The sections are then blocked with 5% BSA and incubated with a specific primary antibody, washed, and incubated with a secondary antibody. Use DAB as a chromogenic substrate for color development, followed by hematoxylin counterstaining. Finally, the sections are dehydrated, cleared, and sealed for microscopic examination.

### TUNEL Staining

Prepare, fix, and section the kidney tissue, and then deparaffinize and rehydrate the sections (xylene × 2, 15 min; anhydrous ethanol, 10 min; 95% ethanol, 5 min; 80% ethanol, 5 min; 70% ethanol, 5 min; ultra‐pure water, 1 min; PBST × 3, 2 min). Prepare Proteinase K working solution, TdT incubation buffer, Equilibration Buffer, 0.1% Triton X‐100, and 5 mg kg^−1^ BSA according to the instructions. Add an appropriate amount of Proteinase K working solution to the samples and incubate at room temperature for 20 min, ensuring complete and even coverage of the samples. Rinse the samples with PBST 2–3 times for 5 min each. Add 60 µL of Equilibration Buffer to each sample and incubate at room temperature for 20 min. Remove most of the Equilibration Buffer, then add 50 µL of TdT incubation buffer to the samples and incubate in a humidified box at room temperature in the dark for 60 min. Remove the incubation buffer and wash with PBS containing 0.1% Triton X‐100 and 5 mg kg^−1^ BSA three times for 5 min each. Add an anti‐fluorescence quencher containing DAPI to the samples and cover them with coverslips. Observe and photograph the samples under a fluorescence microscope, and analyze fluorescence intensity using Image J.

### IF Staining for Kidneys and HK‐2 Cells

Prepare, fix, and section the kidney tissue, and then deparaffinize and rehydrate the sections (xylene × 2, 15 min; anhydrous ethanol, 10 min; 95% ethanol, 5 min; 80% ethanol, 5 min; 70% ethanol, 5 min; ultra‐pure water, 1 min; PBST × 3, 2 min). Preheat the water bath to 95 °C in advance, dip the slices into Tris‐EDTA repair solution, cool them naturally after the water bath for 15 min, and clean them with PBST 3 times (3 min/ time). Incubate the sections with 0.1% TritonX‐100 in PBS at room temperature for 10 min. The sections were then blocked with 5% BSA and incubated with primary antibody overnight at 4 °C. After washing with PBS, apply fluorescent secondary antibodies and co‐incubate at room temperature in the dark for 60 min. Add an anti‐fluorescence quencher containing DAPI to the samples and cover them with coverslips. Observe and photograph the samples under a fluorescence microscope.

Culturing HK‐2 cells on circle microscope cover glass placed in a 24‐well plate until they reach the desired confluency. Fix the cells with 4% paraformaldehyde for 15 min, then permeabilize them with 0.1% Triton X‐100 in PBS for 10 min. Block the cells with 5% BSA for 30 min at room temperature to prevent nonspecific binding. Incubate the cells with primary antibodies overnight at 4 °C. After washing with PBS, apply the appropriate fluorescent secondary antibodies for 1 h at room temperature. Wash the microscope cover glass with PBS, mount them onto glass slides with an antifade mounting medium, and seal with an anti‐fluorescence quencher containing DAPI. Finally, use a confocal microscope to visualize and capture images of the stained cells.

### Mtdna Copy Number

mtDNA copy number was estimated by quantitative real‐time PCR. Mitochondria were separated and DNA was then extracted from HK‐2 cells with a Mitochondrial separation and protein extraction kit (Proteintech) and MolPure Cell/Tissue DNA Kit (Yeasen, CHINA), respectively. Mitochondrially encoded Cytochrome C Oxidase II (MT‐CO_2_) was measured using the following primers: Forward 5′‐CCT CCC ATT CAT TAT CGC CGC CCT TGC‐3′ and Reverse 5′‐GTC TGG GTC TCC TAG TAG GTC TGG GAA‐3′. The nuclear gene ACTB (β‐actin) was measured using the following primers: Forward 5′‐GGA GAT TAC TGC CCT GGC TCC TA‐3′ and Reverse 5′‐GAC TCA TCG TAC TCC TGC TTG CTG‐3′. The assay was performed in a total reaction volume of 20 µL containing 10 µL of SYBR Premix Ex Taq, 6.8 µL of ddH_2_O, 0.4 µL of each primer (10 µm), 0.4 µL of ROX Reference DyeII (50x) and 2 µL of DNA template. The reaction was performed at 95 °C for 30 s, followed by 40 cycles of 5 s at 95 °C and 34 s at 65 °C, 95 °C for 15 s, 1 min at 60 °C, and 15 s at 95 °C. Each sample was assayed in triplicate, and the relative mtDNA copy number was calculated with ΔΔCt and normalized to ACTB.

### Picogreen Dsdna Quantitation

Seed HK‐2 cells at an appropriate density in a 6‐well plate. Add an equal amount of PBS (control group) or 500 µm H_2_O_2_ (to induce oxidative damage) to each well. After 4 h of stimulation, add PBS/HAND/CND (40 µg mL^−1^) and incubate in a 5% CO_2_, 37 °C incubator for 24 h. After trypsin digestion, collect the cells and centrifuge at 500 g for 10 min. Isolate mitochondria and extract dsDNA from both mitochondrial and cytosolic fractions according to the protocol provided by the kits. Ensure both mitochondrial and cytosolic fractions were ready for dsDNA quantitation. Prepare a series of standard DNA dilutions using a known concentration of dsDNA to generate a standard curve. Add 100 µL of the extracted mitochondrial or cytosolic DNA samples into a 96‐well plate. Add 100 µL of PicoGreen reagent to each well and mix gently. Incubate the plate in the dark for 5–10 min at room temperature. Measure the fluorescence intensity at an excitation wavelength of 480 nm and emission wavelength of 520 nm using a fluorescence microplate reader. Calculate the concentration of dsDNA in the samples using the standard curve.

### Biocompatibility Evaluation—Short‐Term

Healthy KM mice were randomly divided into two groups (*n* = 6). The control group received an intravenous injection of 100 µL 1×PBS, while the HAND group received an intravenous injection of 100 µL HAND solution (10 mg kg^−1^). Mice were euthanized 24 h after injection, and blood was collected along with major organs (heart, liver, spleen, lungs, and kidneys).

### Biocompatibility Evaluation—Long‐Term

Healthy KM mice were randomly divided into two groups (*n* = 6). The control group received an intravenous injection of 100 µL 1×PBS, while the HAND group received an intravenous injection of 100 µL HAND solution (2 mg kg^−1^). HAND/PBS was injected once a week, and mice were euthanized after 4 weeks. Blood was collected, and major organs (heart, liver, spleen, lungs, and kidneys) were harvested.

### Biocompatibility Evaluation—H&E Staining

The H&E procedure for major organs was the same as in “Detection of FITC‐HAND/FITC‐CND Distribution in Major Organs of Mice” in Experimental Section.

### Biocompatibility Evaluation—Hematological Parameter Testing

Whole blood samples from mice were analyzed using a hematology analyzer. Specific parameters included: Platelet‐related parameters: Platelet count (PLT), Mean Platelet Volume (MPV), Platelet Distribution Width (PDW), and Plateletcrit (PCT). Erythrocyte‐related parameters: Red Blood Cell count (RBC), Hematocrit (HCT), Hemoglobin content (HGB), Mean Corpuscular Volume (MCV). Leukocyte‐related parameters: Neutrophilic granulocyte count (GRAN), White Blood Cell count (WBC), Monocyte count (MON), and Lymphocyte count (LYM).

### Biocompatibility Evaluation—Liver and Kidney Function Testing

Serum levels of CRE, BUN, AST, and Alanine ALT were measured according to the kit instructions.

### Biocompatibility Evaluation—Inflammatory Factors Detection

ELISA kits were used to measure levels of TNF‐α, IL‐6, and IL‐1β in mouse serum.

### Statistical Analysis

All data were pre‐processed to check for normality using the Shapiro‐Wilk test, and outliers were identified and removed based on Grubbs’ test. Quantitative data are presented as mean ± standard deviation (SD). Each experiment was independently performed at least three times. For statistical comparisons between the two groups, an independent samples t‐test (two‐sided) was applied. For comparisons among multiple groups, one‐way ANOVA was used, followed by post‐hoc multiple comparisons with the SNK test. The significance level (alpha) was set at 0.05, and a *p*‐value < 0.05 was considered statistically significant. Assumptions of homogeneity of variances were verified using Levene's test. All statistical analyses were conducted using SPSS 23.0 software. The sample size (n) for each analysis is indicated in the figure legends.

## Conflict of Interest

The authors declare no conflict of interest.

## Author Contributions

All authors contributed to the article and approved the submitted version.

## Supporting information



Supporting Information

## Data Availability

The data that support the findings of this study are available from the corresponding author upon reasonable request.
