# Supplementary material for: Hierarchical Targeting Nanodrug with Holistic DNA Protection for Effective Treatment of Acute Kidney Injury
Source: Adv Sci (Weinh). 2024 Dec 20;12(6):2411254. doi: 10.1002/advs.202411254 (PMC11809360; doi:10.1002/advs.202411254)
Supplement: Supplementary file 1 — Supporting Information [file ADVS-12-2411254-s001.docx]

**Supplement Information**

**Hierarchical Targeting Nanodrug with Holistic DNA Protection for Effective Treatment of Acute Kidney Injury**

Qiaohui Chen^1,2^, Yongqi Yang^2,3^, Xiaohong Ying^2,3^, Changkun Huang^4^, Jianlin Chen^5,6^, Jue Wang^2,3^, Ziyu Wu^2,3^, Wan Zeng^2,3^, Chenxi Miao^2,3^, Xiaojing Shi^2,3^, Yayun Nan^7^, Qiong Huang^1,8*^, Kelong Ai^2,3,9*^

^1^Department of Pharmacy, Xiangya Hospital, Central South University, Changsha, 410008, China.

^2^Xiangya School of Pharmaceutical Sciences, Central South University, Changsha, 410013, China.

^3^Hunan Provincial Key Laboratory of Cardiovascular Research, Xiangya School of Pharmaceutical Sciences, Central South University, Changsha, 410013, China.

^4^Department of Urology, The Second Xiangya Hospital, Central South University, Changsha, Hunan, 410011, China.

^5^Department of Pancreatic Surgery, Xiangya Hospital, Central South University, Changsha, 410008, China

^6^Department of General Surgery, Xiangya Hospital, Central South University, Changsha, 410008, China

^7^Geriatric Medical Center, People's Hospital of Ningxia Hui Autonomous Region, Yinchuan, Ningxia, 750002, China.

^8^National Clinical Research Center for Geriatric Disorders, Xiangya Hospital, Central South University, Changsha, 410008, China.

^9^Key Laboratory of Aging-related Bone and Joint Diseases Prevention and Treatment, Ministry of Education, Xiangya Hospital, Central South University, Changsha, 410008, China.

*Corresponding authors: qionghuang@csu.edu.cn (Prof. Qiong Huang), aikelong@csu.edu.cn (Prof. Kelong Ai)


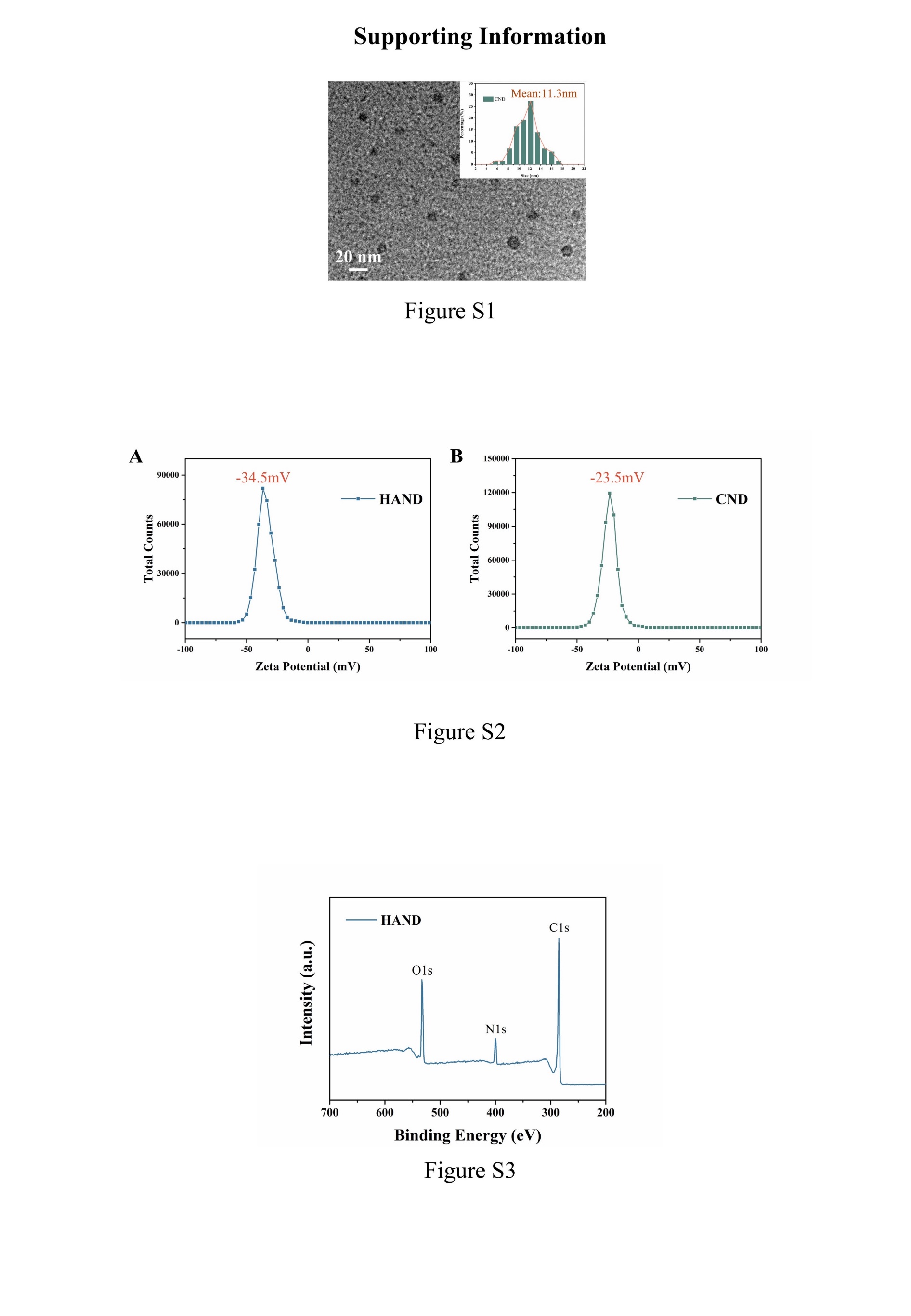


**Figure S1.** TEM image of CND. Insert: Size distribution statistics of CND.


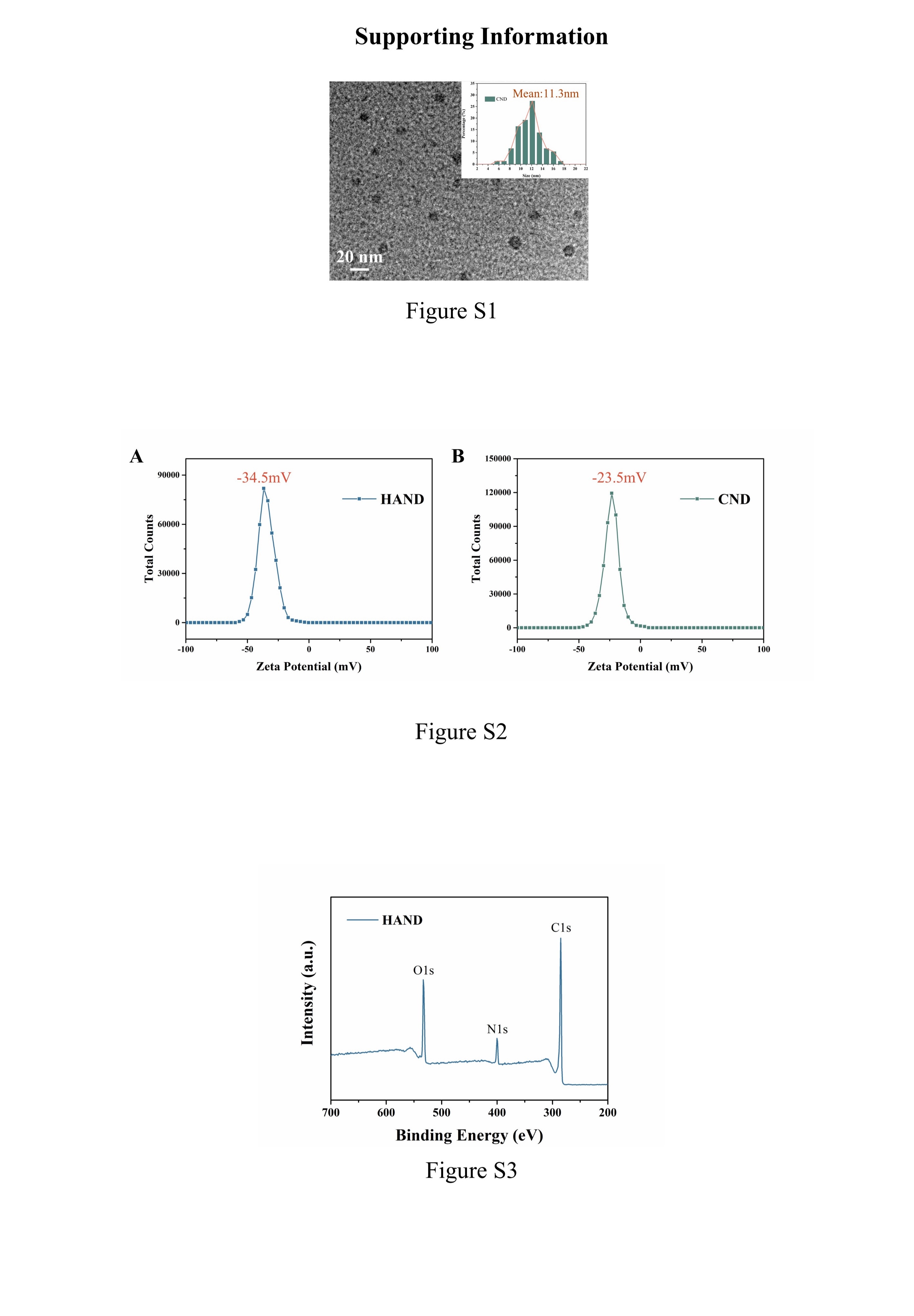


**Figure S2.** Zeta potential determination of HAND (A) and CND (B).


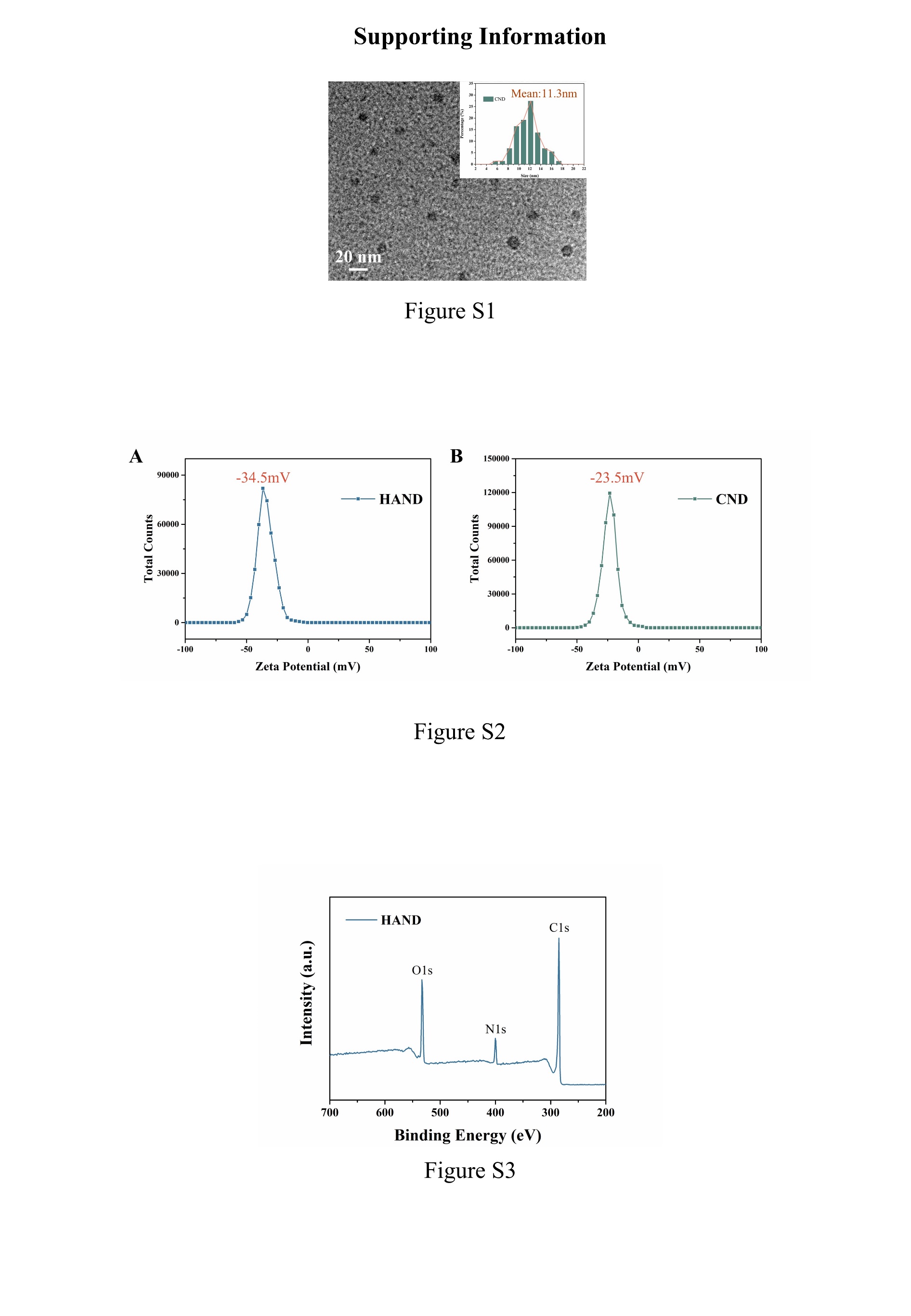


**Figure S3.** XPS spectrum of HAND.


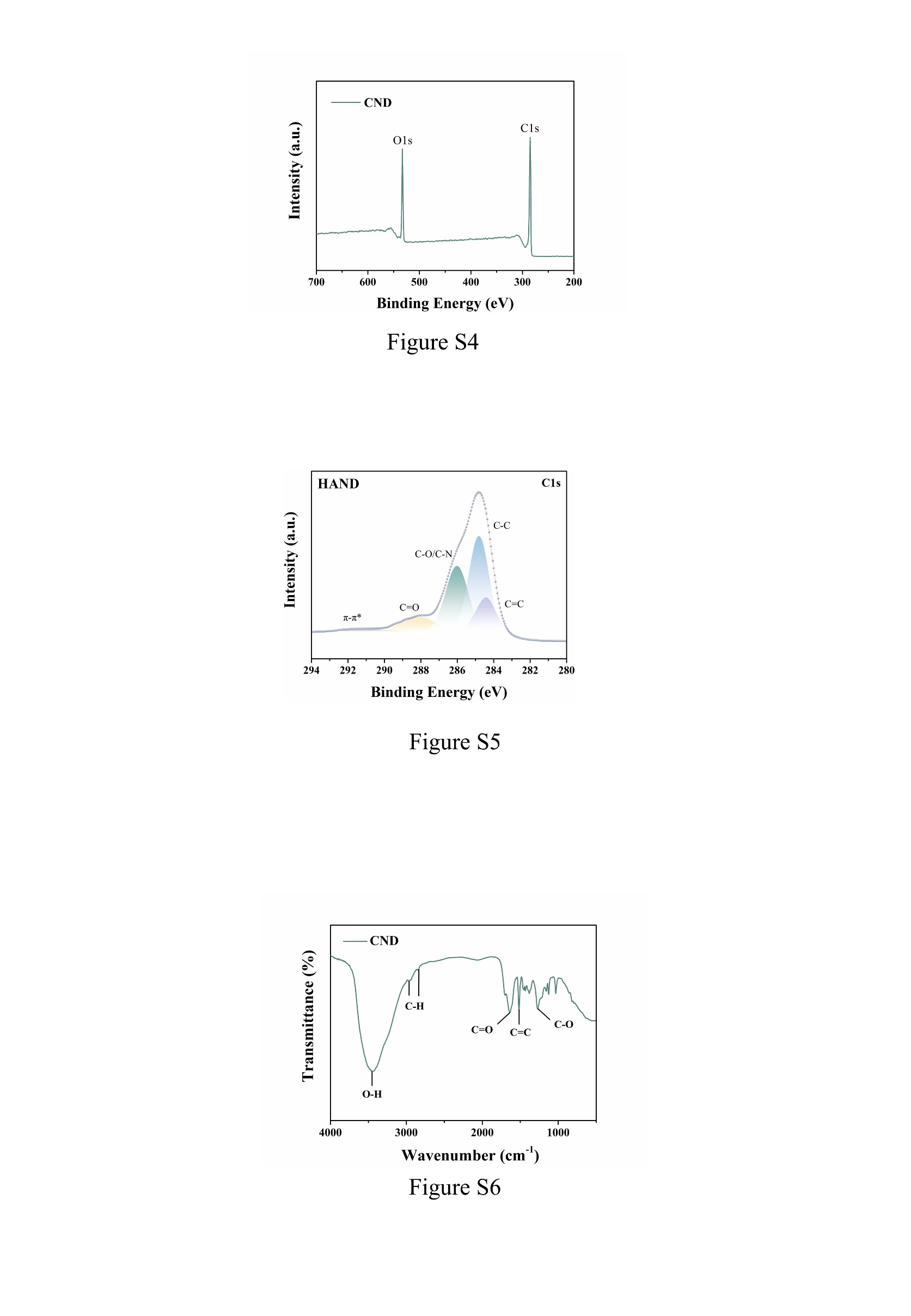


**Figure S4.** XPS spectrum of CND.


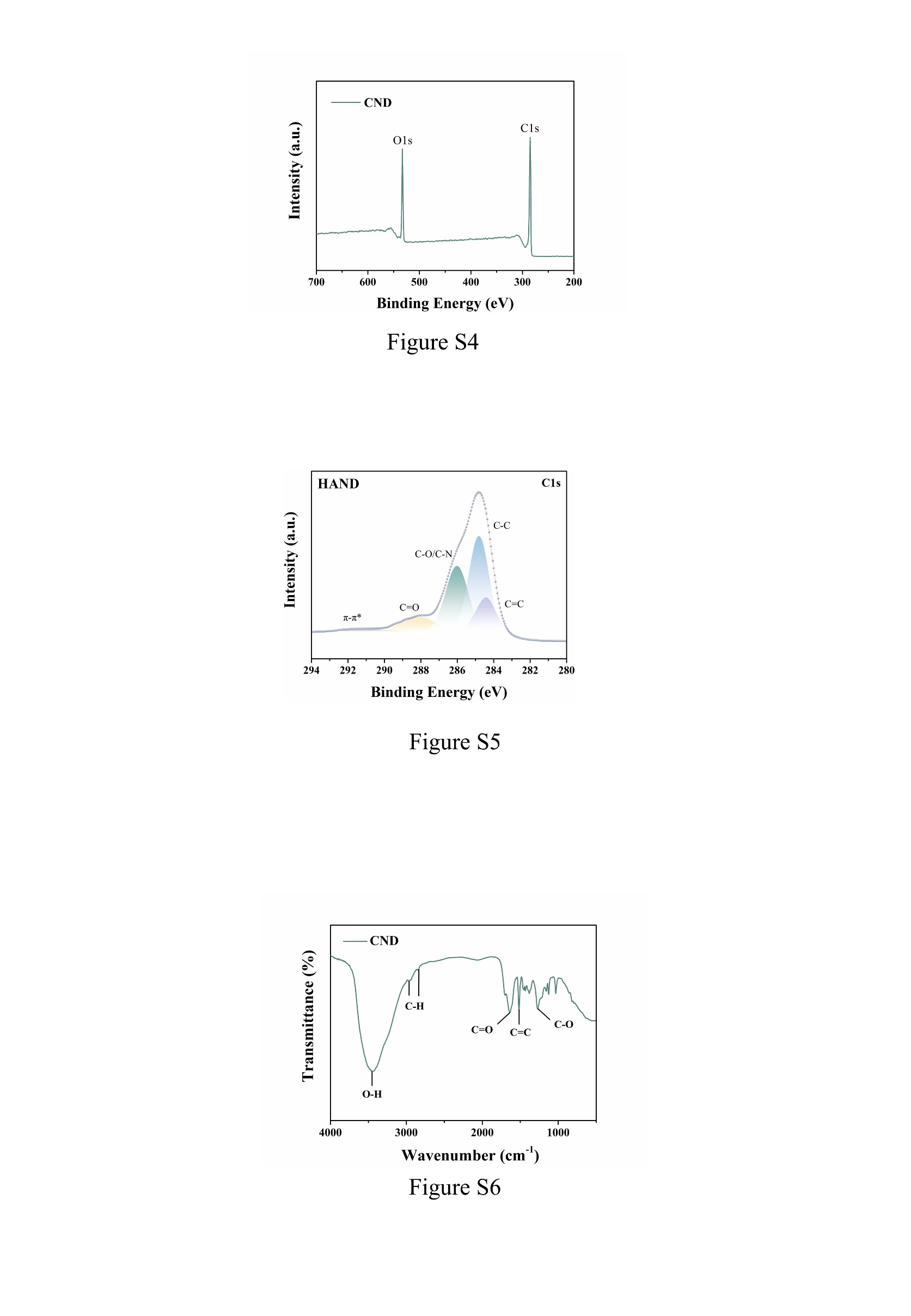


**Figure S5.** XPS C1s spectrum of HAND.


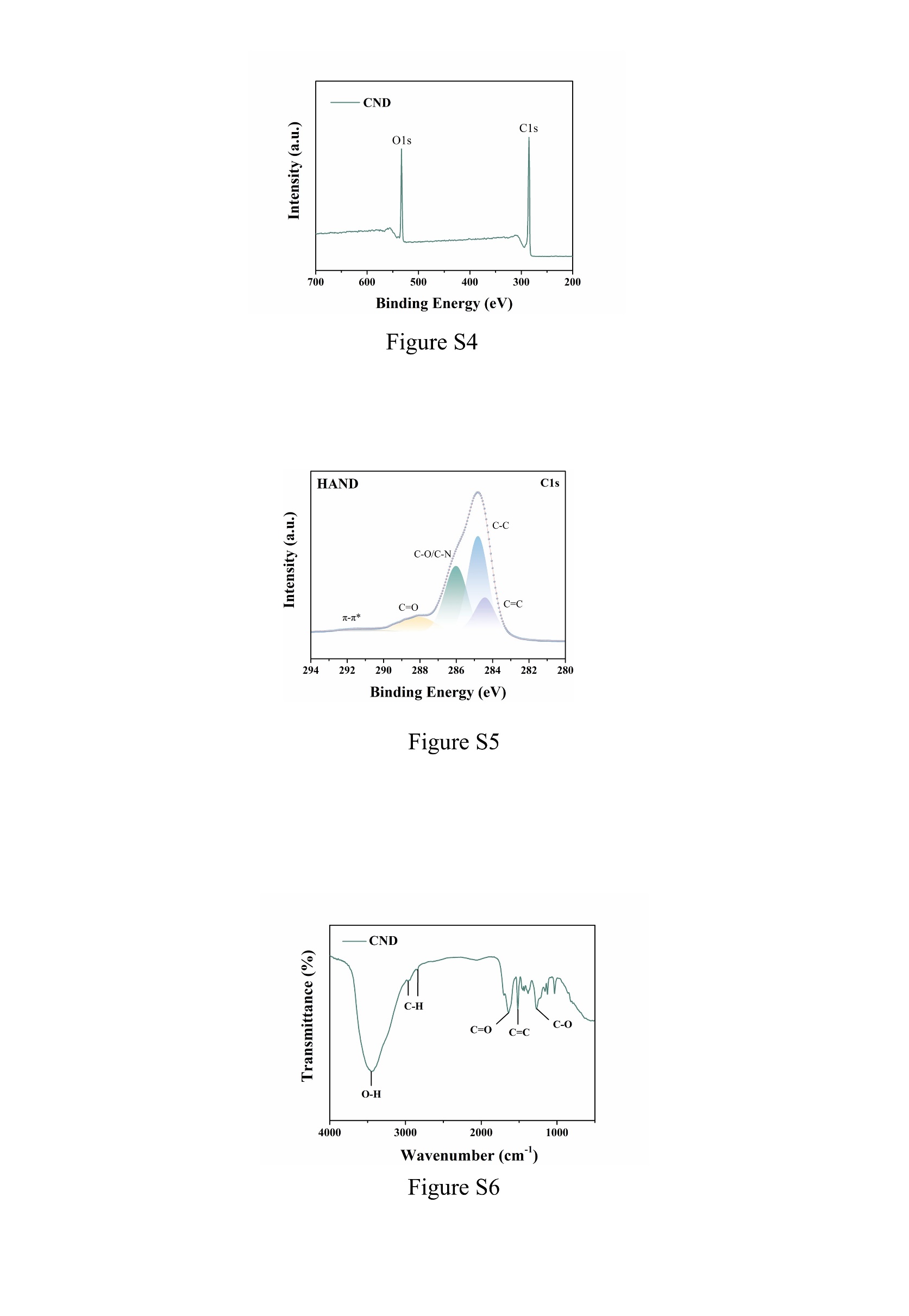


**Figure S6.** FT-IR spectrum of CND.


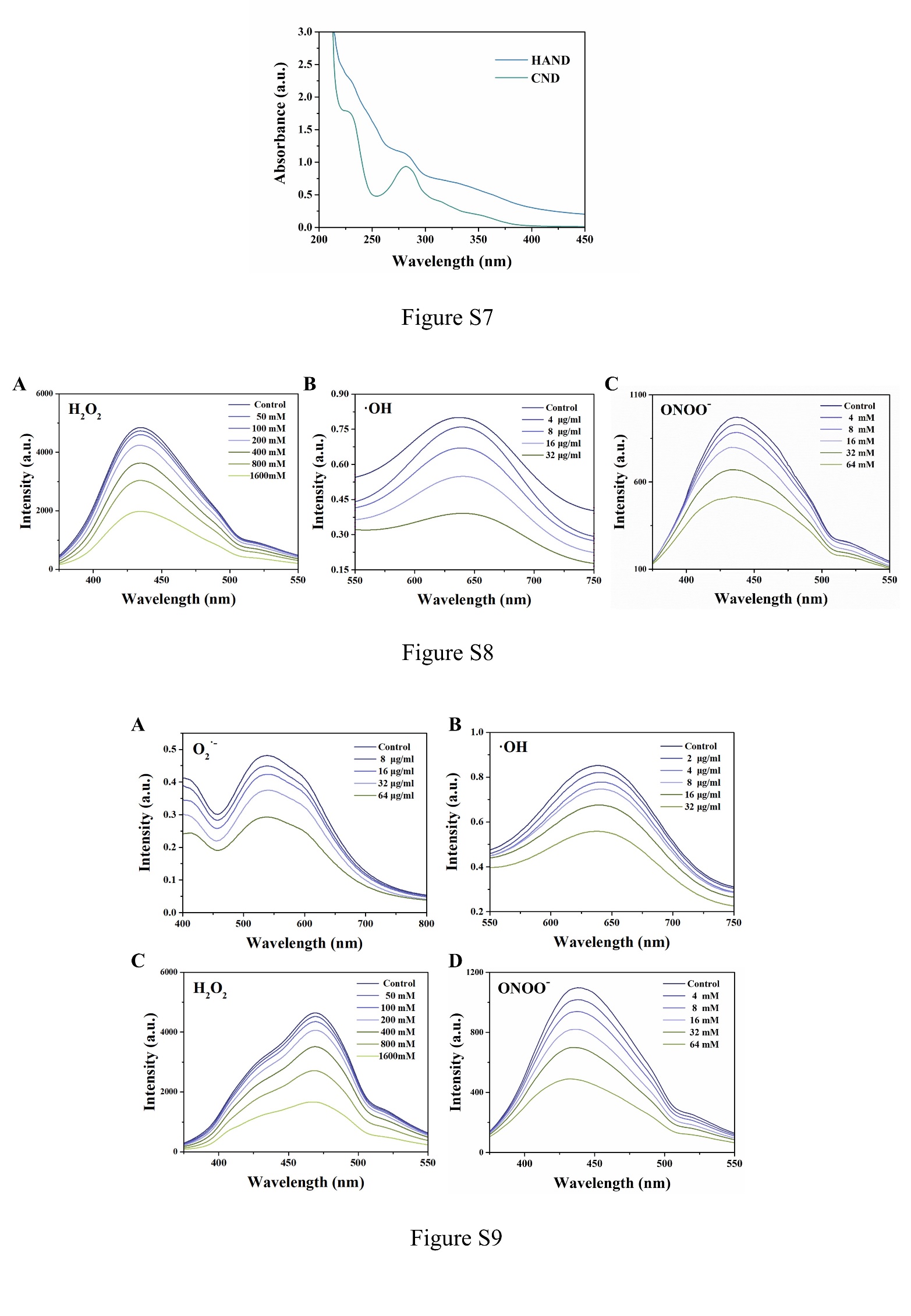


**Figure S7.** UV-Vis spectrum of HAND and CND.


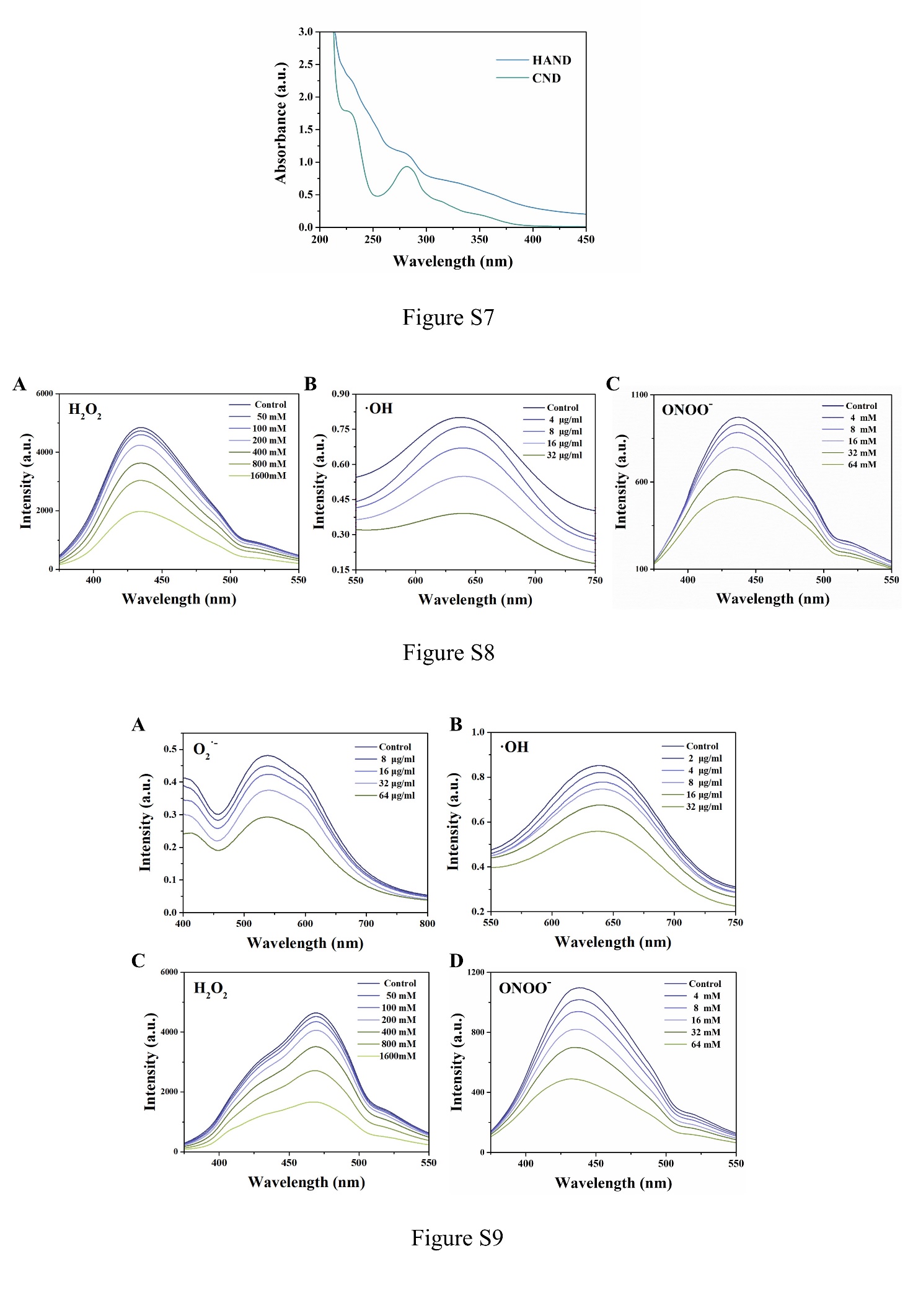


**Figure S8.** (A) Fluorescence emission spectra of HAND reacting with different concentrations of H_2_O_2_. (B) UV absorption spectra of ·OH reacting with different concentrations of HAND. (C) Fluorescence emission spectra of HAND reacting with different concentrations of ONOO^-^.


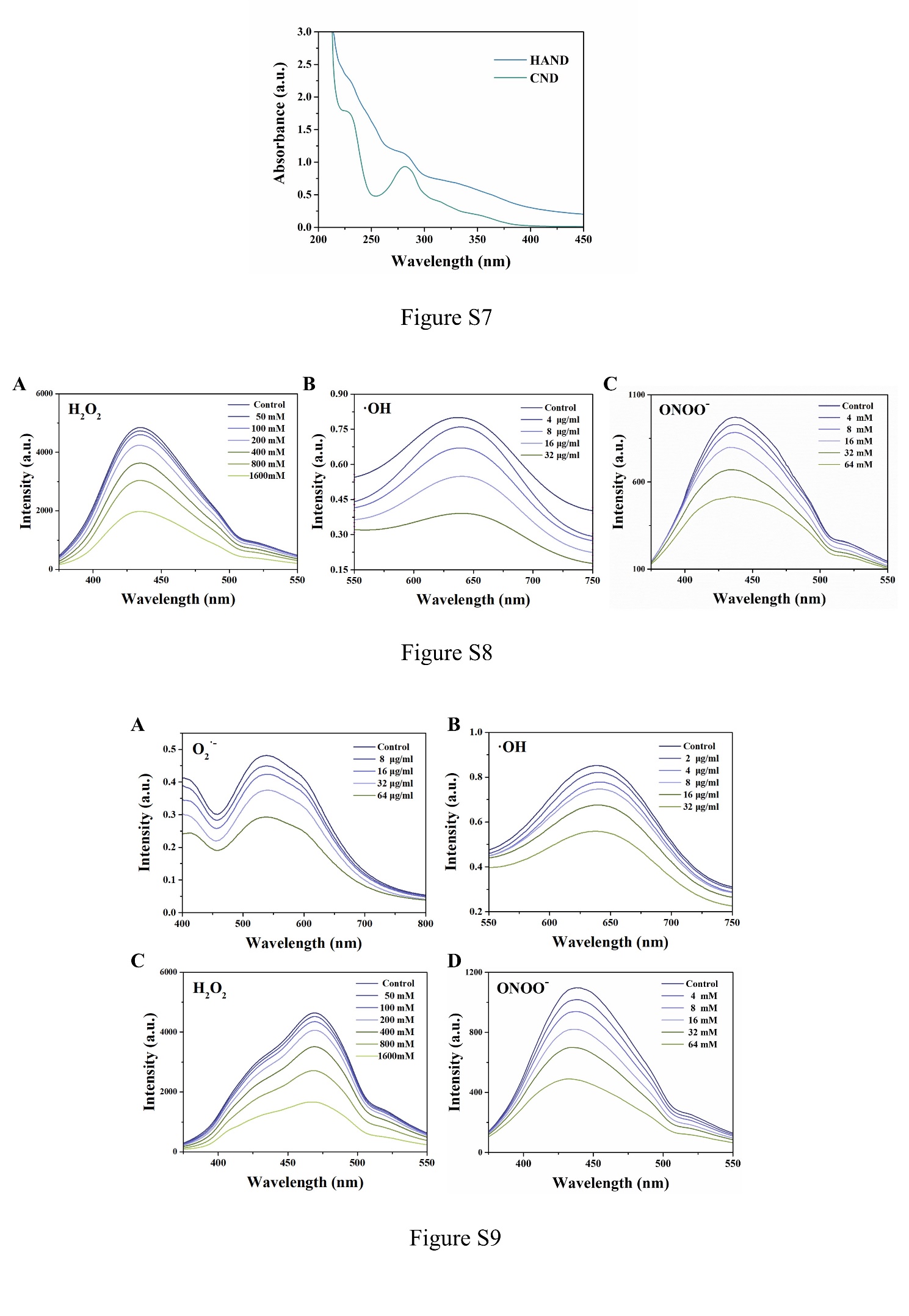


**Figure S9.** (A) UV absorption spectra of O_2_^·-^ reacting with different concentrations of CND. (B) UV absorption spectra of ·OH reacting with different concentrations of CND. (C) Fluorescence emission spectra of CND reacting with different concentrations of H_2_O_2_. (D) Fluorescence emission spectra of CND reacting with different concentrations of ONOO^-^.


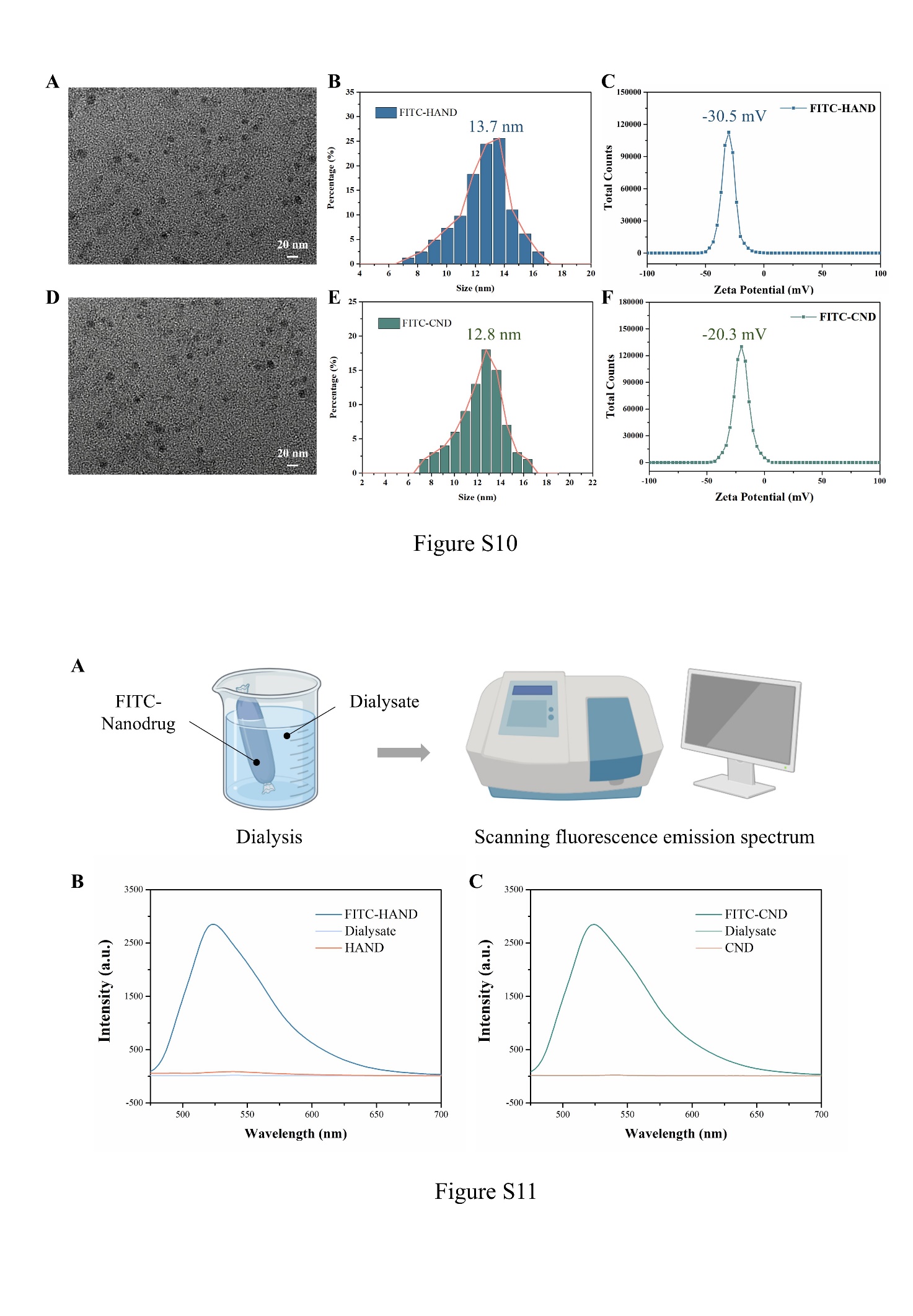


**Figure S10.** (A) TEM image of FITC-HAND. (B) Size distribution statistics of FITC-HAND. (C) Zeta potential determination of FITC-HAND. (D) TEM image of FITC-HAND. (E) Size distribution statistics of FITC-HAND. (F) Zeta potential determination of FITC-HAND.


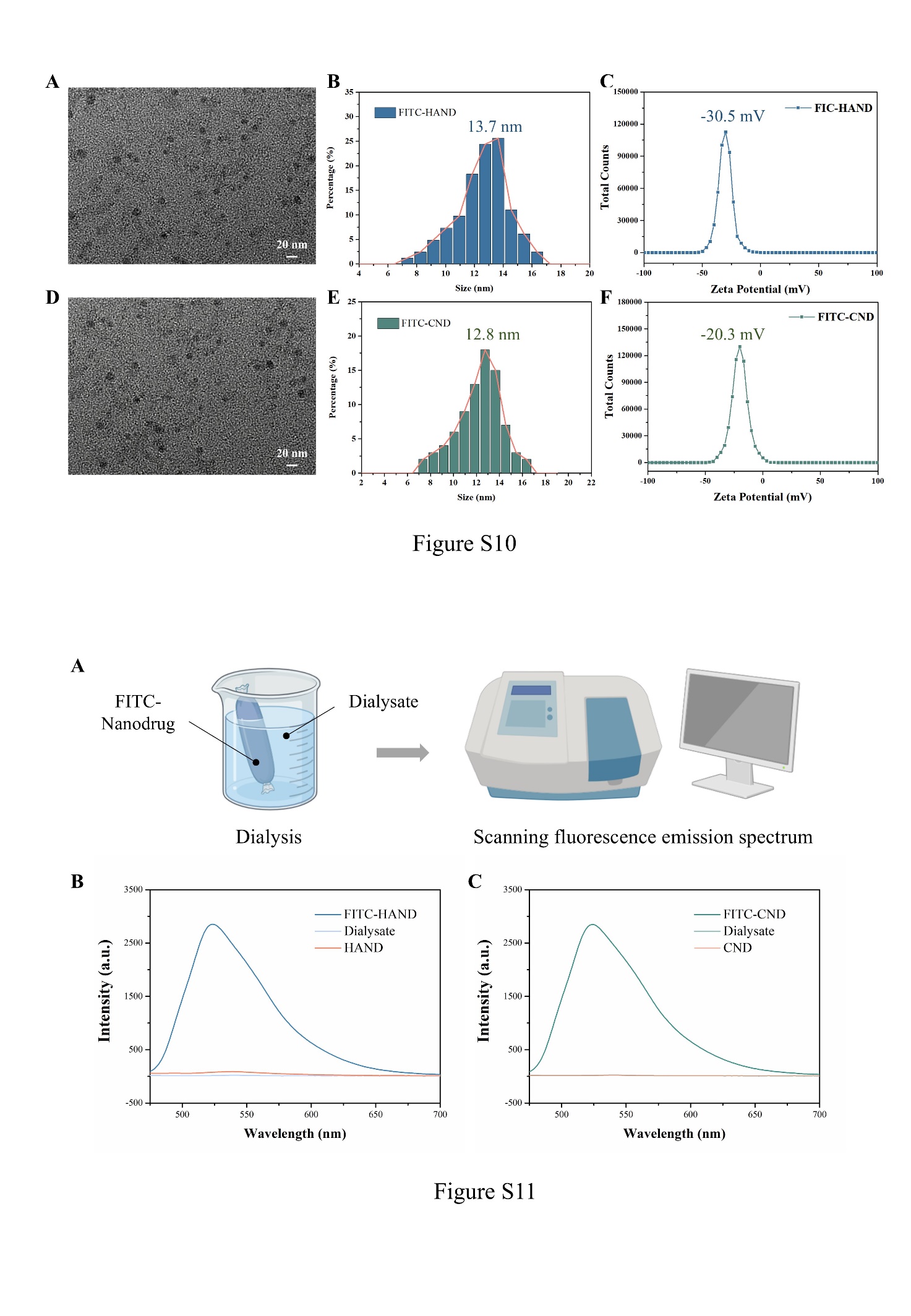


**Figure S11.** (A) Schematic illustration of dialysis purification of FITC-labeled nanodrugs. (B) Fluorescence emission spectra of FITC-HAND components before and after dialysis. (C) Fluorescence emission spectra of FITC-CND components before and after dialysis.


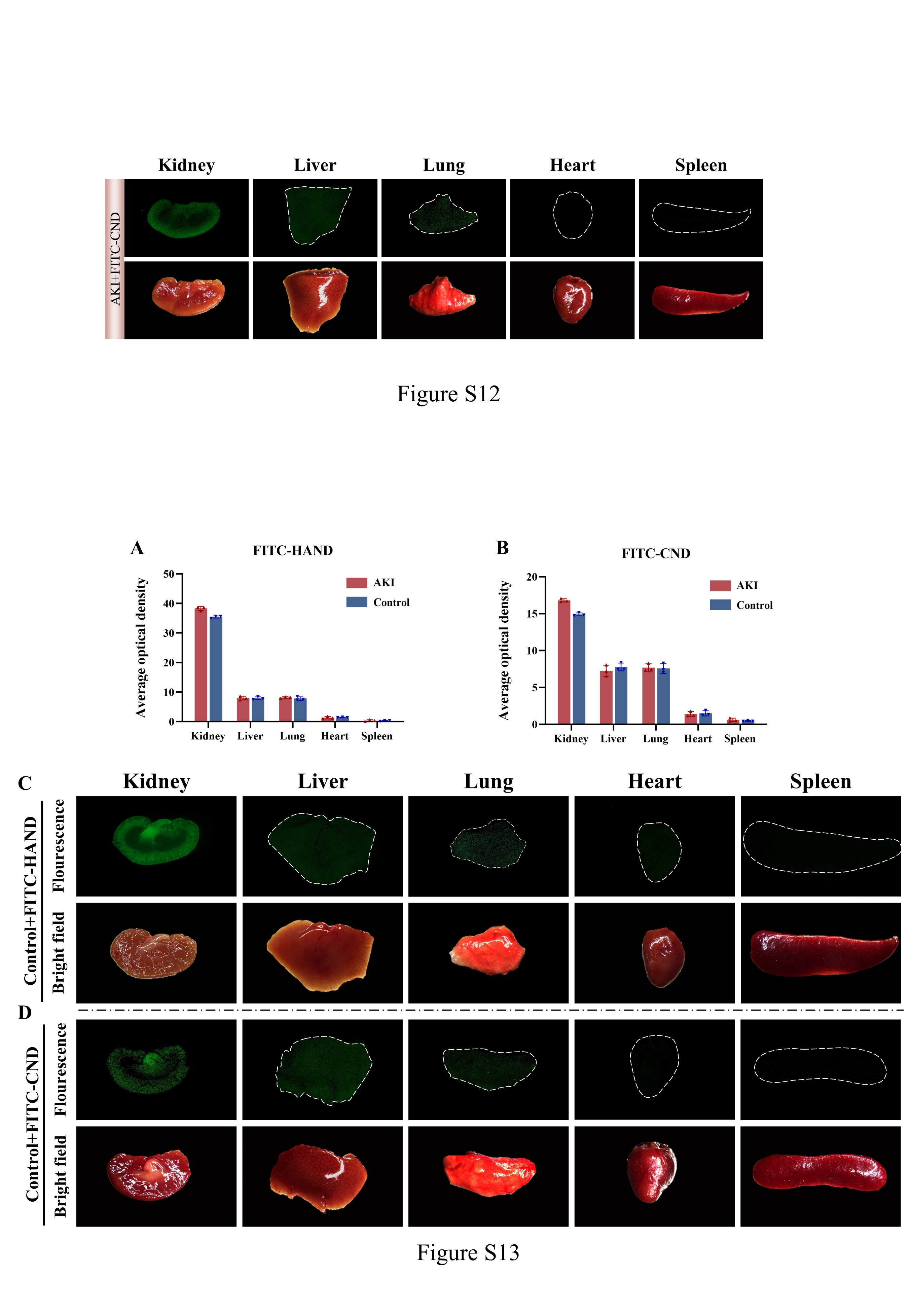


**Figure S12.** Representative fluorescence images of different organs in AKI mice 6 hours after intravenous injection of CND.


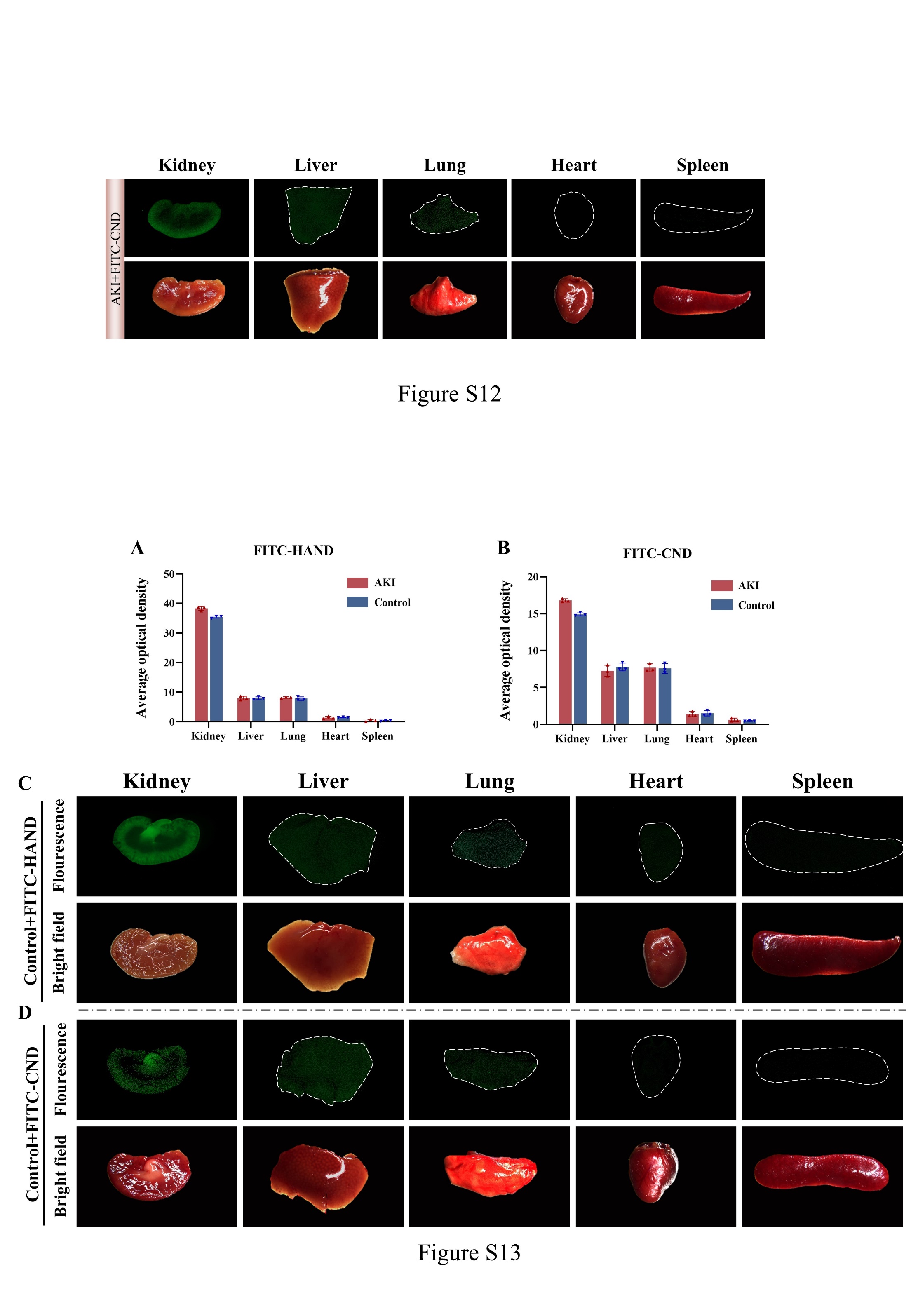


**Figure S13.** (A) The fluorescence intensity in different organs 6 hours after FITC-HAND injection in different groups. (B) The fluorescence intensity in different organs 6 hours after FITC-CND injection in different groups. (C-D) Representative fluorescence images of different organs in Control mice 6 hours after intravenous injection of FITC-HAND (C) and FITC-CND (D). Data are presented as mean ± SD. n=3.


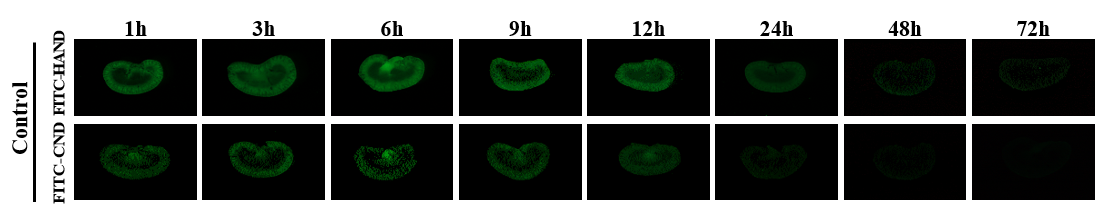


**Figure S14.** Representative fluorescence images of kidneys in Control mice at various time points (1-72h) after intravenous injection of drugs.


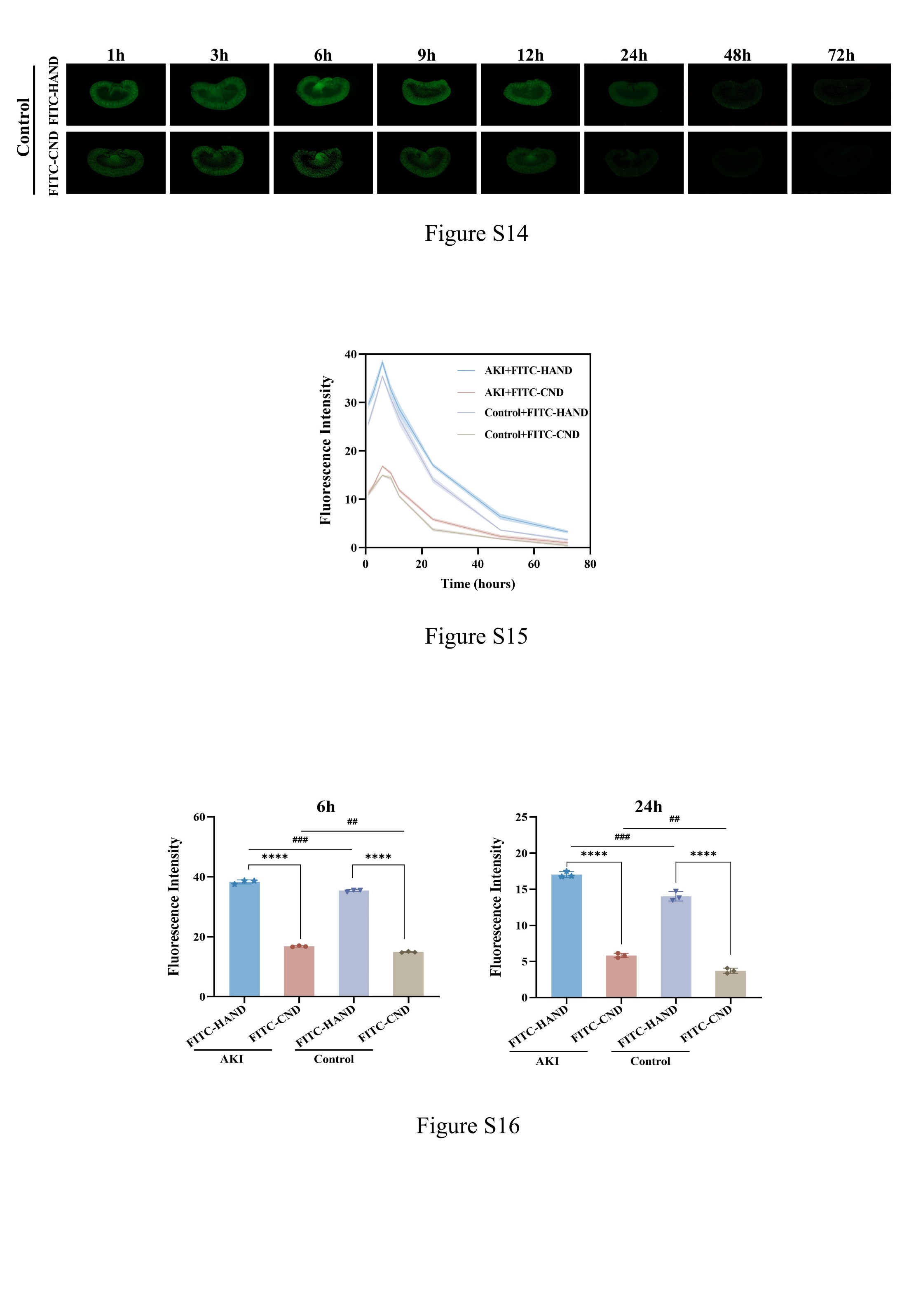


**Figure S15.** The fluorescence intensity in kidneys after drug injection (1-72h) from different groups. The average optical density of kidneys 24 hours after HAND/CND injection in different groups. Data are presented as mean ± SD. n=3.


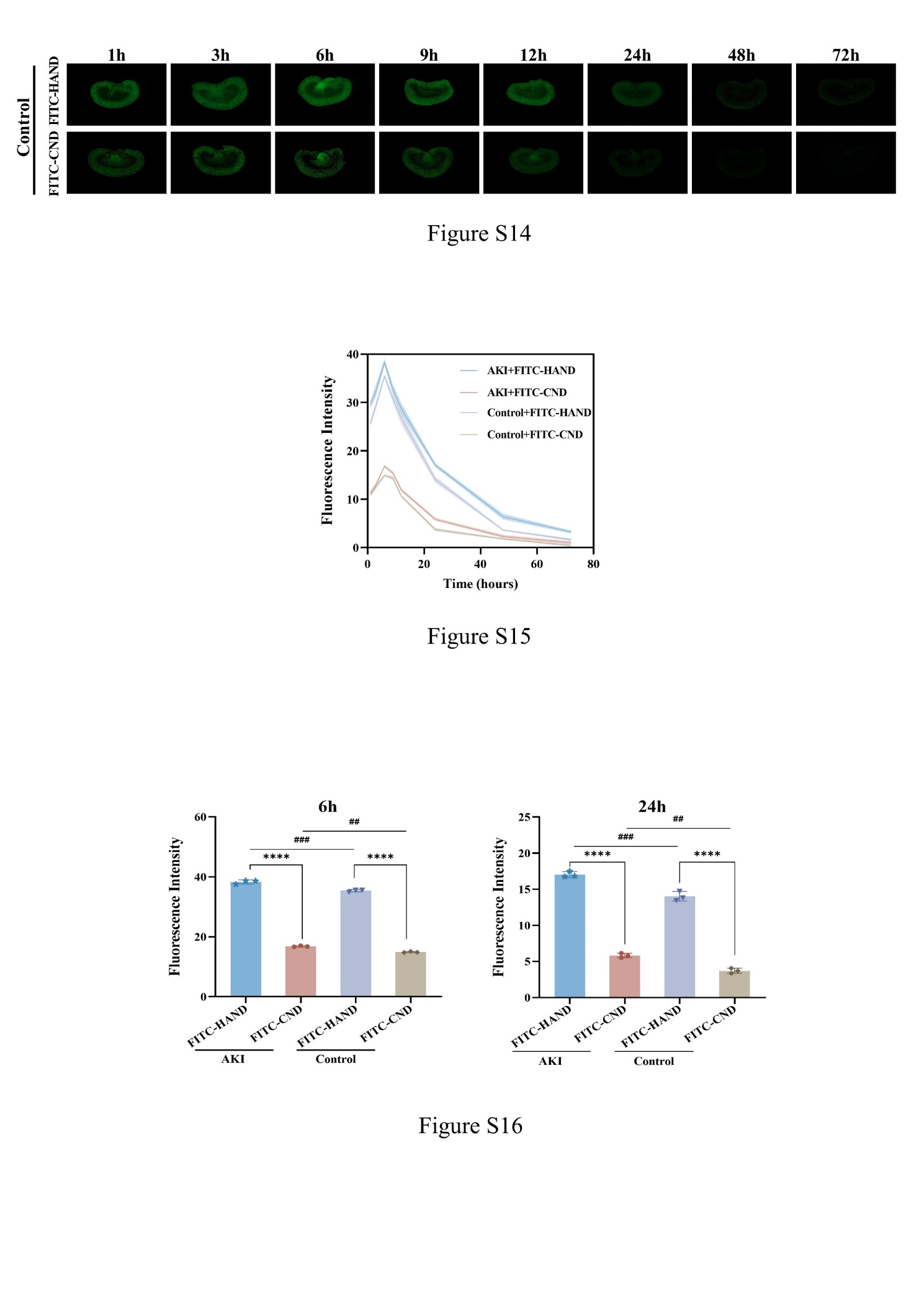


**Figure S16.** The average optical density of kidneys 6 hours and 24 hours after HAND/CND injection in different groups. Data are presented as mean ± SD. One-way ANOVA followed by SNK test was used for analysis. n=3, ^##^*P* < 0.01, ^###^*P* < 0.001, ^****^*P* < 0.0001.


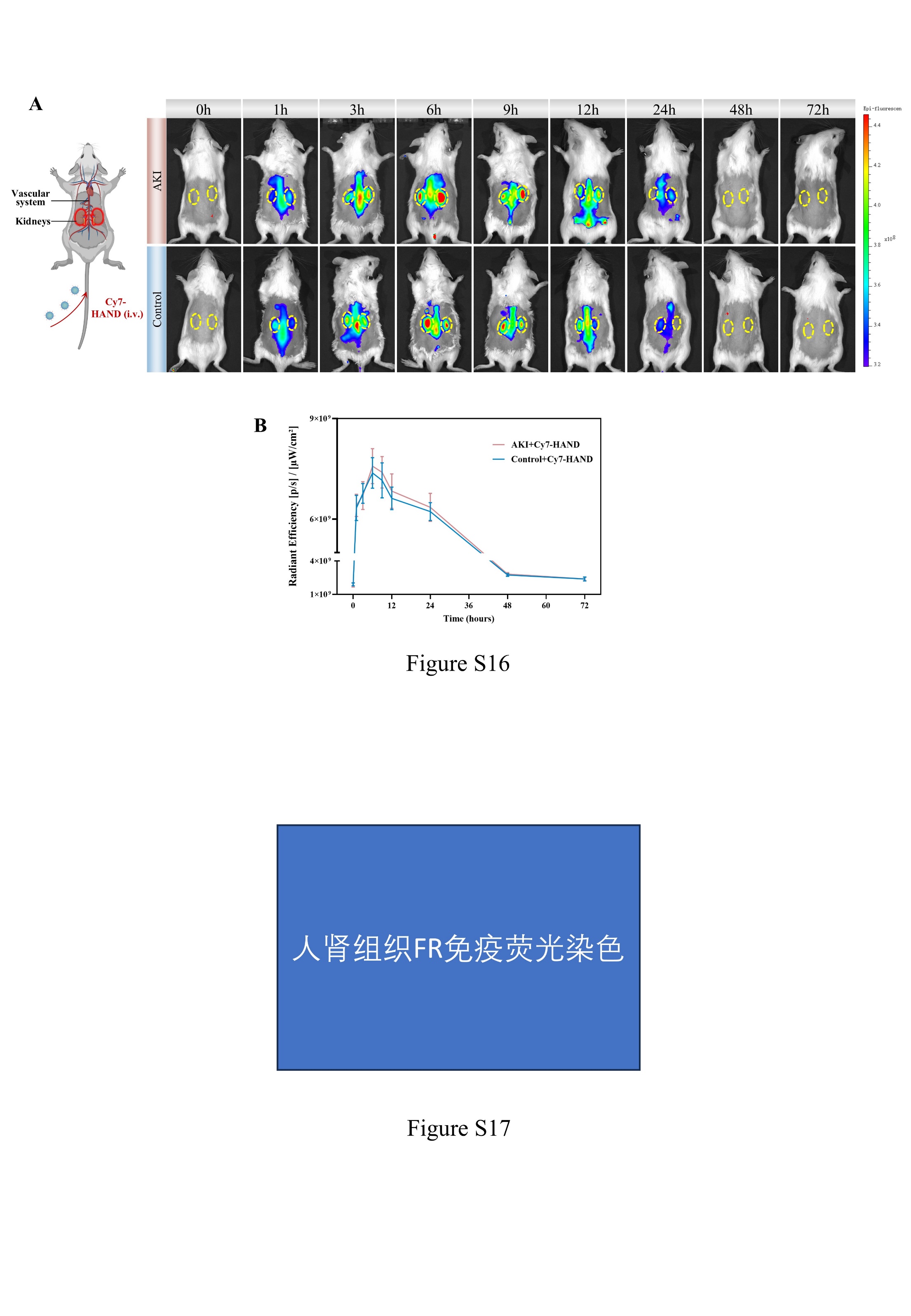


**Figure S17.** (A) Fluorescence imaging of mice at different time points after Cy7-HAND injection. (B) Quantification of fluorescence intensity in the kidney region (highlighted by yellow circles). Data are presented as mean ± SD. n=3.


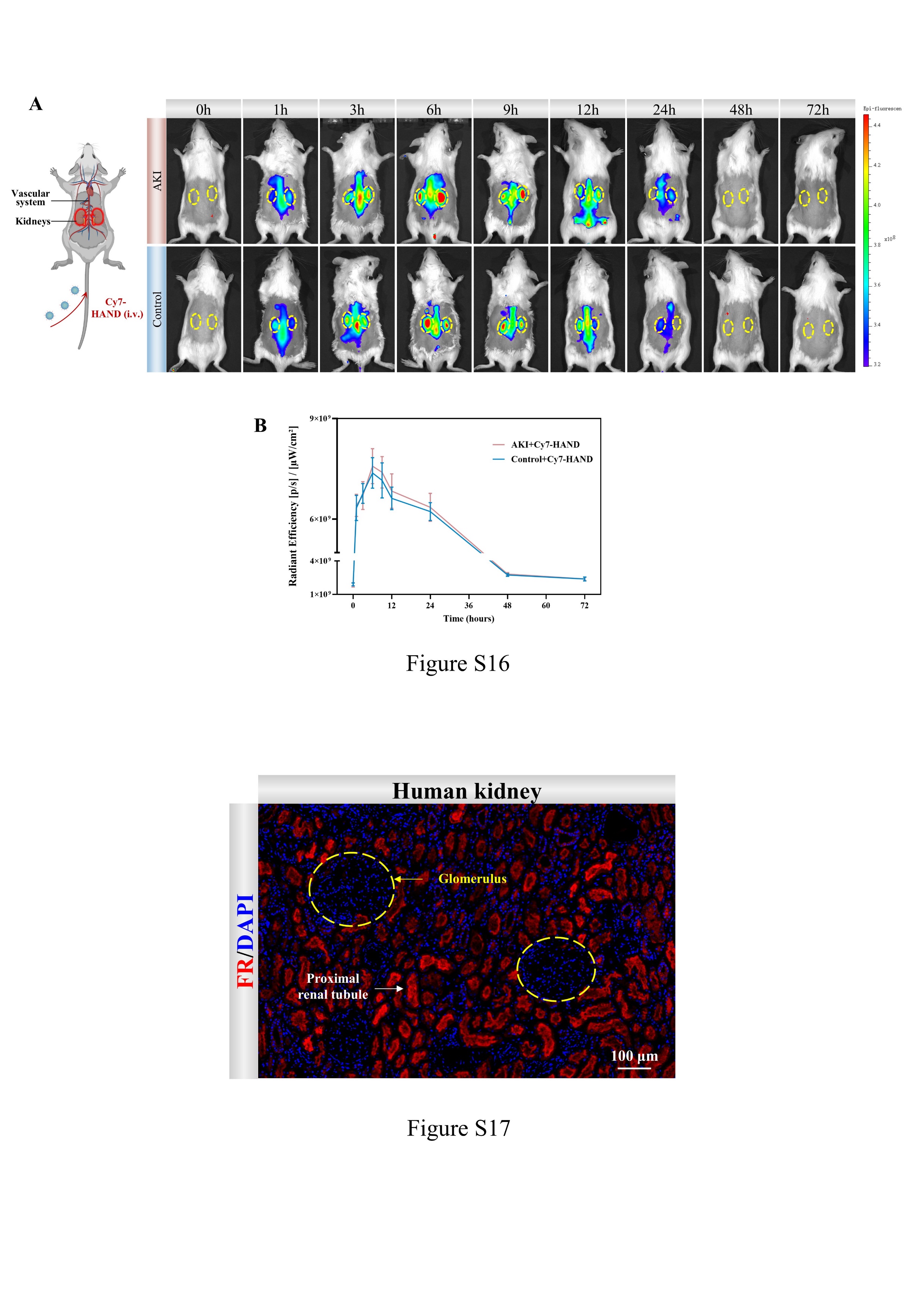


**Figure S18.** IF staining images of human kidney tissue, with yellow circles indicating glomerulus.


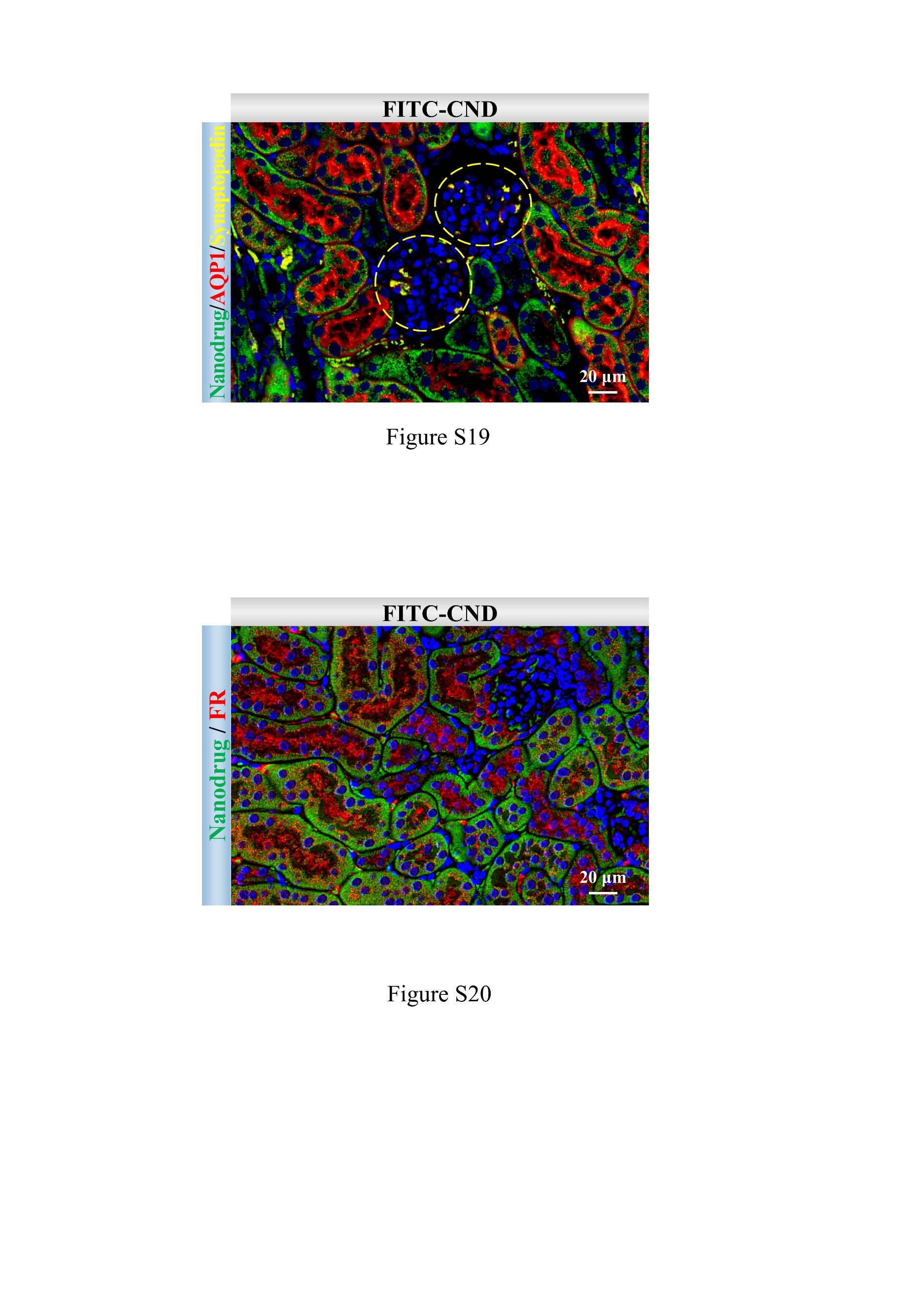


**Figure S19.** Representative IF staining images of FITC-CND with glomerular (synaptopodin) and tubular (AQP1) markers in mice kidney tissues, with yellow circles indicating glomeruli.


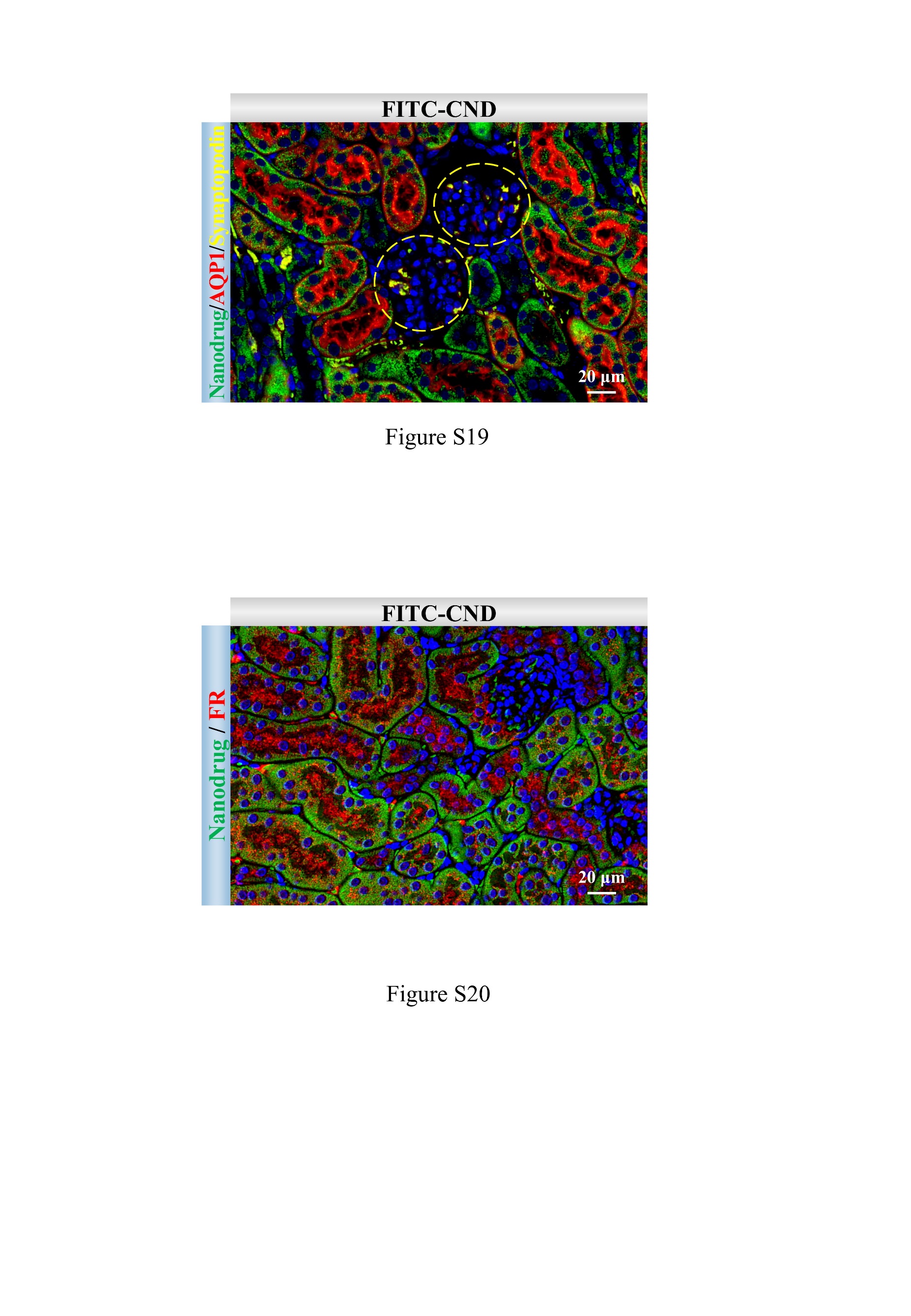


**Figure S20.** Representative IF staining image of FITC-CND with FR in mice kidney tissues.

**
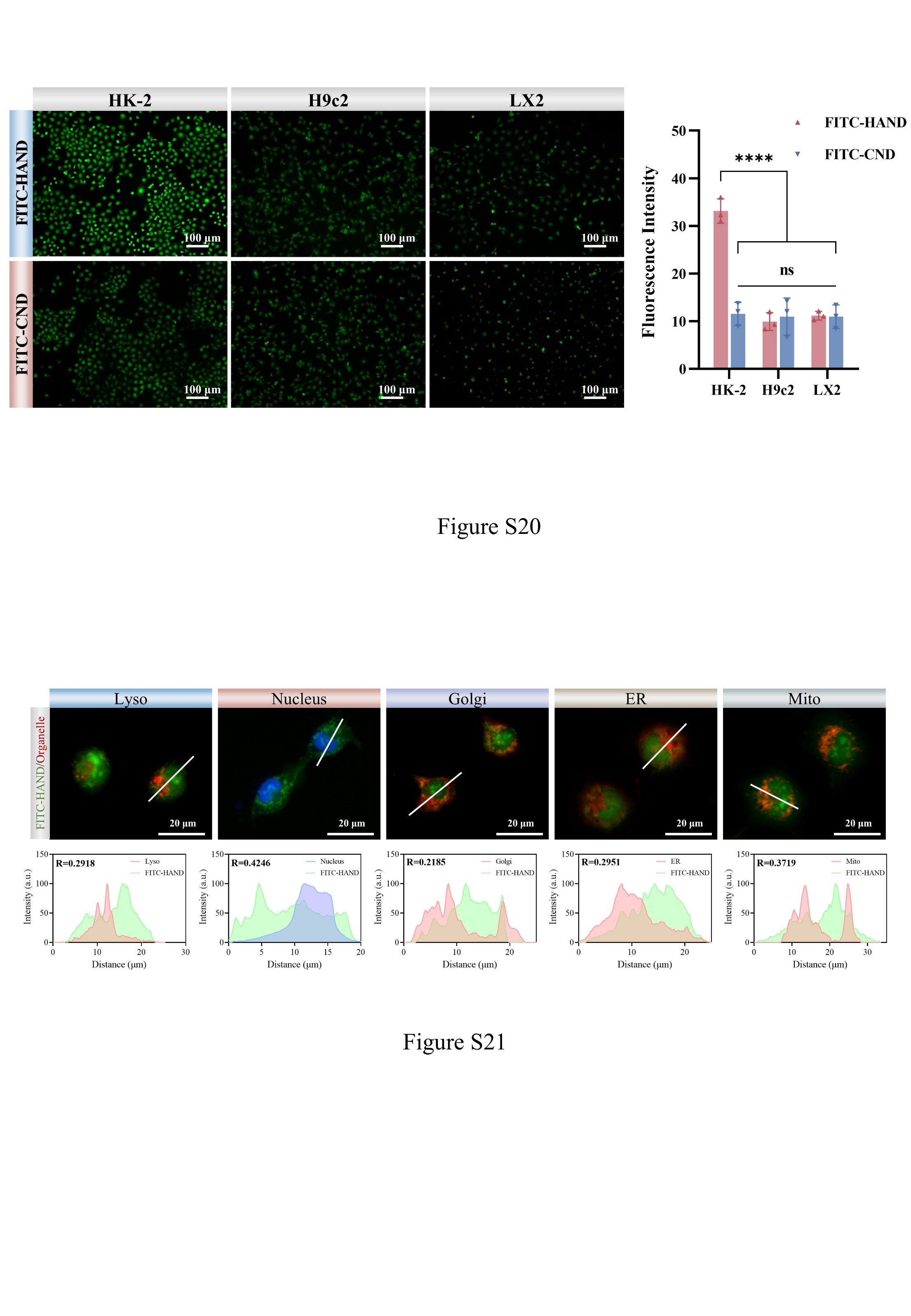
**

**Figure S21.** Representative fluorescence images and quantitative fluorescence analysis of HK-2, H9c2 and LX2 cells incubated with FITC-HAND or FITC-CND in different groups. Data are presented as mean ± SD. One-way ANOVA followed by SNK test was used for analysis. n=3, ^****^*P* < 0.0001; ns, not significant (*P* > 0.05).

**
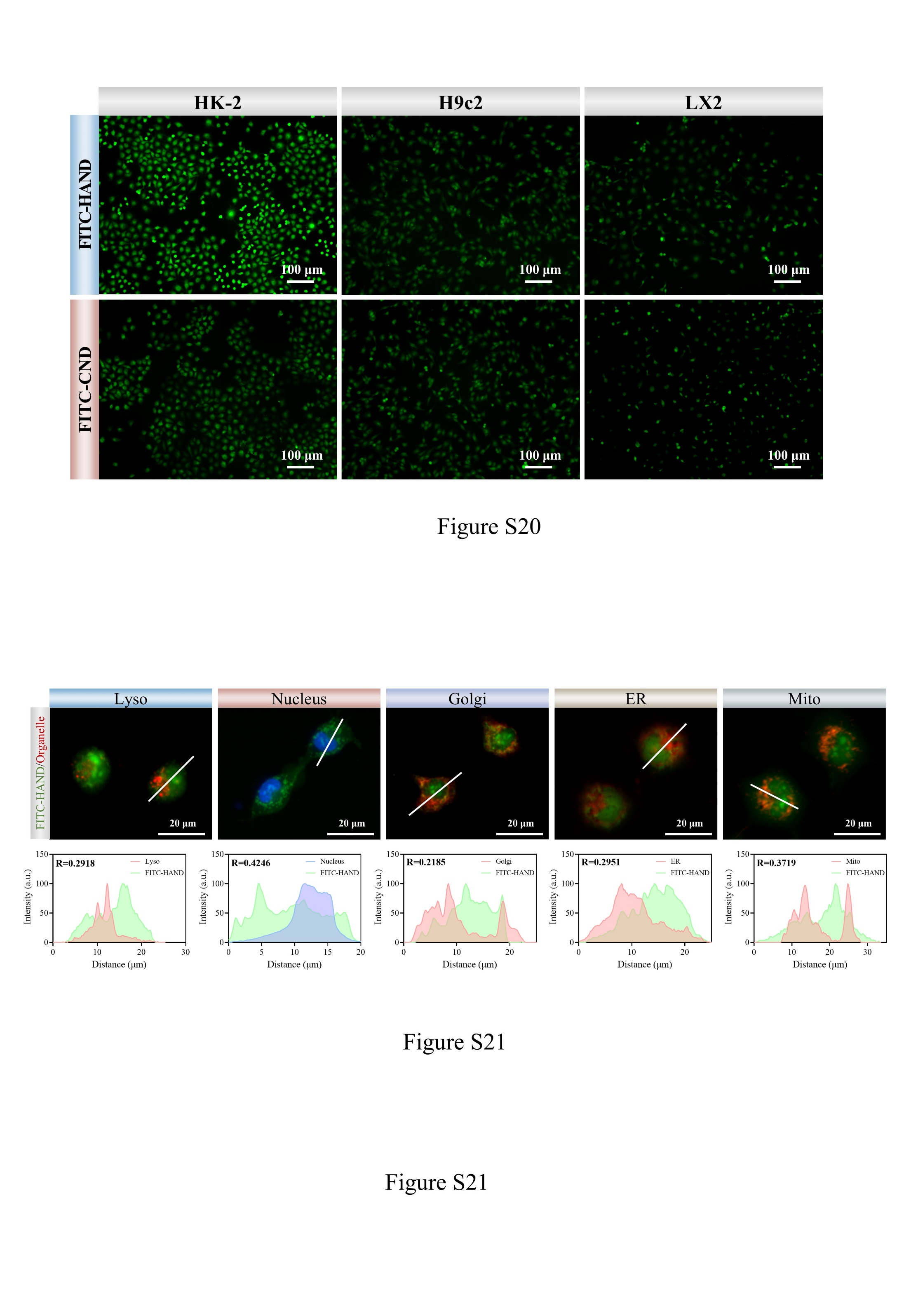
**

**Figure S22.** Colocalization fluorescence images and correlation coefficient analysis of FITC-HAND with different organelles after FR pre-treatment in HK-2 cells.


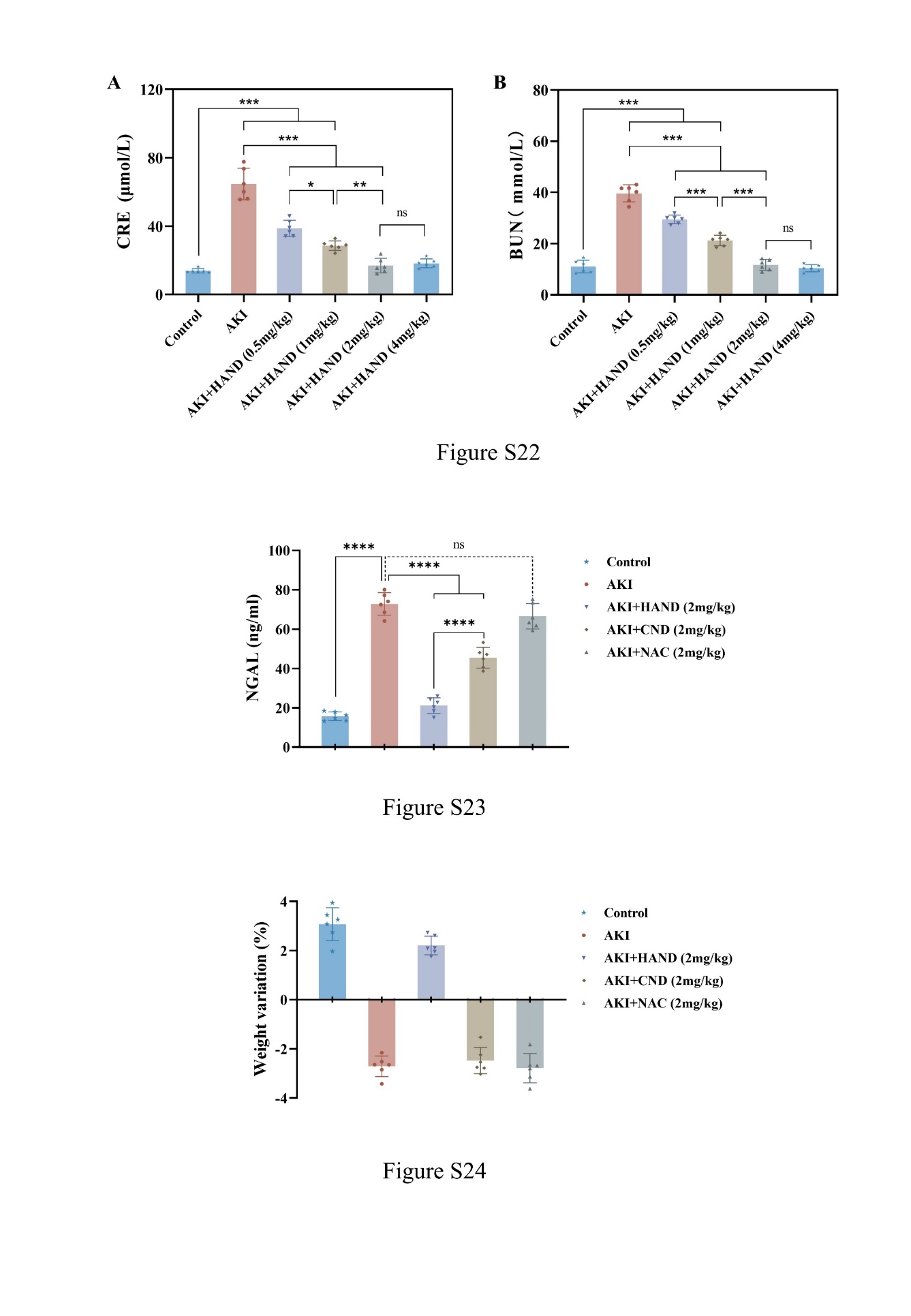


**Figure S23.** Levels of serum CRE (A) and BUN (B) in mice after injection of different doses of HAND. Data are presented as mean ± SD. One-way ANOVA followed by SNK test was used for analysis. n=6, ^*^*P* < 0.05, ^**^*P* < 0.01, ^***^*P* < 0.001; ns, not significant (*P* > 0.05).


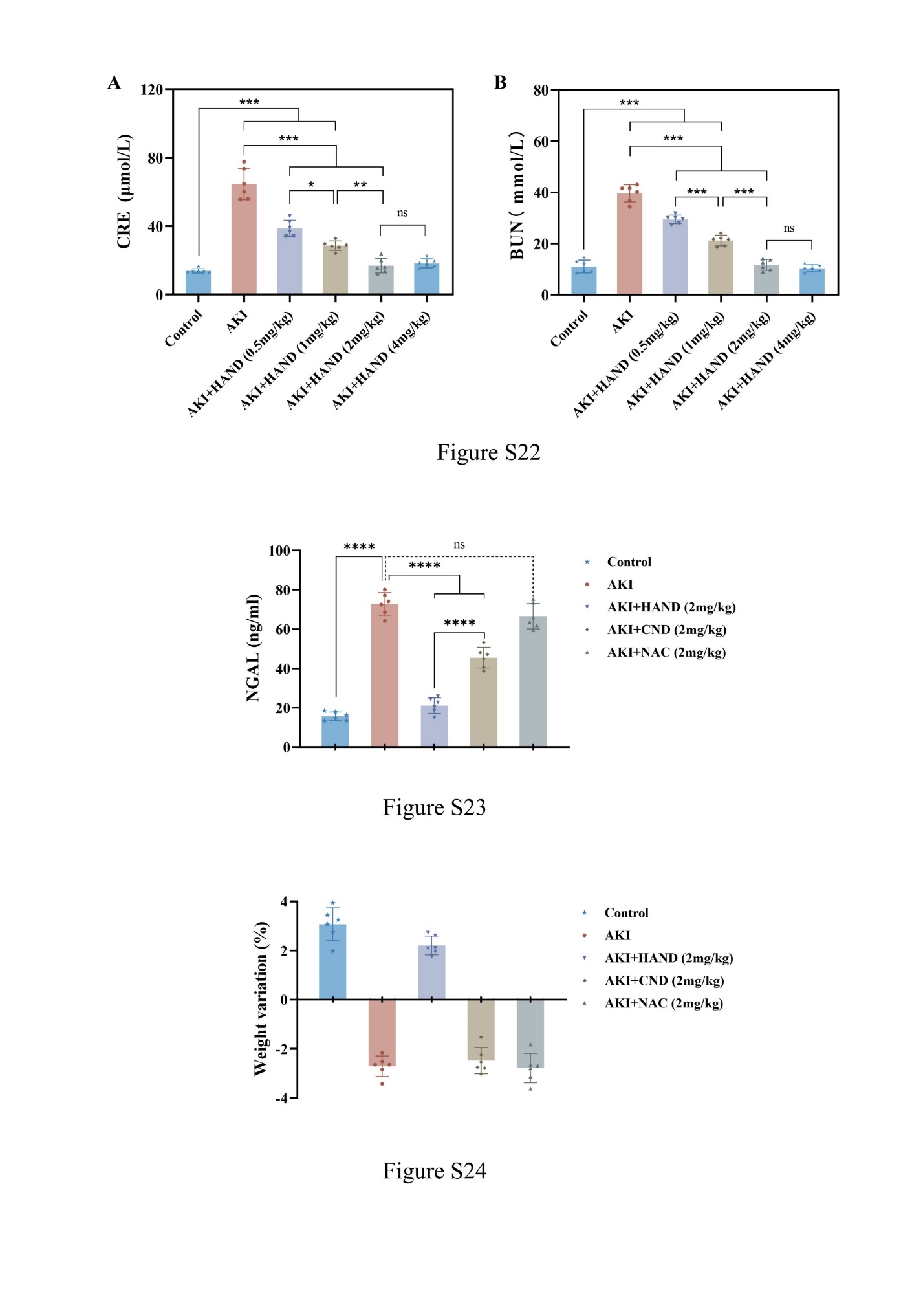


**Figure S24.** Levels of urine NGAL in different treatment groups. Data are presented as mean ± SD. One-way ANOVA followed by SNK test was used for analysis. n=6, ^****^*P* < 0.0001; ns, not significant (*P* > 0.05).


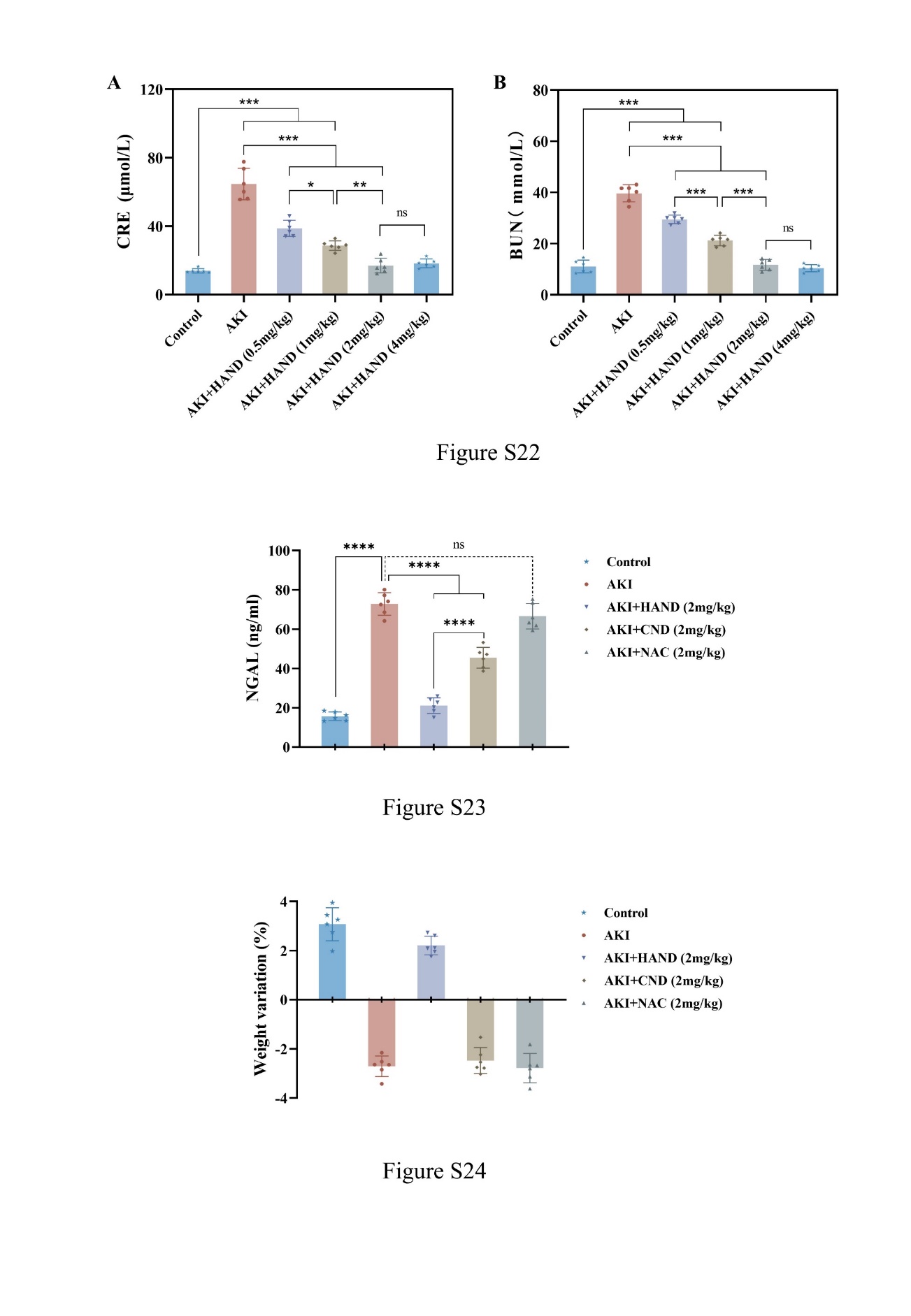


**Figure S25.** Body weight variation of different treatment groups. Data are presented as mean ± SD. n=6

**Figure S26.** Fluorescence intensity of DHE fluorescence images in different treatment groups. Data are presented as mean ± SD. One-way ANOVA followed by SNK test was used for analysis. n=6, ^****^*P* < 0.001; ns, not significant (*P* > 0.05).

**Figure S27.** Levels of 8-OHdG in kidney tissues from different treatment groups. Data are presented as mean ± SD. One-way ANOVA followed by SNK test was used for analysis. n=6, ^*^*P* < 0.05, ^**^*P* < 0.01, ^****^*P* < 0.0001; ns, not significant (*P* > 0.05).


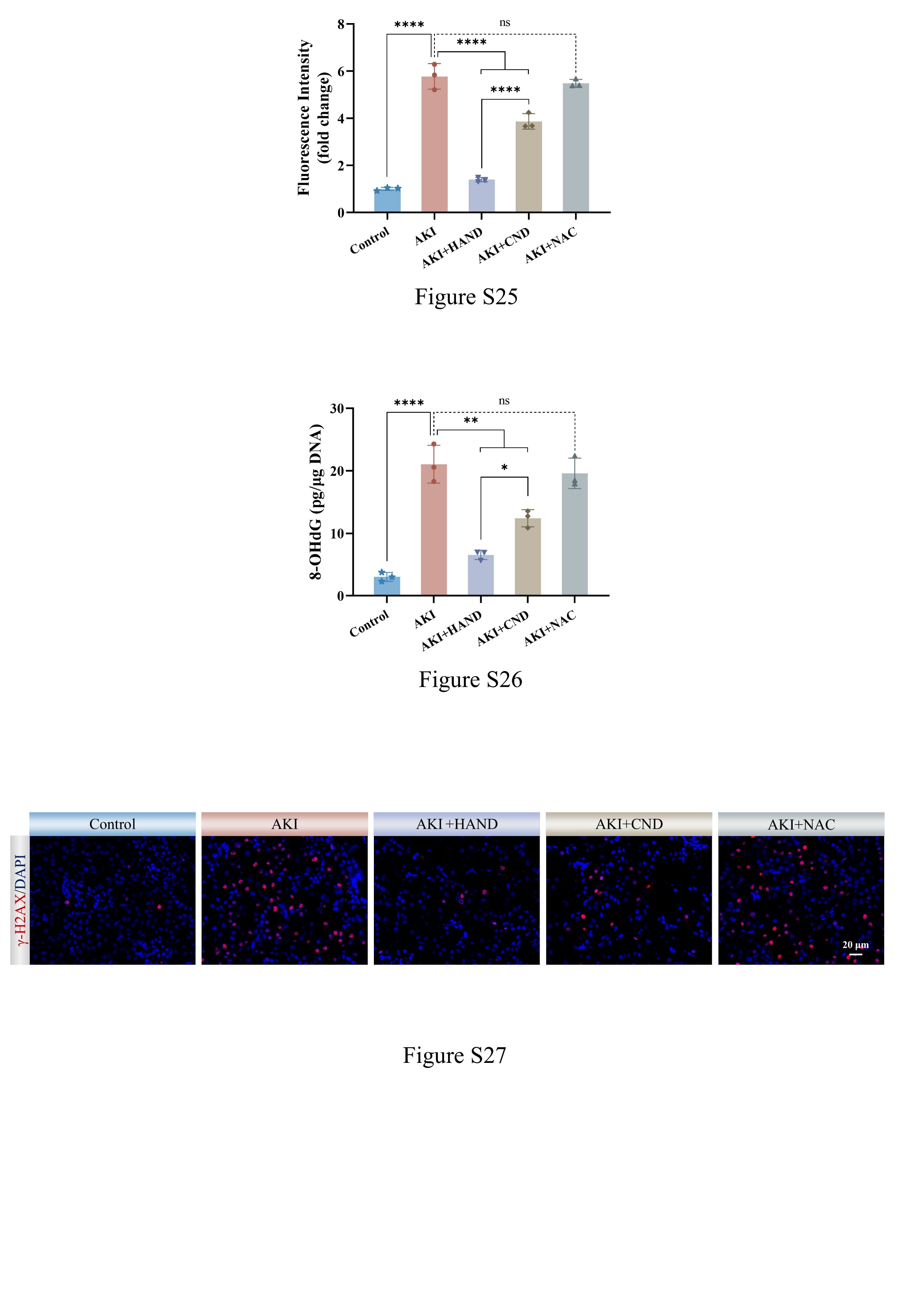


**Figure S28.** Representative γ-H2AX fluorescence images of kidney tissues in different treatment groups. n=3.


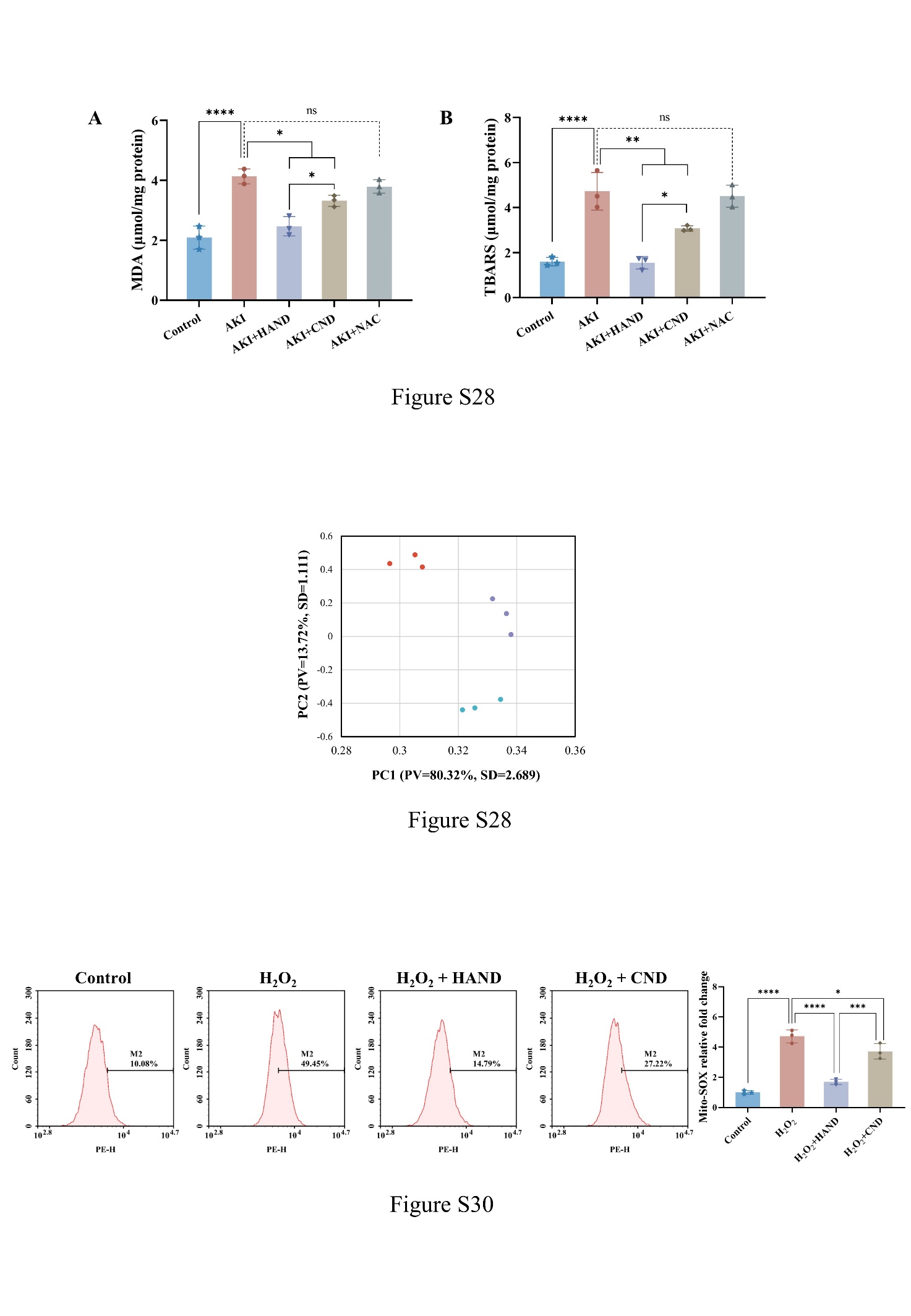


**Figure S29.** Levels of oxidative products MDA (E) and TBARS (F) in kidney tissues from different treatment groups. Data are presented as mean ± SD. One-way ANOVA followed by SNK test was used for analysis. n=6, ^*^*P* < 0.05, ^**^*P* < 0.01, ^***^*P* < 0.001.


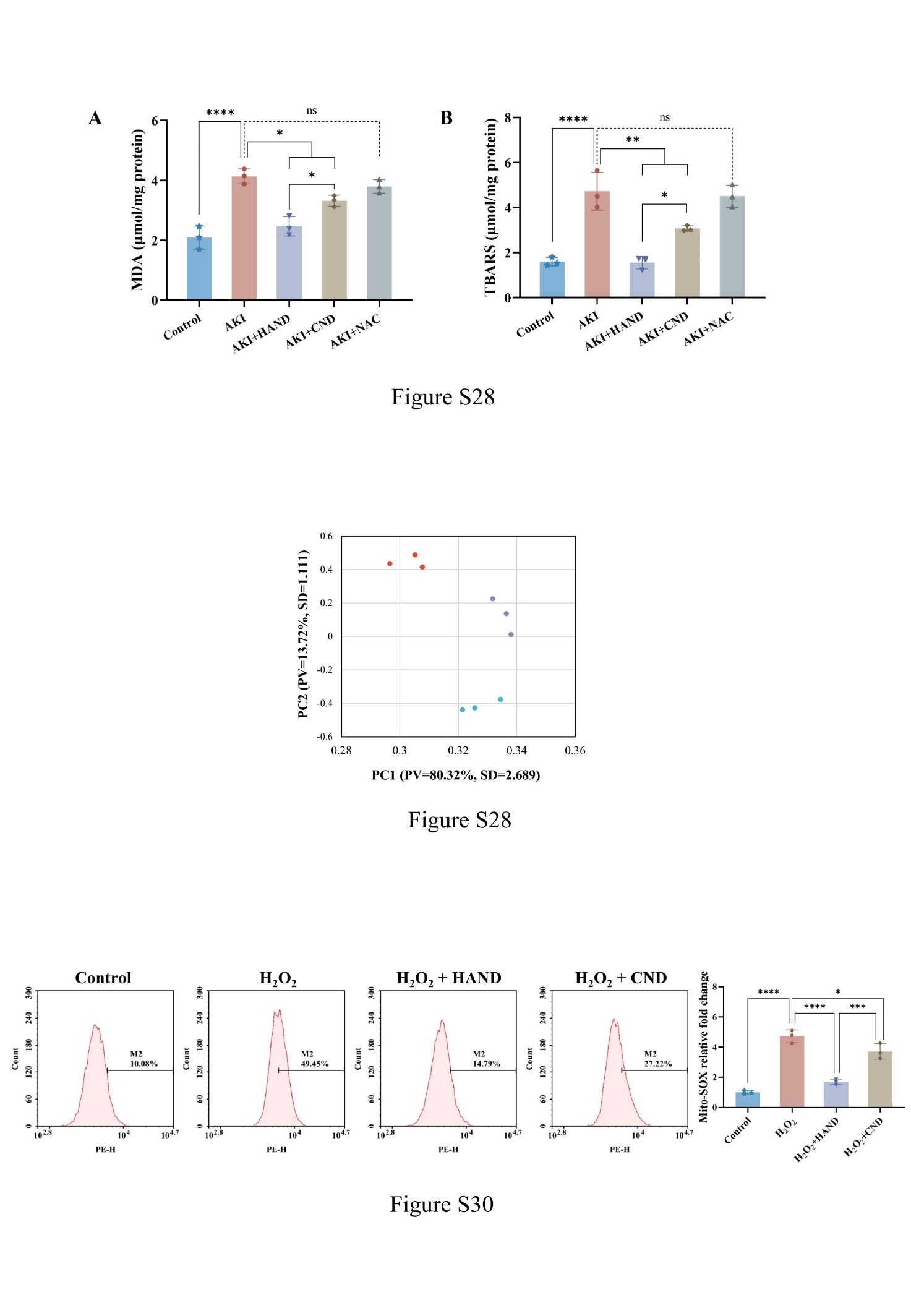


**Figure S30.** PCA results of different samples in RNA-seq of mouse kidney tissues. n=3.


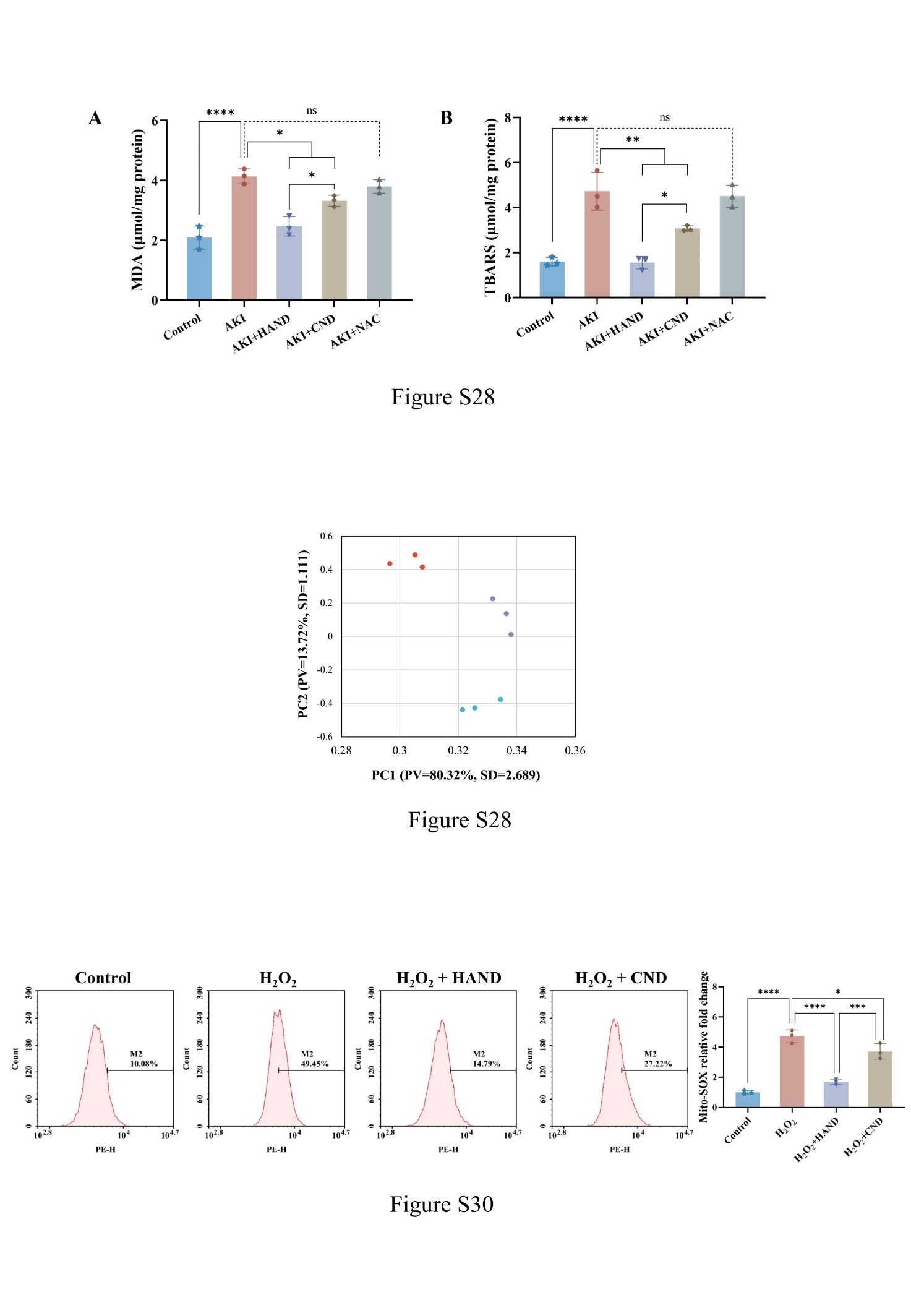


**Figure S31.** Mito-SOX flow cytometry results and statistical analysis in HK-2 cells from different treatment groups. Data are presented as mean ± SD. One-way ANOVA followed by SNK test was used for analysis. n=3, ^*^*P* < 0.05, ^***^*P* < 0.001, ^****^*P* < 0.0001.


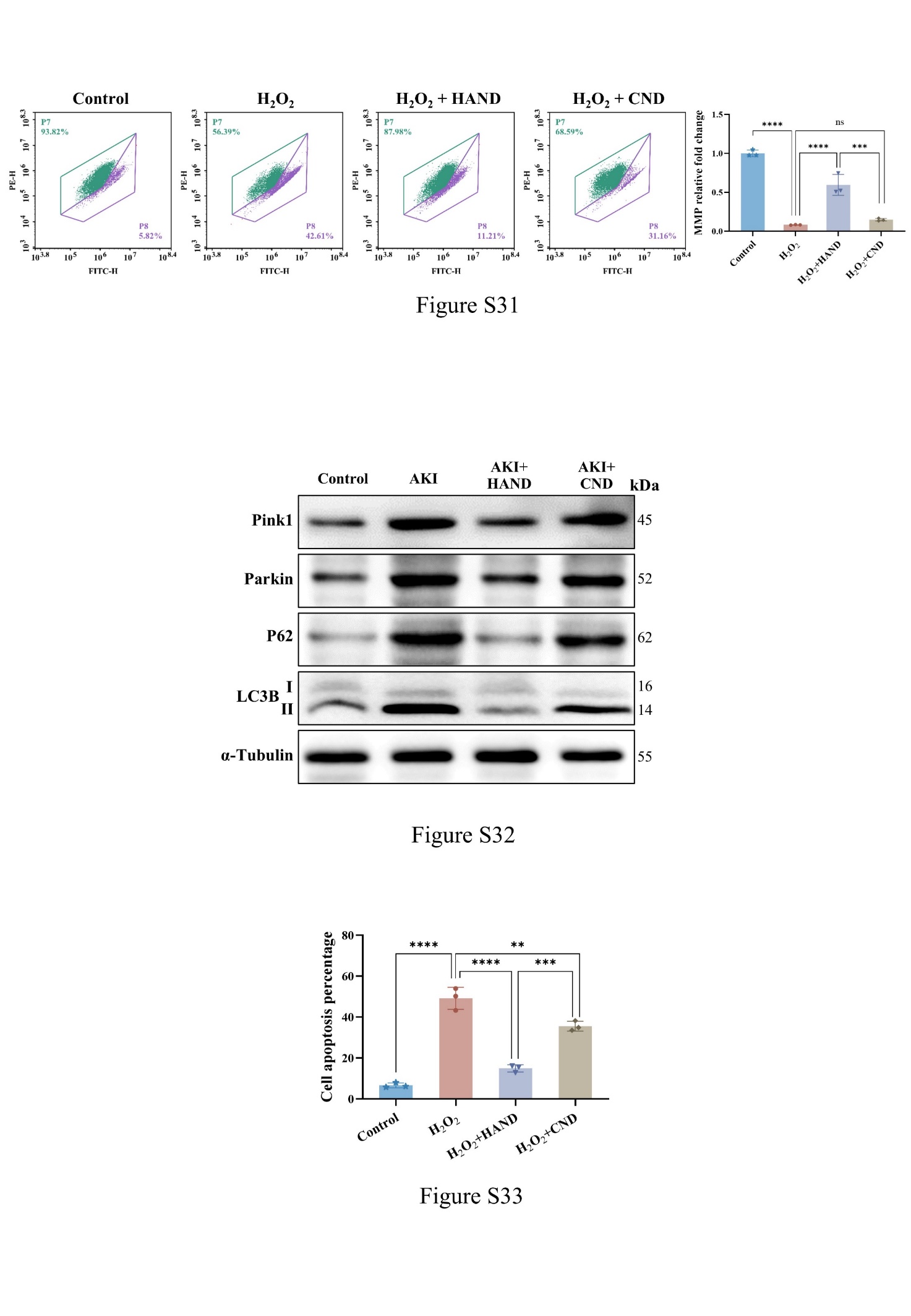


**Figure S32.** JC-1 flow cytometry results and statistical analysis in HK-2 cells from different treatment groups. Data are presented as mean ± SD. One-way ANOVA followed by SNK test was used for analysis. n=3, ^***^*P* < 0.001, ^****^*P* < 0.0001.


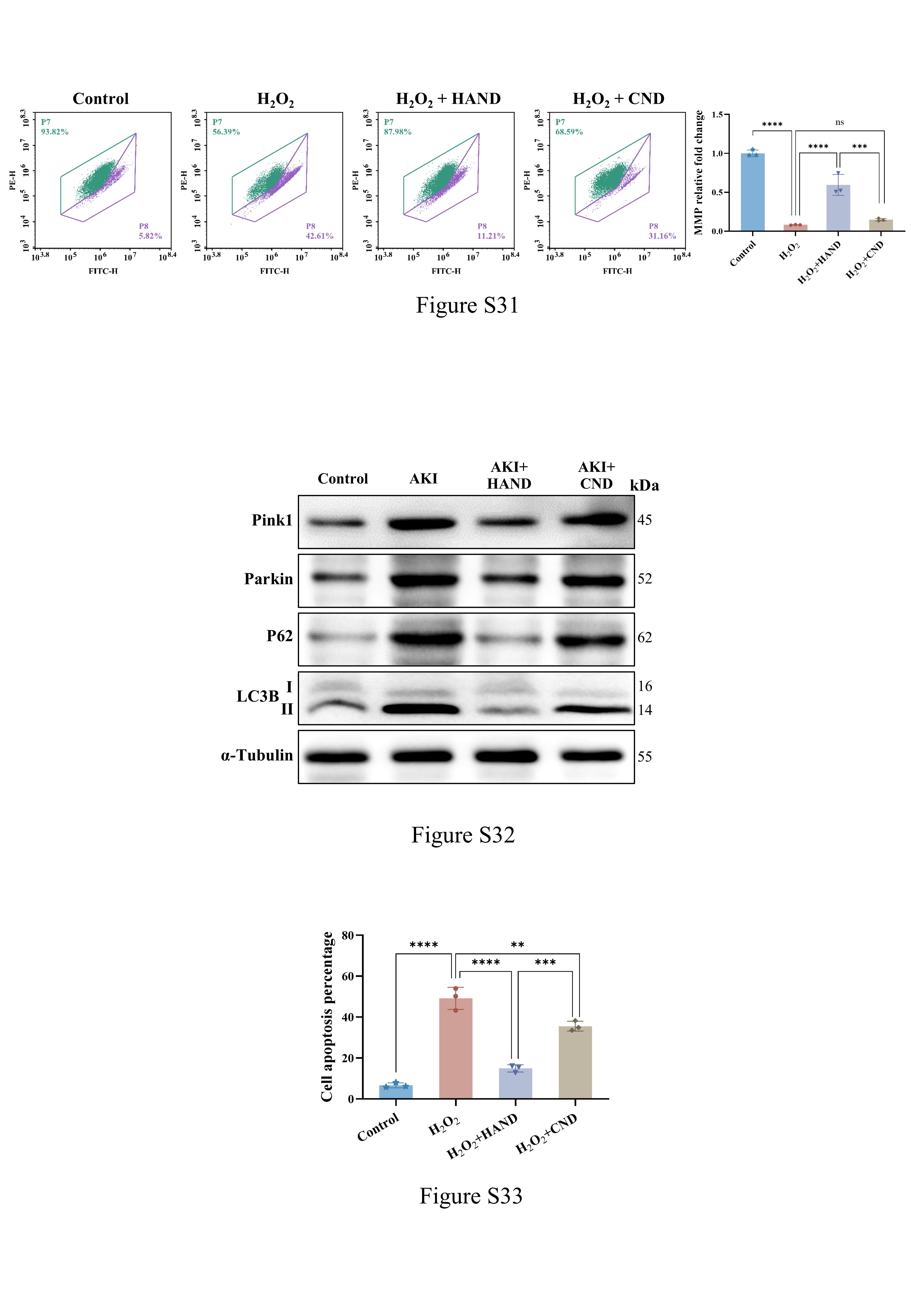


**Figure S33.** Western blot result of mitophagy-related protein expression in kidney tissues from different treatment groups of mice.


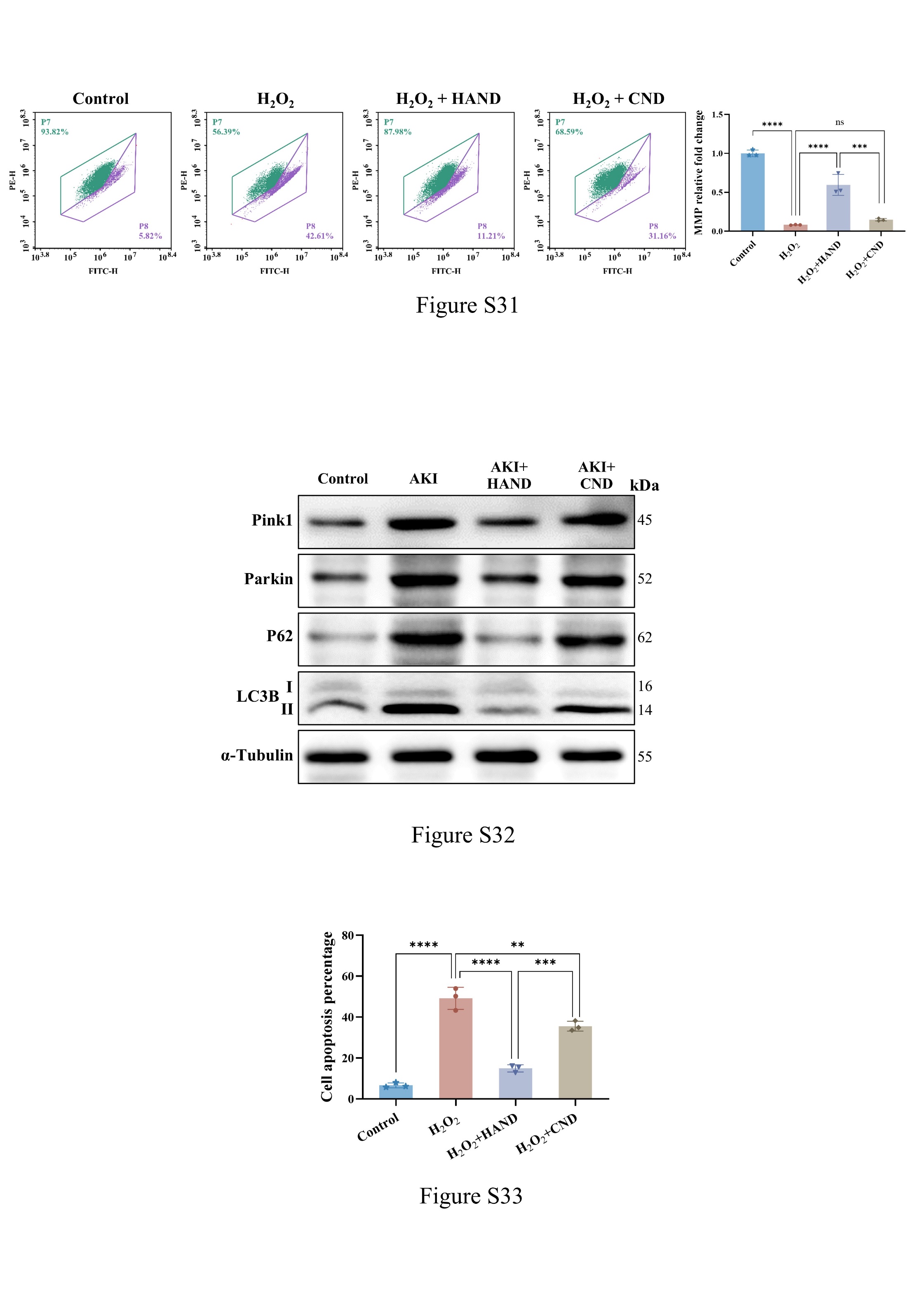


**Figure S34.** Apoptosis ratio of HK-2 cells in different treatment groups from Annexin-V/PI flow cytometry. Data are presented as mean ± SD. One-way ANOVA followed by SNK test was used for analysis. n=3, ^*^*P* < 0.05, ^**^*P* < 0.01, ^****^*P* < 0.0001.


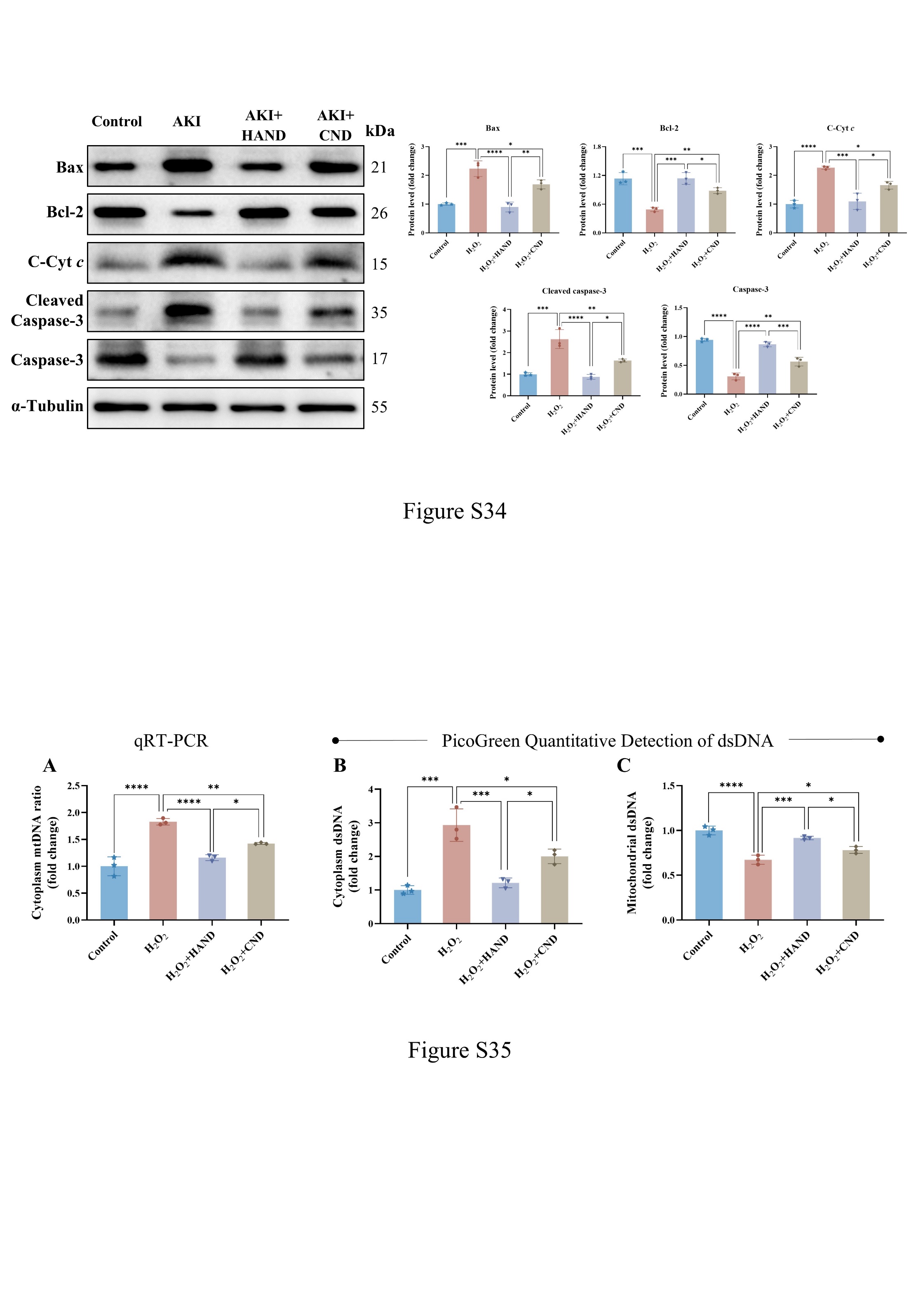


**Figure S35.** Western blot results of apoptosis-related protein expression levels in HK-2 cells from different treatment groups and grayscale analysis of Bax, Bcl-2, C-Cyt *c*, Caspase-3 and Cleaved-Caspase 3. Data are presented as mean ± SD. One-way ANOVA followed by SNK test was used for analysis. n=3, ^*^*P* < 0.05, ^**^*P* < 0.01, ^***^*P* < 0.001, ^****^*P* < 0.0001.


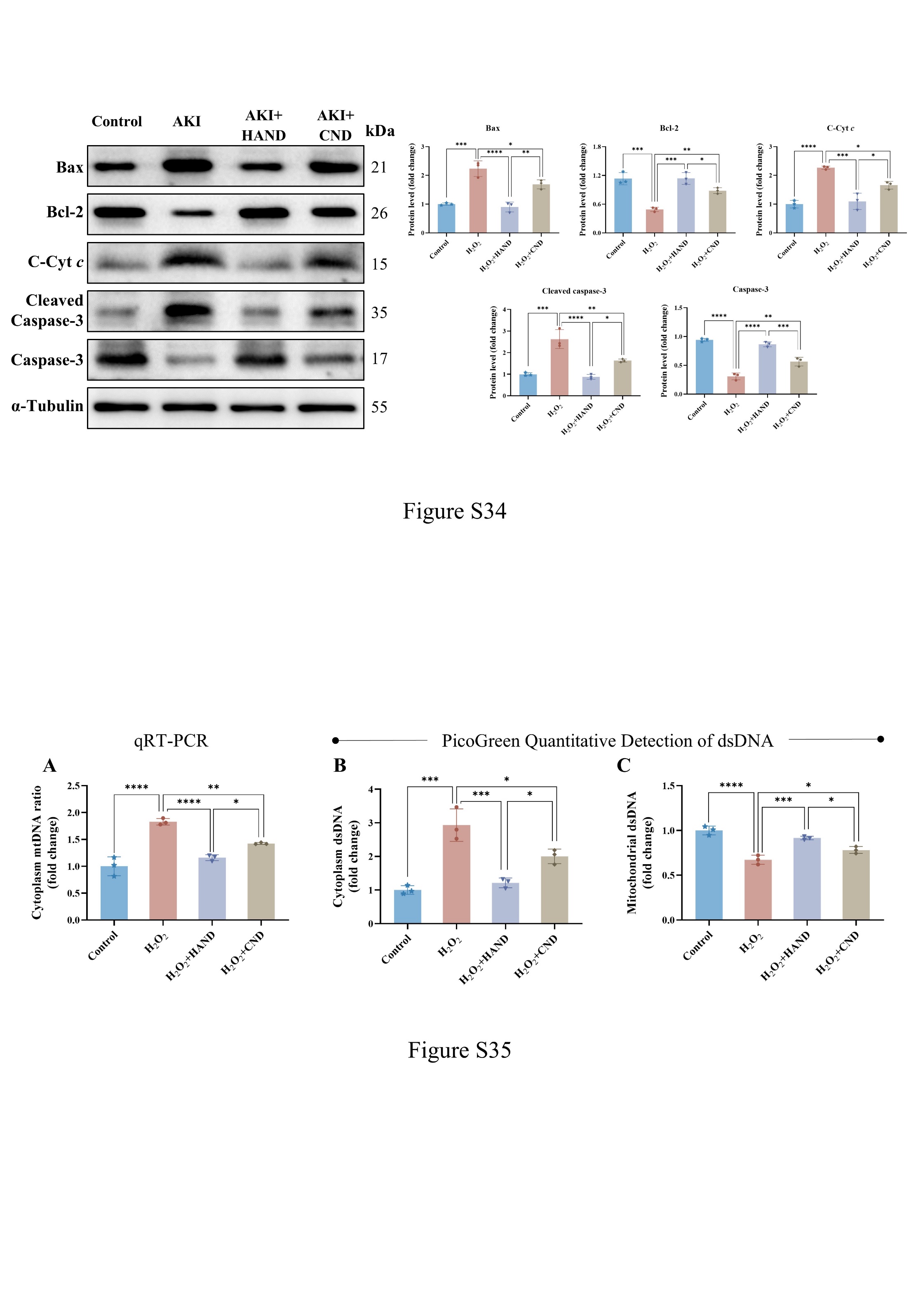


**Figure S36.** (A) mtDNA levels in HK-2 cells of different treatment groups from qRT-PCR. (B-C) dsDNA levels in cytoplasm (B) and mitochondrial (C) of HK-2 cells in different treatment groups. Data are presented as mean ± SD. One-way ANOVA followed by SNK test was used for analysis. n=3, ^*^*P* < 0.05, ^**^*P* < 0.01, ^***^*P* < 0.001, ^****^*P* < 0.0001.


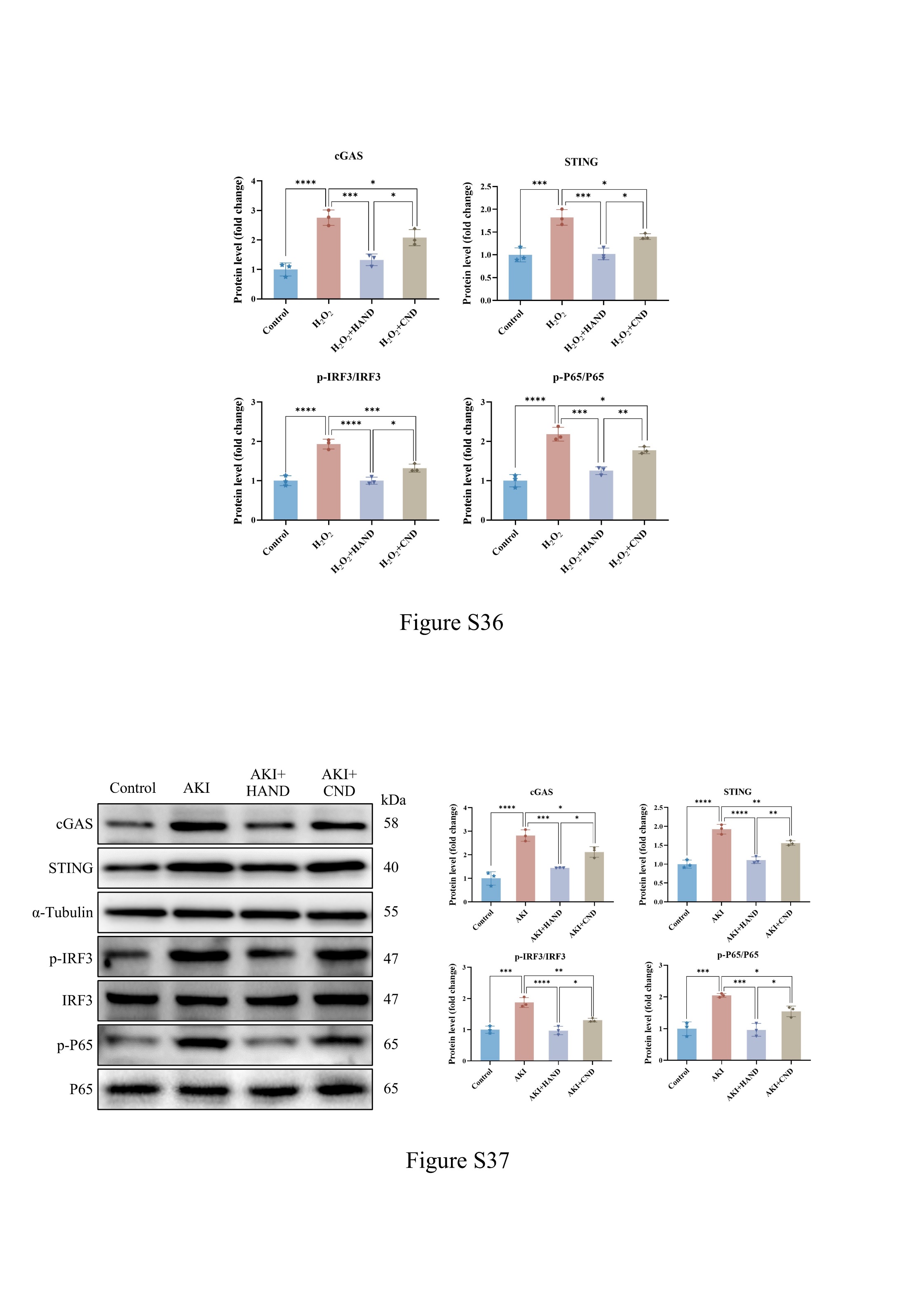


**Figure S37.** Grayscale analysis of cGAS, STING, p-IRF3/IRF3 and p-P65/P65 expression levels in HK-2 cells from Western blot. Data are presented as mean ± SD. One-way ANOVA followed by SNK test was used for analysis. n=3, ^*^*P* < 0.05, ^**^*P* < 0.01, ^***^*P* < 0.001, ^****^*P* < 0.0001.


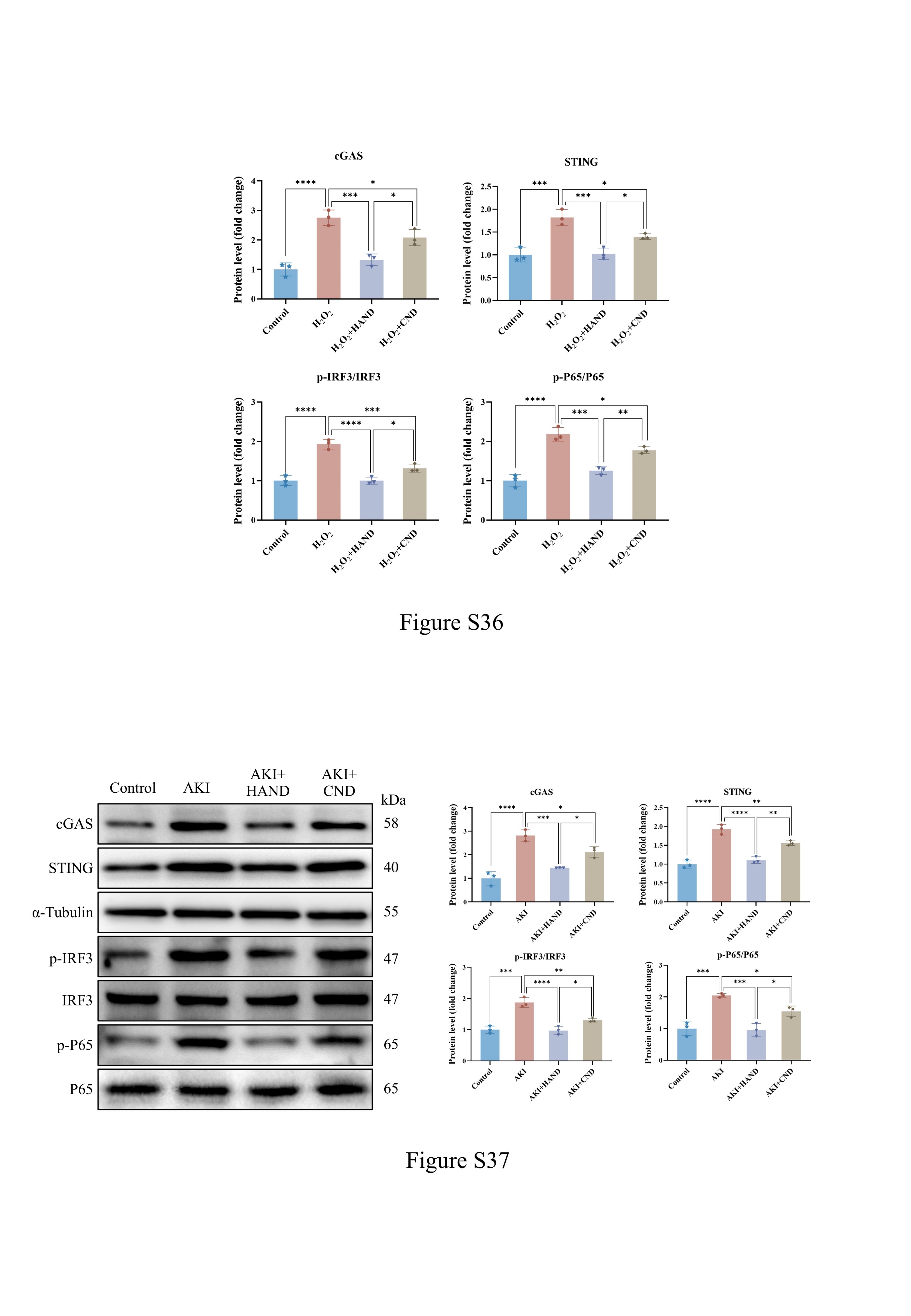


**Figure S38.** Western blot results of cGAS-STING related protein expression levels in kidneys from different treatment groups and grayscale analysis of cGAS, STING, p-IRF3/IRF3 and p-P65/P65. Data are presented as mean ± SD. One-way ANOVA followed by SNK test was used for analysis. n=3, ^*^*P* < 0.05, ^**^*P* < 0.01, ^***^*P* < 0.001, ^****^*P* < 0.0001.


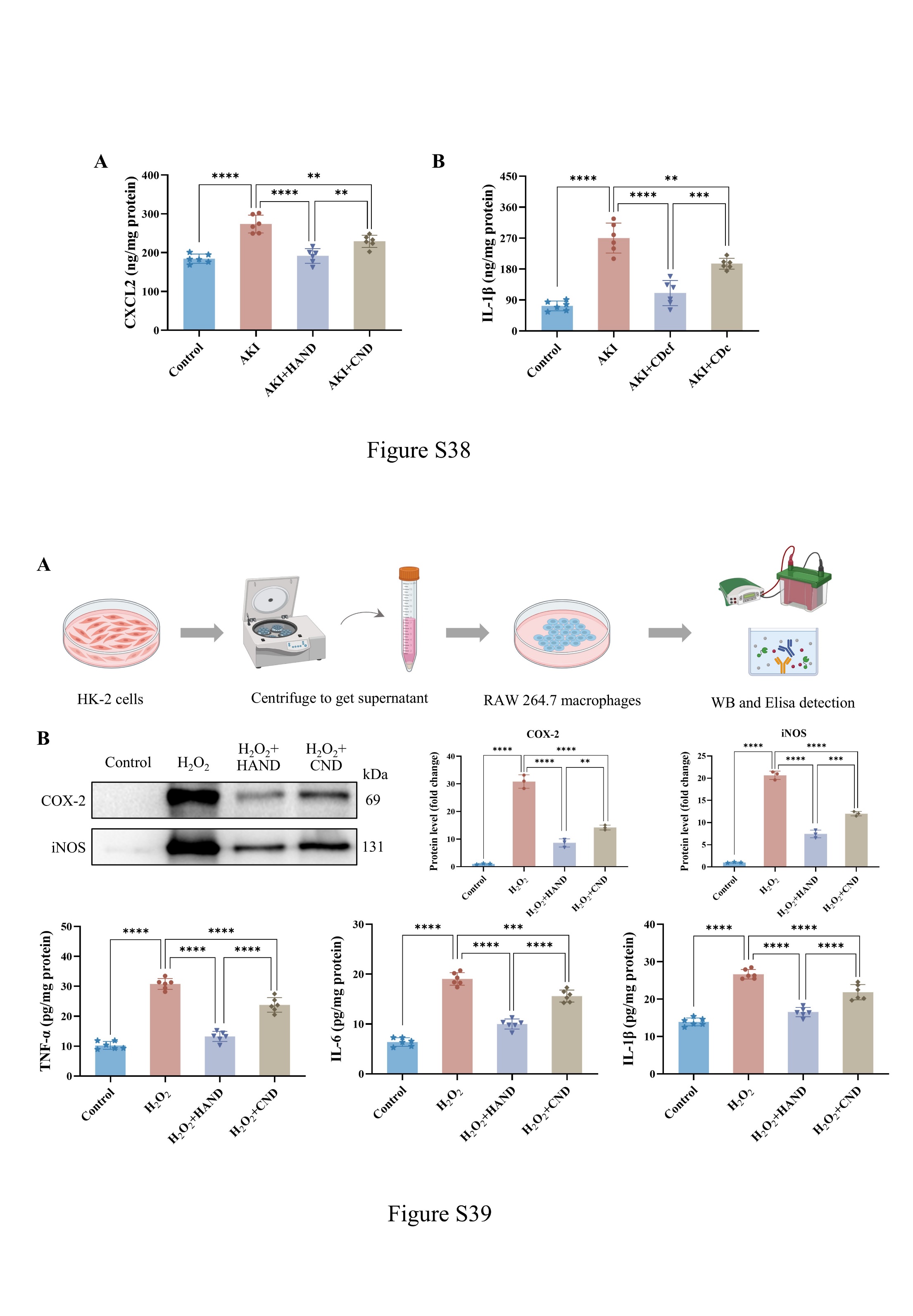


**Figure S39.** Levels of chemokines CXCL2 (A) and inflammatory cytokines IL-1β (B) in kidneys from different treatment groups. Data are presented as mean ± SD. One-way ANOVA followed by SNK test was used for analysis. n=3. ^**^*P* < 0.01, ^***^*P* < 0.001, ^****^*P* < 0.0001.


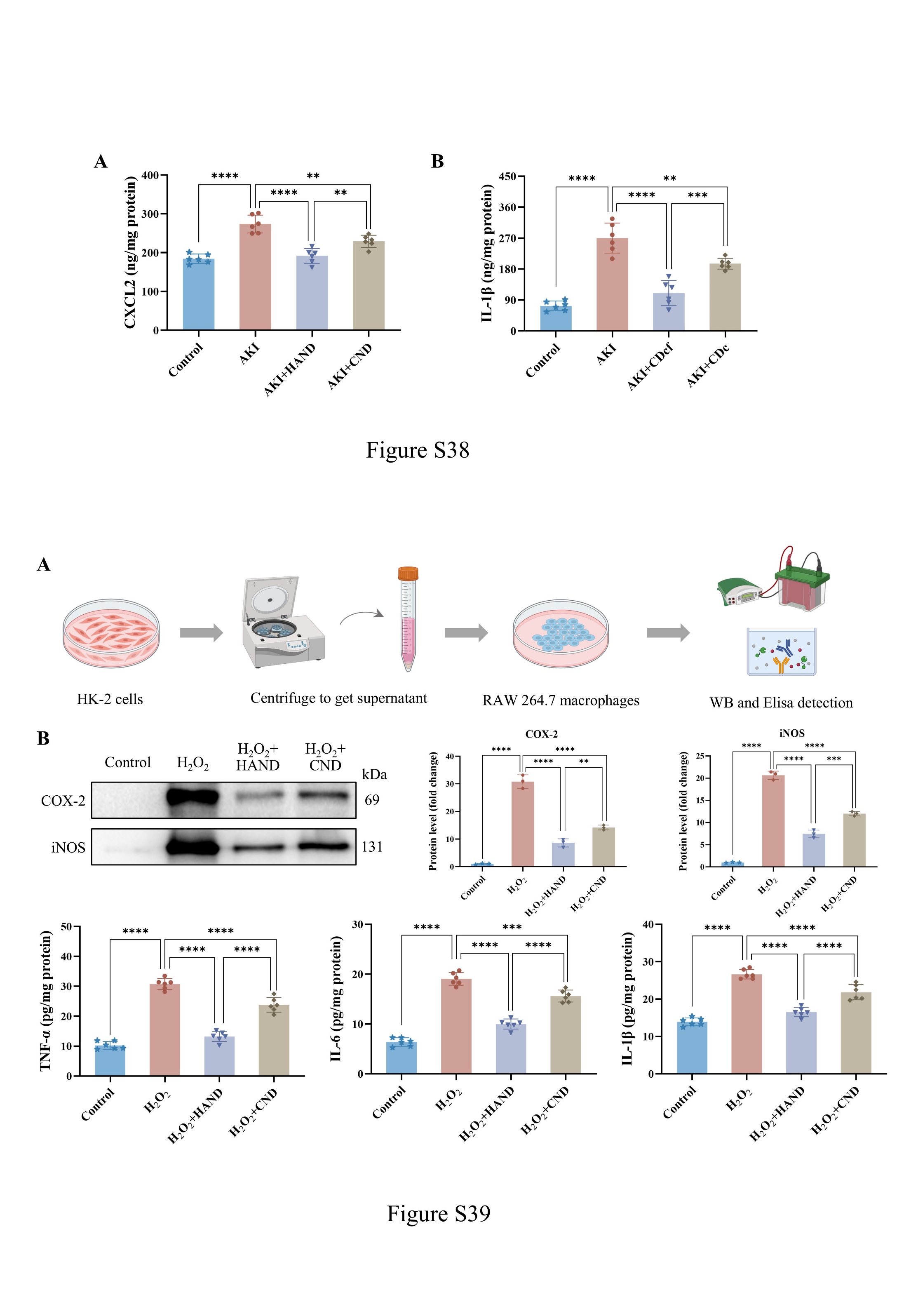


**Figure S40.** (A) Schematic illustration of RAW264.7 cells cultured in HK-2 cells conditioned medium. (B) Western blot results of COX-2 and iNOS expression levels in HK-2 cells from different treatment groups and corresponding grayscale analysis. Data are presented as mean ± SD. One-way ANOVA followed by SNK test was used for analysis. n=3. ^**^*P* < 0.01, ^***^*P* < 0.001, ^****^*P* < 0.0001.


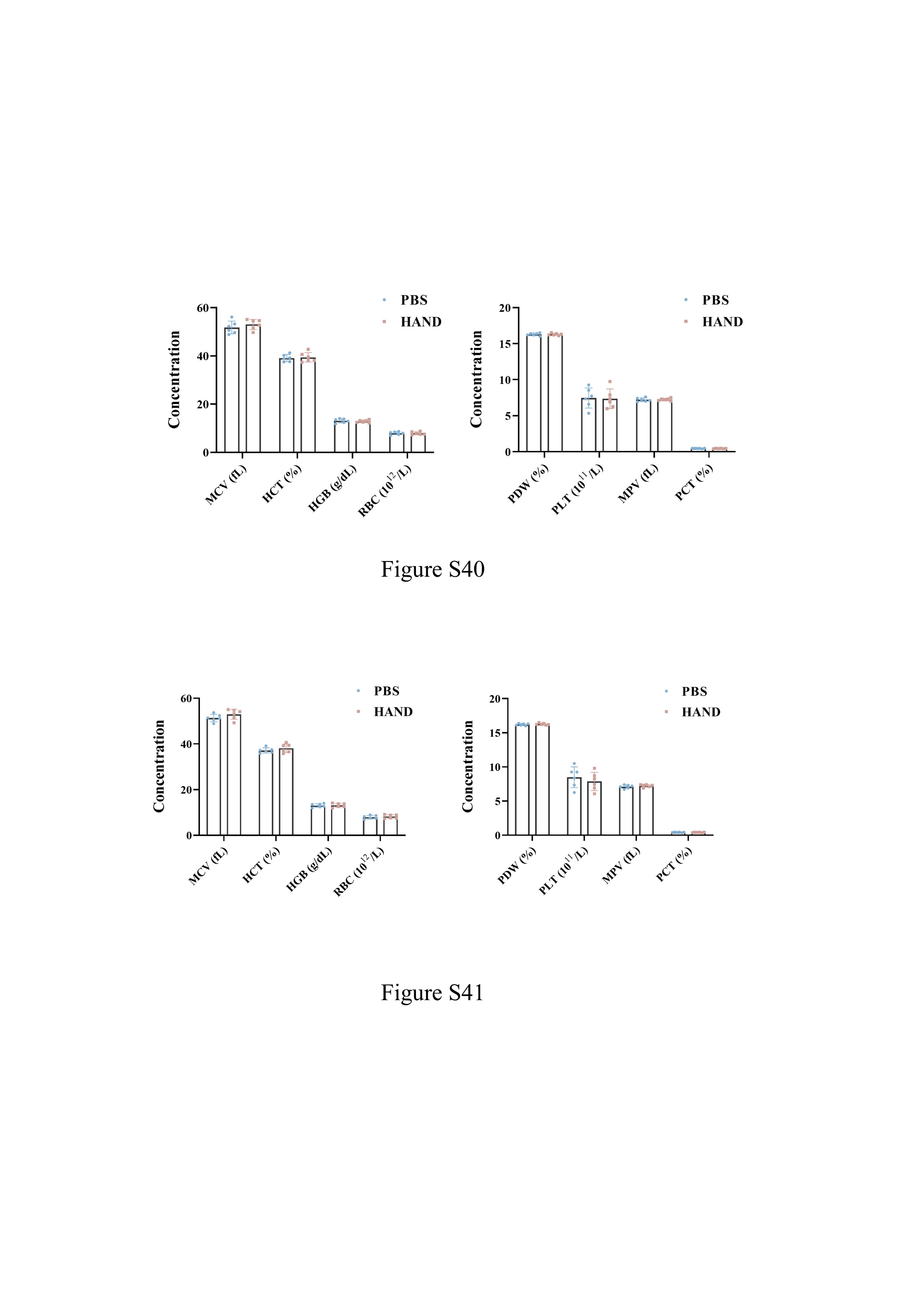


**Figure S41.** Hematological parameters of mice in different treatment groups after short-term HAND/PBS treatment. Data are presented as mean ± SD. n=6.


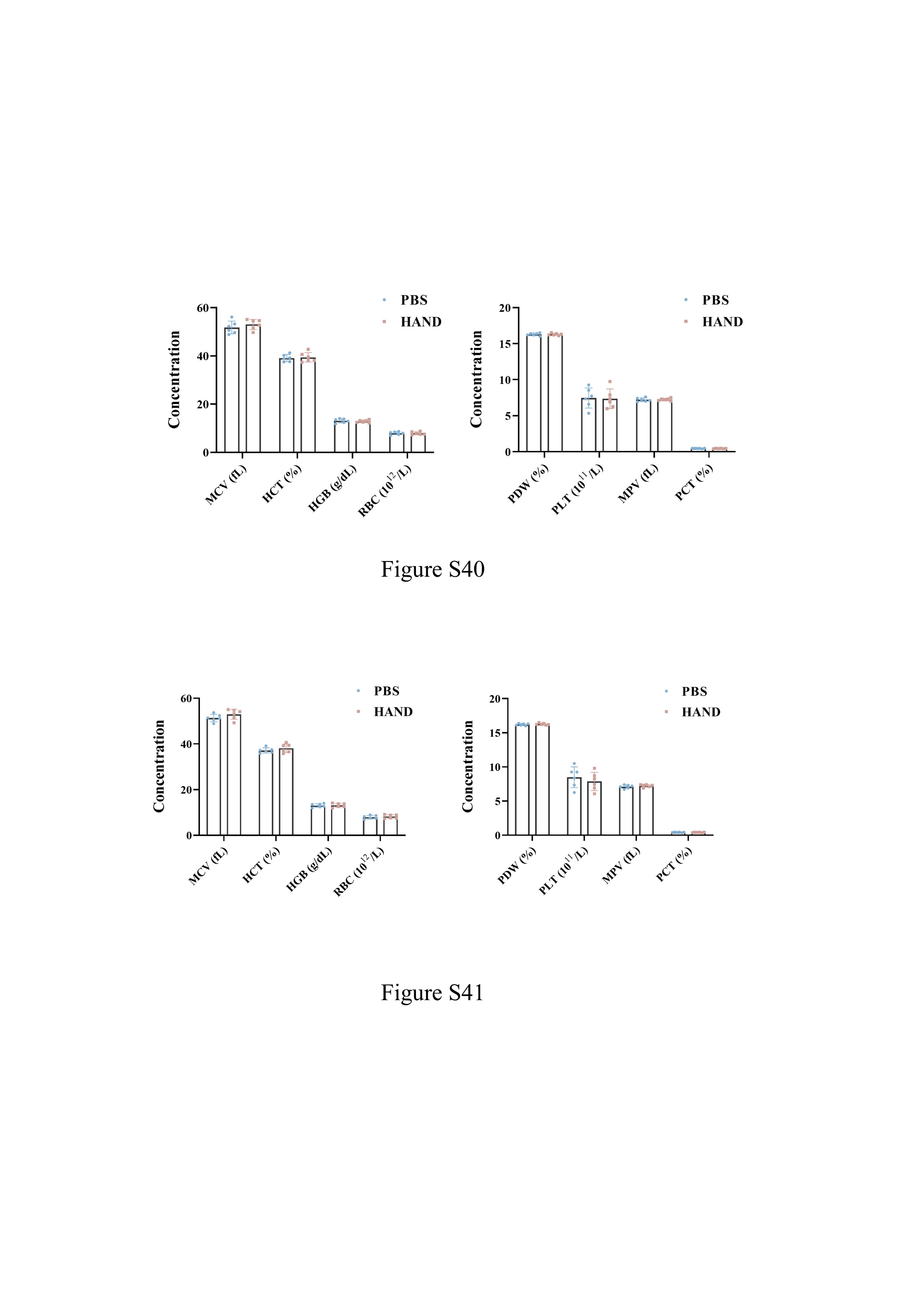


**Figure S42.** Hematological parameters of mice in different treatment groups after long-term HAND/PBS treatment. Data are presented as mean ± SD. n=6.
